# Molecular dynamics guided all-atom reconstruction of cryo-ET maps reveals mechanisms of histone tail mediated chromatin compaction

**DOI:** 10.1038/s41467-026-77871-9

**Published:** 2026-09-15

**Authors:** Shuxiang Li, Maria J. Aristizabal, Sergei A. Grigoryev, Anna R. Panchenko

**Affiliations:** 1 Department of Pathology and Molecular Medicine, Queen’s University, Kingston, ON Canada; 2 Biology Department, Queen’s University, Kingston, ON Canada; 3 Department of Biomedical and Molecular Sciences, Queen’s University, Kingston, ON Canada; 4 Department of Molecular and Precision Medicine, College of Medicine, Penn State University, Hershey, PA USA; 5 School of Computing, Queen’s University, Kingston, ON Canada; 6 Department of Physics, Engineering Physics & Astronomy, Queen’s University, Kingston, ON Canada

**Keywords:** Computational biophysics, Computational models, Molecular modelling

## Abstract

Dynamics and physical state of chromatin are crucial in regulating gene expression, DNA replication, and repair. Intrinsically disordered histone tails were previously recognized as key modulators of chromatin states. However, detailed atomistic mechanisms by which histone tail dynamics are associated with chromatin compaction and higher-order chromatin organization remain poorly understood. In this work, we combine extensive all-atom molecular dynamics simulations of tri-nucleosomes with varying linker lengths and cryo-electron tomography (cryo-ET) of native nucleosome arrays. Our approach offers distinct advantages as it elucidates realistic inter-nucleosomal interactions and tri-nucleosome orientations derived from physics-based MD simulations, enabling a more accurate and physically grounded interpretation of cryo-ET data. The results reveal that tri-nucleosome models can be successfully used for fitting and the near-atomistic interpretation of cryo-ET density maps of native human chromatin. Moreover, histone tails were shown to promote chromatin compaction via three major patterns: through histone-DNA interactions, histone H2A-H4 and H3-H4 tail-tail interactions. Notably, the distributions of MD-generated structural parameters of tri-nucleosomes were found to be in strong agreement with those of experimental condensed chromatin arrays.

## Abstract (web summary)

This study combines molecular dynamics simulations with cryo-electron tomography to reconstruct native chromatin structures and reveal how histone tails interact with DNA and each other to promote chromatin compaction.

## Introduction

Chromatin, a nucleoprotein complex in which genomic DNA is packaged with histones and diverse non-histone proteins and associated RNAs, plays a central role in regulating cellular processes, such as gene expression, DNA replication, transcription, recombination, and DNA repair. The dynamic folding and unfolding of chromatin are essential for maintaining its functional state, with histone N- and C-terminal tails playing a central role in these processes^1,2^. Histone tails, which are intrinsically disordered protein regions, participate in various interactions that facilitate chromatin compaction, the higher-order organization of chromatin fiber and stability^3–5^.

Understanding the physical principles governing chromatin folding remains a key challenge in chromatin biology^6–8^. A number of experimental techniques, including cryo-electron microscopy (cryo-EM), cryo-electron tomography (cryo-ET), X-ray scattering, super-resolution fluorescence microscopy, NMR and chromosome conformation capture methods, have been employed to explore the structural organization of chromatin both in vitro and in vivo^9–16^. While these methods have provided crucial insights into chromatin architecture, achieving atomic-level resolution remains difficult, particularly for histone tails in condensed chromatin regions. This limitation arises from the intrinsic flexibility of chromatin and the transient, heterogeneous nature of histone tail interactions with both nucleosomal and linker DNA^17–19^. Earlier structural models of chromatin posited that histone tails may mediate the inter-nucleosomal interactions predominantly through nucleosome face-to-face stacking across very short distances^9,20–22^, while recent cryo-EM studies suggest that nucleosomes may interact through their tails at larger distances and various orientations^12,23,24^. A full mechanistic understanding of how different histone tails contribute to chromatin compaction remains incomplete^25^.

Another major challenge involves reconstructing realistic 3D chromatin models from cryo-EM or ET maps. Traditionally, nucleosome organization has been inferred by rigidly fitting mono-nucleosome crystal structures into high-density volumes obtained from cryo-ET or EM data^9,11,12^. While these approaches provide valuable structural insights, they suffer from some limitations: they rely on arbitrary decisions about nucleosome chain direction, often connect nucleosomes using straight DNA linkers, do not take into account histone tail-mediated interactions and ignore the geometric and torsional constraints of DNA. These issues are exacerbated in highly condensed chromatin regions, where ambiguous fits and poor DNA resolution complicate the generation of reliable models. Recent studies have tried to address these limitations by incorporating more realistic representations of linker DNA. For example, linker DNA models have been reconstructed from cryo-ET densities using polymer-based models, such as worm-like chains^26^. In addition, fragment-based approaches combined with hierarchical chain growth have been employed to generate conformational ensembles of linker DNA at atomistic resolution^27^. Despite these advances, a consistent and physically rigorous framework that integrates linker DNA geometry, nucleosome orientation, and array-level organization remains lacking.

To overcome these limitations and provide mechanistic insight into nucleosomal array folding, we employed all-atom molecular dynamics (MD) simulations of different tri-nucleosome models with various linker lengths. Tri-nucleosomes represent the minimal chromatin unit capable of capturing inter-nucleosomal geometry, linker DNA conformations, and histone tail interactions. We demonstrated that these tri-nucleosome models allow for near-atomistic interpretation of cryo-ET density maps of native human chromatin. Namely, these models specify the relative orientation of consecutive nucleosomes, while simultaneously and inherently incorporating DNA and protein geometric and torsional constraints, even in partially resolved maps. This tri-nucleosome-based fitting framework provides a practically superior strategy for building biologically meaningful chromatin models. Additionally, we present a systematic analysis of tri-nucleosome dynamics in systems with and without histone tails and show that histone tails are critical for driving nucleosome array compaction and mediating inter-nucleosomal interactions. Importantly, we find that the tri-nucleosome conformations generated in our simulations reproduce key stereological features of experimental native chromatin. Moreover, we reveal previously uncharacterized modes of histone tail interaction that may provide mechanistic insights into higher-order chromatin structure.

## Results

### Tri-nucleosome fitting into experimental cryo-ET maps

**Fig. 1:**
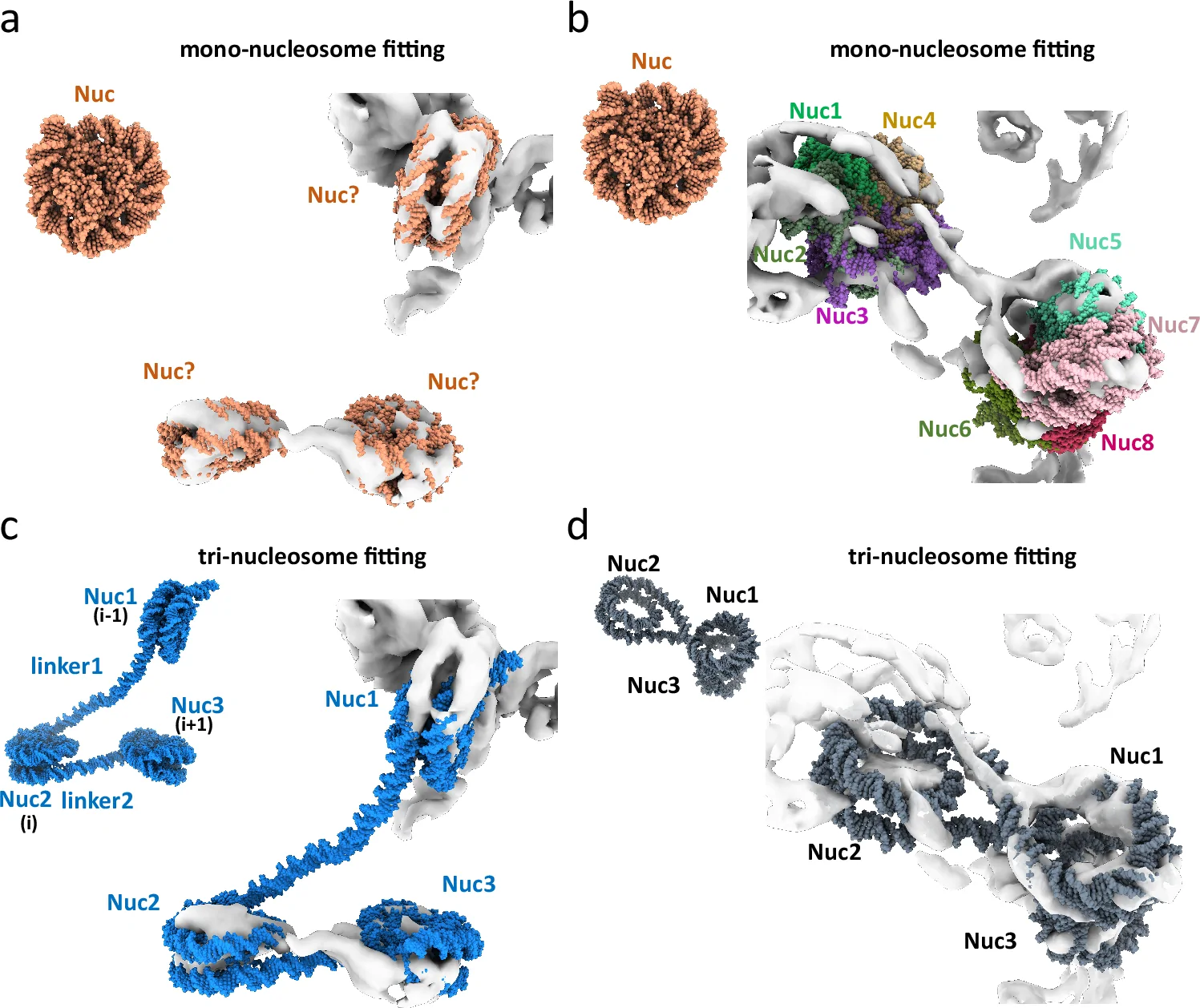
Representative fitted mono-nucleosome and tri-nucleosome structural models into cryo-ET density maps of human chromatin. Fitting of a mono-nucleosome structure with truncated tails (PDB: 1KX5) into: **a** high-density regions of the cryo-ET map of an open chromatin region (chromatin array vitrified at 0 mM Mg^2+^), **b** condensed chromatin region (chromatin array vitrified at 0.75 mM Mg^2+^). Fitting of a representative tri-nucleosome conformation from ${{{\rm{NUC}}}}_{{{\rm{notail}}}}^{22}$ MD simulations into: **c** cryo-ET map as shown in (**a**); **d** cryo-ET map as shown in (**b**).

Previous efforts to model chromatin and nucleosome arrays predominantly relied on fitting of a mono-nucleosome core particle X-ray crystal structure into the high-density regions of cryo-ET/EM maps^9,11,12,28,29^. However, mono-nucleosome-based fitting strategies have several inherent limitations. One major drawback is the arbitrary selection of the nucleosome connection path, which often leads to ambiguous or inconsistent definitions of consecutive nucleosome arrangement (Fig. 1a and see Fig. S1 for strong contrast with a black background and volume rendering of cryo-ET density maps. The corresponding XZ views of the cryo-ET density maps are shown in Fig. S2). Additionally, straight-line DNA segments are typically used to connect nucleosomes and ignore the torsional mechanics and geometric constraints of DNA, such as twist, rise, and bend, that are essential for maintaining the structural integrity and topological regulation of chromatin^23,30^. Another major limitation of mono-nucleosome fitting occurs in condensed chromatin regions within the cryo-ET map, where multiple mono-nucleosomes can be erroneously fitted into the same high electron density volume area, often in implausible mutual orientations (Fig. 1b). This necessitates manual intervention and subjective decision-making to determine reasonable nucleosome configurations based on permissible nucleosome arrangements.

Our approach utilizes various tri-nucleosome conformations derived from MD simulations for fitting to the cryo-ET maps. These conformations inherently define the relative spatial organization and order of three consecutive nucleosomes (Nuc1, Nuc2, and Nuc3), providing information about nucleosome arrangement and a more meaningful representation of local regions of chromatin and nucleosome clutches (Fig. 1c). Importantly, since these tri-nucleosome conformations are sampled from physically plausible MD trajectories, the associated linker DNA segments naturally embody torsional strain and geometric constraints, possibly offering a more accurate and/or plausible structural conformation and interpretation for modeling of nucleosome arrays. Moreover, when tri-nucleosome core structures are aligned with appropriate high-density regions in the map, the linker DNA segments connecting the three nucleosomes can be positioned in alignment with the corresponding DNA density, even when the linker DNA densities are partially missing or poorly resolved in the cryo-ET map (Fig. 1d). As such, tri-nucleosome fitting can provide insight into the geometry and connectivity of nucleosome arrays within chromatin.

Therefore, we assessed the goodness of fit of human cryo-ET maps using the MD-generated tri-nucleosome conformations. To better recapitulate native chromatin fibers with varying linker lengths, we also collected the MD trajectories from three tri-nucleosome systems (${{{\rm{NUC}}}}_{{{\rm{tail}}}}^{22}$, ${{{\rm{NUC}}}}_{{{\rm{tail}}}}^{26}$ and ${{{\rm{NUC}}}}_{{{\rm{tail}}}}^{31}$), each incorporating histone tails and linker DNA lengths of 22 bp, 26 bp, and 31 bp, respectively. These cryo-ET maps were collected under two experimental conditions: 0 mM Mg²⁺ (open chromatin fiber) and 0.75 mM Mg²⁺ (condensed chromatin fiber), the latter approximating the physiological concentration of free magnesium in a cellular nucleus^12,31^ (Figs. S3 and S4). Since cryo-ET maps mostly showed the arrangement of nucleosomes in nucleosomal arrays, we will use the terminology of “nucleosome array” or “chromatin fiber” throughout the text. Although three tri-nucleosome systems with histone tails and different linker lengths were considered, only one system, ${{{\rm{NUC}}}}_{{{\rm{tail}}}}^{22},$ was used for comparison with the identical system without tails ${{{\rm{NUC}}}}_{{{\rm{notail}}}}^{22}$_._

We found that fitting of MD conformations of the tri-nucleosome system without histone tails (${{{\rm{NUC}}}}_{{{\rm{notail}}}}^{22}$) into the nucleosome array, captured at 0 mM Mg^2+^, yielded relatively high values for goodness-of-fit measures, estimated as correlation coefficients and “inside-contour”, calculated for the whole tri-nucleosome system (Figs. S5 and S6). The fitting quality was statistically significantly higher than that obtained using systems with histone tails ${{{\rm{NUC}}}}_{{{\rm{tail}}}}^{22}$ and was comparable to that of mono-nucleosome fitting. These results suggest that the tri-nucleosomes without tail conformations closely resemble open nucleosome arrays. Conversely, we found that the ${{{\rm{NUC}}}}_{{{\rm{tail}}}}^{22}$ conformations better matched the condensed nucleosome arrays when compared to the ${{{\rm{NUC}}}}_{{{\rm{notail}}}}^{22}$ system (Figs. S5b and S6b). The goodness of fit correlation coefficients were considerably higher compared to the mono-nucleosome fitting, even though fitting with mono-nucleosomes can in general produce a more accurate local placement, where the local correlation coefficient is maximized (see “Methods”). This suggests that tri-nucleosome conformations capture structural features that extend beyond mono-nucleosome disks. Compared to the ${{{\rm{NUC}}}}_{{{\rm{tail}}}}^{22}$ system, both ${{{\rm{NUC}}}}_{{{\rm{tail}}}}^{26}$ and ${{{\rm{NUC}}}}_{{{\rm{tail}}}}^{31}$ systems yielded statistically lower goodness of fit values for condensed nucleosome arrays, whereas ${{{\rm{NUC}}}}_{{{\rm{tail}}}}^{26}$ produced a somewhat better fit for an open array (Figs. S7 and S8). It should be mentioned that the inside-contour values could not be directly compared between tri-nucleosome and mono-nucleosome systems because this measure is very sensitive to the fitted system’s size.

It has been argued previously that different denoising filters can substantially influence the quality of fitting of structural models into the cryo-ET maps. The original cryo-ET maps used in this study were previously denoised with the deep-learning non-linear filter^32^. Therefore, we tested other denoising filters: NAD filter (non-linear) and a Gaussian filter (linear)^12,33^. Comparisons of the three filtering approaches using one tomogram illustrate that there are no significant differences in either orientations or positions of the fitted models across the three methods (Fig. S9). The corresponding raw data are provided in Supplementary Data 1.

### Comparisons of structural characteristics derived from simulations and experiments

**Fig. 2:**
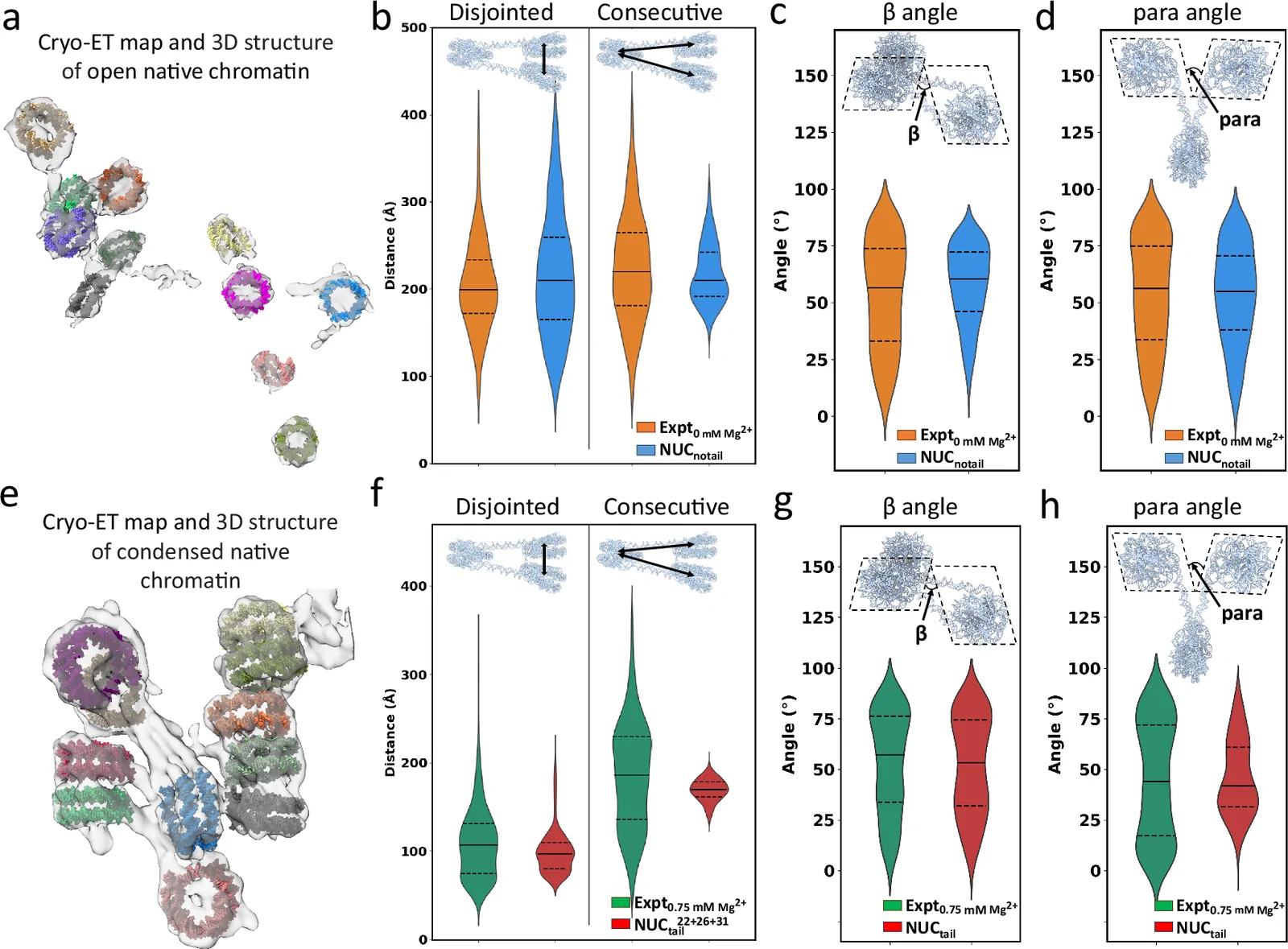
Comparisons of structural characteristics from tri-nucleosome MD simulations and experimental cryo-ET maps. To infer the experimental inter-nucleosome distance distributions from cryo-ET maps, mono-nucleosome structures (without histone tails, PDB: 1KX5) were rigid-body fitted into the cryo-ET density maps (gray) of an open chromatin fiber at 0 mM Mg²⁺ (**a**), and of a condensed chromatin fiber at 0.75 mM Mg²⁺ (**e**), as was done previously^12^. Colors assigned to the fitted mono-nucleosomes do not encode any structural or functional property and are used only for visual clarity. **b** Distributions of disjointed and consecutive nucleosome center-to-center distances inferred from experimental open chromatin fibers and from ${{{\rm{NUC}}}}_{{{\rm{notail}}}}^{22}$ simulations. **f** Distributions of disjointed and consecutive nucleosome center-to-center distances inferred from experimental condensed chromatin fiber and from the ${{{\rm{NUC}}}}_{{{\rm{tail}}}}^{22}$, ${{{\rm{NUC}}}}_{{{\rm{tail}}}}^{26}$, and ${{{\rm{NUC}}}}_{{{\rm{tail}}}}^{31}$ systems combined. Their initial models’ disjointed center-to-center distances were 65, 154, and 91 Å, and the initial consecutive center-to-center distances were 168, 163, and 167 Å, respectively. Distributions of β angles inferred from experimental open chromatin fibers and ${{{\rm{NUC}}}}_{{{\rm{notail}}}}^{22}$ simulations (**c**); from experimental condensed chromatin fiber and ${{{\rm{NUC}}}}_{{{\rm{tail}}}}^{22}$, ${{{\rm{NUC}}}}_{{{\rm{tail}}}}^{26}$, and ${{{\rm{NUC}}}}_{{{\rm{tail}}}}^{31}$ systems combined. Initial β angles were 57°, 51°, and 70°, respectively (**g**). Distributions of para-angle values inferred from experimental open chromatin fibers and ${{{\rm{NUC}}}}_{{{\rm{notail}}}}^{22}$ simulations (**d**); from experimental condensed chromatin fiber and ${{{\rm{NUC}}}}_{{{\rm{tail}}}}^{22}$, ${{{\rm{NUC}}}}_{{{\rm{tail}}}}^{26}$, and ${{{\rm{NUC}}}}_{{{\rm{tail}}}}^{31}$ systems combined. Initial para-angle values were 27°, 57°, and 50°, respectively (**h**). Data were collected from all three independent MD runs. The distributions for individual systems ${{{\rm{NUC}}}}_{{{\rm{tail}}}}^{22}$, ${{{\rm{NUC}}}}_{{{\rm{tail}}}}^{26}$, and ${{{\rm{NUC}}}}_{{{\rm{tail}}}}^{31}$ are given in Fig. S11.

To provide another level of cryo-ET map interpretation, we turned our attention to directly comparing structural characteristics derived from simulations to those obtained from the native experimental cryo-ET maps, without any tri-nucleosome fitting involved. We calculated the following structural characteristics: Euclidean distance between the disjointed nucleosomes’ centers of mass (Nuc1–Nuc3, corresponding to N-distance in ref. ^12^), Euclidean distance between the consecutive nucleosomes’ centers of mass (Nuc1–Nuc2 or Nuc2–Nuc3, corresponding to D-distance), dihedral angles formed by two consecutive nucleosomal planes (between Nuc1 and Nuc2 or between Nuc2 and Nuc3, −β angle) and dihedral angles formed by two disjointed nucleosomal planes (between Nuc1 and Nuc3, −*para* angle). The experimental data on distances and angles and their definitions were taken from the previous study (Figs. S10 and S11)^12^. Consistent with the better fitting quality, the distributions of MD-generated parameters from the ${{{\rm{NUC}}}}_{{{\rm{notail}}}}^{22}$ system were found to be in strong agreement with those of open experimental chromatin arrays at 0 mM Mg²⁺ with the median disjointed and consecutive nucleosome distances of around 200 and 218 Å, respectively (Fig. 2a–d). Both distributions of distances and dihedral angles were wide, and systems showed high heterogeneity.

In condensed nucleosome arrays, inter-nucleosomal distances derived from experiments were much smaller, especially for disjointed distances (median value of 100 Å), compared to the open fiber (without magnesium), reflecting the magnesium-induced chromatin fiber compaction (Fig. 2e, f)^12,23,34^. The results showed that the distributions of distances and dihedral angles, which covered all conformations with different linker lengths, collectively reproduced the experimental behavior of condensed native chromatin fibers (Fig. 2f–h). Namely, the median values considerably differed from those calculated from the initial tri-nucleosome conformations (after equilibration), pointing to sampling of various conformations (see Fig. 2 captions). At the same time, simulated distance and angle distributions were narrower than experimental ones, possibly pointing to some degree of conformational under-sampling. Representative and initial conformations of different tri-nucleosome systems are shown in Fig. S12. From this figure and Fig. S13, it is clear that ${{{\rm{NUC}}}}_{{{\rm{tail}}}}^{22}$ and ${{{\rm{NUC}}}}_{{{\rm{tail}}}}^{31}$ are characterized by more prominent face-to-face nucleosome disk orientations with smaller disjointed distances and angle *para* values, compared to ${{{\rm{NUC}}}}_{{{\rm{tail}}}}^{26}$ which is characterized by more open side-to-side nucleosome orientations, which explains its better fit to the open array.

### Structural and dynamic determinants of the structural models’ predictive power

**Fig. 3:**
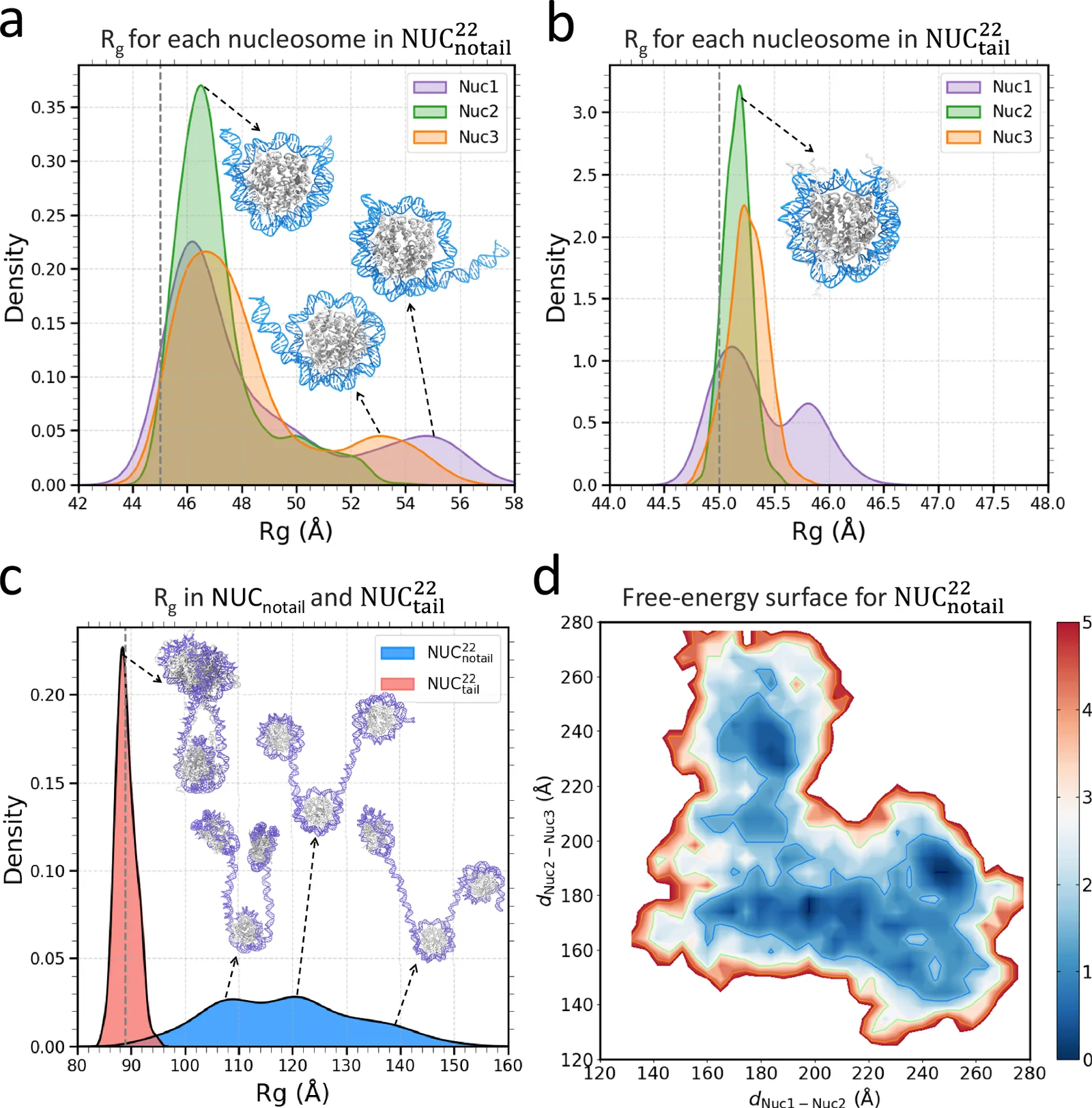
Role of histone tails in mediating tri-nucleosome compaction. **a** Radius of gyration (Rg) distributions for each nucleosome (Nuc1: purple, Nuc2: green, and Nuc3: red) in the ${{{\rm{NUC}}}}_{{{\rm{notail}}}}^{22}$ system. The grey dashed line shows the Rg values of a single nucleosome (Nuc2) extracted from the initial ${{{\rm{NUC}}}}_{{{\rm{notail}}}}^{22}$ system (after equilibration). **b** Same as (**a**), for the ${{{\rm{NUC}}}}_{{{\rm{tail}}}}^{22}$ system. **c** Distributions of Rg for all three nucleosomes in ${{{\rm{NUC}}}}_{{{\rm{notail}}}}^{22}$ (blue) and ${{{\rm{NUC}}}}_{{{\rm{tail}}}}^{22}$ (red) tri-nucleosome systems. **d** A free-energy surface as a function of center-to-center distances between nucleosome 1 (Nuc1) and nucleosome 2 (Nuc2), and between nucleosome 2 and nucleosome 3 (Nuc3) in ${{{\rm{NUC}}}}_{{{\rm{notail}}}}^{22}$ simulations. Free energies are in kcal/mol. Data were collected from all three independent MD runs.

Here, we analyzed the structural and dynamic features that might underlie the tri-nucleosome models’ fitting/predictive power by comparing ${{{\rm{NUC}}}}_{{{\rm{tail}}}}^{22}$ to ${{{\rm{NUC}}}}_{{{\rm{notail}}}}^{22}$ systems. The ${{{\rm{NUC}}}}_{{{\rm{tail}}}}^{22}$ system maintained a condensed architecture throughout the simulations, with Nuc1 and Nuc3 consistently closely apposed, which, in turn, was reflected in smaller values and a narrower radius of gyration (Rg) distribution as well as smaller inter-nucleosomal distances compared to the ${{{\rm{NUC}}}}_{{{\rm{notail}}}}^{22}$ system (Fig. 3a, b). These observations were further supported by the radius of gyration distribution of the whole system (including all three nucleosomes) (Fig. 3c): systems without tails displayed a broad distribution with three peaks that corresponded to various unfolding pathways: single-sided unfolding from Nuc1, unfolding from Nuc3, and simultaneous unfolding from both Nuc1 and Nuc3. These findings highlight the critical role of histone tails in constraining DNA unwrapping and inter-nucleosome unfolding, preventing the adoption of open conformations, corroborating earlier reports on tail-mediated chromatin condensation^22,35–37^.

The tri-nucleosome system without tails serves as a suitable model for open chromatin fiber fitting. In the ${{{\rm{NUC}}}}_{{{\rm{notail}}}}^{22}$ system, MD simulations revealed pronounced opening of linker regions and unfolding of one or both flanking nucleosomes relative to Nuc2, which was used as a reference for aligning MD trajectories/frames (Fig. S14). This process led to an increase in the effective linker lengths, enabling them to span the distances between nucleosomes in an open fiber and approximate the linker length distribution observed in native chromatin. Moreover, the linkers were found to be flexible (see MD trajectories) and deviated from the linear DNA conformations (Fig. 3c). It should be mentioned that although native chromatin contains nucleosomes with histone tails, ${{{\rm{NUC}}}}_{{{\rm{notail}}}}^{22}$ system serves as a useful reference for the tailed system to demonstrate the effect of histone tails and effective linker lengths’ contributions to chromatin compaction.

Next, we tried to interpret our findings by examining the Gibbs free energy landscape as a function of disjointed nucleosomal center-center distances (d_Nuc-Nuc_). In the ${{{\rm{NUC}}}}_{{{\rm{notail}}}}^{22}$ system, the free-energy landscape revealed three wide and dispersed energy minima, aligning with the tri-modal distribution of the radius of gyration of the whole tri-nucleosome system (Fig. 3d). Moreover, the energy distribution was wider along the d_Nuc2-Nuc1_ axis compared to the one along the d_Nuc2-Nuc3_ axis. This asymmetry suggests that Nuc1 is more susceptible to unfolding from the middle nucleosome than Nuc3. In contrast, the ${{{\rm{NUC}}}}_{{{\rm{tail}}}}^{22}$ system exhibited a much narrower free-energy basin (Fig. S15).

### Molecular mechanisms of tail–tail mediated nucleosome array compaction

Having established that the tri-nucleosome systems with histone tails may capture some aspects of the behavior of condensed chromatin, we next sought to elucidate the molecular mechanisms by which histone tails facilitate chromatin compaction at the tri-nucleosome level. For the nucleosome systems with tails, our results showed that the average numbers of inter-nucleosomal contacts were 256, 85, and 335 for ${{{\rm{NUC}}}}_{{{\rm{tail}}}}^{22}$, ${{{\rm{NUC}}}}_{{{\rm{tail}}}}^{26}$ and ${{{\rm{NUC}}}}_{{{\rm{tail}}}}^{31}$ systems, with tail–tail interactions contributing 29%, 14%, and 8% of all contacts, respectively (Figs. S16 and S17). The ${{{\rm{NUC}}}}_{{{\rm{tail}}}}^{22}$ system had the highest number of tail–tail contacts, followed by ${{{\rm{NUC}}}}_{{{\rm{tail}}}}^{31}$ system, whereas ${{{\rm{NUC}}}}_{{{\rm{tail}}}}^{26}$ had the smallest number of tail–tail contacts. In the representative conformations of the ${{{\rm{NUC}}}}_{{{\rm{tail}}}}^{26}$ system, the disjointed nucleosome planes are nearly juxtaposed to each other (Fig. S12b), making it difficult for histone tails from the disjointed nucleosomes to contact one another. In the ${{{\rm{NUC}}}}_{{{\rm{tail}}}}^{31}$ system, the inter-nucleosomal contacts between histone tails from the disjointed nucleosomes are hindered by the relatively large center-to-center nucleosome distances (Fig. S12c).

**Fig. 4:**
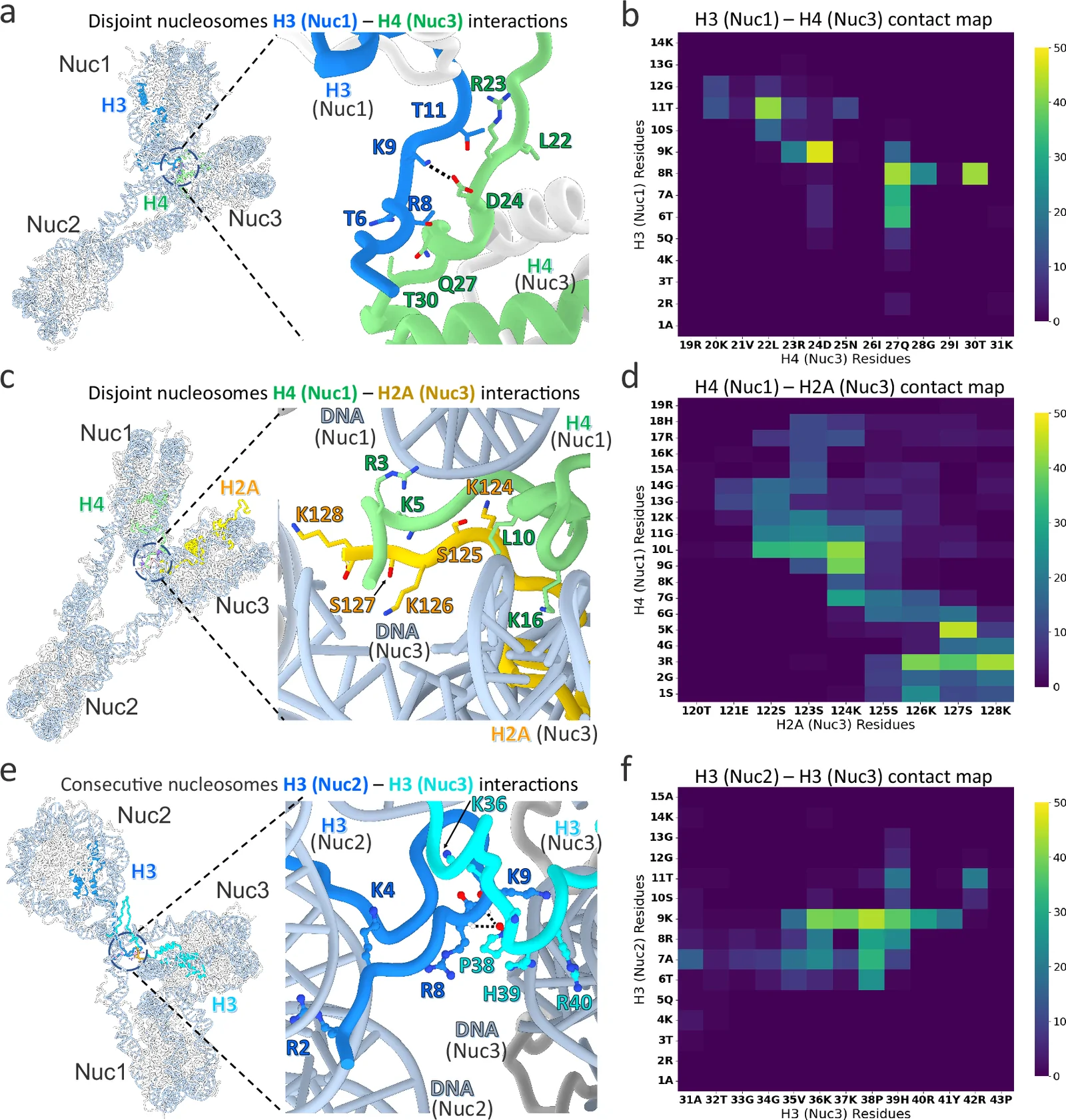
Histone tail–tail interactions in mediating tri-nucleosome compaction. **a** An illustration of the disjointed nucleosomal interactions between the H3 N-terminal tail of Nuc1 and the H4 N-terminal tail of Nuc3 in the ${{{\rm{NUC}}}}_{{{\rm{tail}}}}^{22}$ system, showing key interacting residues. **b**, **d**, **f** Contact maps show the percentage of all contacts in MD simulation frames between the corresponding tails in all three independent runs in the ${{{\rm{NUC}}}}_{{{\rm{tail}}}}^{22}$ system. Contact maps for individual independent runs are shown in Figs. S16–S18. **c** Disjointed nucleosomal interactions between the H4 N-terminal tail of Nuc1 and the H2A C-terminal tail of Nuc3 via DNA-mediated bridging in the ${{{\rm{NUC}}}}_{{{\rm{tail}}}}^{22}$ system. **e** Consecutive nucleosome interaction between the H3 N-terminal tails of Nuc2 and Nuc3 in the ${{{\rm{NUC}}}}_{{{\rm{tail}}}}^{22}$ system.

Below we present an analysis of atomistic tail–tail contacts in the$\,{{{\rm{NUC}}}}_{{{\rm{tail}}}}^{22}$ system. Among disjointed inter-nucleosomal interactions in the ${{{\rm{NUC}}}}_{{{\rm{tail}}}}^{22}$ system, two types of tail–tail contacts were particularly prevalent between Nuc1 and Nuc3 (Fig. 4a). First, the salt bridge formation was observed between positively charged residue K9 of the H3 tail and negatively charged residue D24 of the H4 tail (Fig. 4a, b, and see Fig. S18 for contact maps of different simulation runs). The second type of disjointed inter-nucleosomal interaction was observed between the two DNA gyres of neighboring nucleosomes mediated by histone tails. Specifically, the positively charged residues R3 and K16 from the H4 tail and K124, K126, and K128 from the H2A tail were inserted into the DNA grooves of Nuc1 and Nuc3, respectively, and partially neutralized the negative charge of DNA, so that DNA gyres of neighboring nucleosomes could come close to each other in space (Fig. 4c, d, and Fig. S19). It should be noted that most of the observed H4-H2A tail–tail contacts arise from interactions between the H4 N-terminal tail and the H2A C-terminal tail. We did not observe a significant number of interactions between the H4 tail and the H2A acidic patch residues in the ${{{\rm{NUC}}}}_{{{\rm{tail}}}}^{22}$ system.

We also identified the third distinct type, involving consecutive nucleosomal interactions, mediated by their H3 tails, that neutralized the DNA charge of neighboring nucleosomes and brought them closer together in space (Fig. 4e). For instance, the H3 tail of Nuc2 interacted with the linker DNA and the nucleosomal DNA of Nuc3. Key residues included H3 R2, K4, and K9, which were inserted into the minor grooves of the linker and nucleosomal DNA (Fig. 4e). Notably, hydrogen bonding was observed between P38 of the H3 tail from Nuc3 and the backbone of R8 and K9 from the H3 tail of Nuc2 (Fig. 4f and Fig. S20). As can be seen, these tail–tail interactions were predominantly mediated by lysine residues, whereas arginines were more stably inserted into the DNA minor grooves, as observed in our previous simulations^38,39^.

### Molecular mechanisms of tail–DNA mediated nucleosome array compaction

**Fig. 5:**
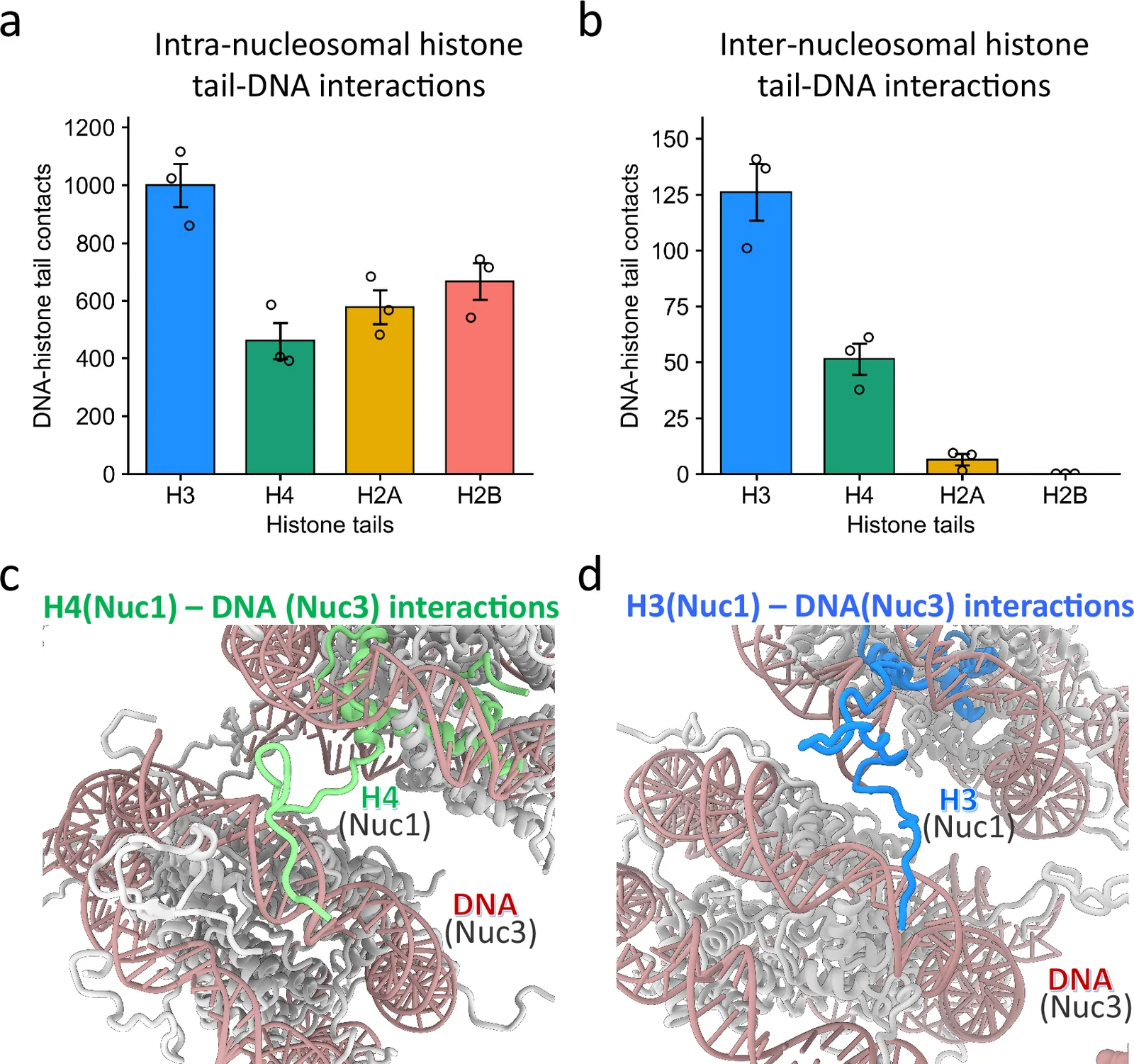
Histone tail–DNA interactions in mediating tri-nucleosome compaction. **a** The average number of intra-nucleosomal histone tail–DNA contacts (histone tails interacting with the DNA from their originating nucleosome) per frame in the ${{{\rm{NUC}}}}_{{{\rm{tail}}}}^{22}$ system. Data are presented as mean values ± SEM from three independent simulation runs (*n* = 3). **b** The average number of inter-nucleosomal histone tail–DNA contacts between Nuc1-DNA (Nuc2 or Nuc3), Nuc2-DNA (Nuc1 or Nuc3) and Nuc3-DNA (Nuc1 or Nuc2) per frame in the ${{{\rm{NUC}}}}_{{{\rm{tail}}}}^{22}$ system. **c** A representative conformation of the H4 N-terminal tail from Nuc1 inserting into the minor groove of DNA (SHL + 6) from Nuc3 that mediates the disjointed nucleosomal interactions. **d** A representative conformation of the H3 N-terminal tail from Nuc1 interacting with DNA at SHL + 1 in Nuc3 that mediates the disjointed nucleosomal interactions. The H3 and H4 tails are highlighted in blue and green, respectively, and the DNA is shown in orange. Histone tail–DNA interactions for other tri-nucleosome systems, ${{{\rm{NUC}}}}_{{{\rm{tail}}}}^{26}$ and ${{{\rm{NUC}}}}_{{{\rm{tail}}}}^{31},$ are shown in Fig. S15.

In addition to mediating inter-nucleosomal interactions through tail–tail contacts, histone tails are known to participate in interactions with DNA, which in turn may promote inter-nucleosomal interactions or restrict DNA breathing motions^25,39–44^. To characterize the binding patterns of intra- and inter-nucleosomal histone tail–DNA interactions (Fig. 5a, b), we mapped all histone tail–DNA contacts across the entire 485 bp DNA region of the tri-nucleosome system. Each nucleosome (Nuc1, Nuc2, and Nuc3) in the tri-nucleosome system was assigned its own DNA superhelical location (SHL) positions, including linker DNA, ranging from −SHL8 to +SHL8.

First, we analyzed the intra-nucleosomal tail–DNA interaction patterns and found that the number of contacts was qualitatively similar across the three nucleosome systems ${{{\rm{NUC}}}}_{{{\rm{tail}}}}^{22}$, ${{{\rm{NUC}}}}_{{{\rm{tail}}}}^{26}$, and ${{{\rm{NUC}}}}_{{{\rm{tail}}}}^{31}$ (Figs. S21–S23). Overall, with a few exceptions, these patterns closely mirrored those found in our earlier mono-nucleosome simulations, which involved a more extensive tail conformational sampling^25^. Indeed, as was shown earlier, intrinsically disordered histone tails can bind and unbind to the specific nucleosomal DNA regions and exist in a dynamic equilibrium, often referred to as fuzzy interactions with DNA^1,25,38,45,46^. Because of the nucleosome’s twofold pseudo-symmetry, good enough conformational sampling is expected to yield similar DNA region coverage for each histone copy (negative and positive SHL regions), which we indeed observed for major peaks for intra-nucleosomal interaction in the current study. Notably, different histone tails exhibited distinct preferences for different DNA regions. The H3 tails, being the longest, engaged multiple DNA regions, including sites near the dyad, and extended up to the next consequent nucleosome via contacts with the linker DNA.

As to the inter-nucleosomal tail–DNA contacts, they were responsible for the majority of all inter-nucleosomal interactions, with an average number of 182, 73, and 308 contacts in the ${{{\rm{NUC}}}}_{{{\rm{tail}}}}^{22}$, ${{{\rm{NUC}}}}_{{{\rm{tail}}}}^{26}$ and ${{{\rm{NUC}}}}_{{{\rm{tail}}}}^{31}$ systems, respectively (Figs. S17 and S21). In all systems, they were predominantly mediated by the H3 and H4 tails, while the H2A and H2B tails contributed minimally. Representative conformations are shown in Fig. 5c, d. These tail-mediated dual DNA interactions (a single tail interacting with two DNA regions from different nucleosomes) can promote close nucleosome contacts. There was also a noticeable difference between systems with different linker lengths (Fig. S17). Notably, the ${{{\rm{NUC}}}}_{{{\rm{tail}}}}^{31}$ system formed the lowest number of tail–DNA intra-nucleosomal contacts. However, a large fraction of them (up to 30% of H3 tail–DNA contacts) were released upon inter-nucleosomal interactions. As a result, ${{{\rm{NUC}}}}_{{{\rm{tail}}}}^{31}$ exhibited the highest number of inter-nucleosomal tail–DNA contacts, followed by the ${{{\rm{NUC}}}}_{{{\rm{tail}}}}^{22}$ and ${{{\rm{NUC}}}}_{{{\rm{tail}}}}^{26}$ systems, with the latter displaying approximately four-fold fewer tail–DNA inter-nucleosomal contacts compared to ${{{\rm{NUC}}}}_{{{\rm{tail}}}}^{31}$ (Fig. S17). In addition, we calculated the binding free energies of inter-nucleosomal tail–DNA interactions for ${{{\rm{NUC}}}}_{{{\rm{tail}}}}^{22}$ using the MM/GBSA method. Even though the MM/GBSA method is known to overestimate the absolute values of binding energy, the overall trend agreed with the contact analyses. It was clear that the H3 tail stood out as the main contributor to inter-nucleosomal tail–DNA binding (Supplementary Table 1).

## Discussion

Recent super-resolution microscopy studies showed that nucleosomes are often arranged in discrete and irregular clusters (“clutches”) containing anywhere from a few to a couple of dozen nucleosomes in mammalian cells^47–49^. In our study, using all-atom MD simulations of tri-nucleosomes with different linker lengths representing structural units of nucleosome clutches, we provide an integrative structural framework that connects atomistic simulations with cryo-ET map-based chromatin reconstructions. Our results establish atomistic tri-nucleosome fitting as a paradigm for understanding native chromatin plasticity at the level of nucleosome clutches. Traditional approaches for cryo-ET/EM map interpretation typically involve fitting individual mono-nucleosome structures into local high-density regions. However, this strategy often results in nucleosomes that remain disconnected or artificially linked by straight DNA linkers, disregarding the geometric and torsional constraints of DNA. These limitations become even more pronounced in highly condensed chromatin regions. Moreover, in such cases, the atomistic-level modeling is limited because histone tail conformations are typically not resolved in mono-nucleosome PDB structures and are therefore omitted in conventional modeling approaches.

Our study demonstrates that fitting tri-nucleosome conformations, sampled from physically realistic MD trajectories, into cryo-ET maps in some cases may resolve these limitations. The MD conformations not only maintain the relative positioning and orientation of three consecutive nucleosomes but also incorporate histone tails and linker DNA that reflect the intrinsic torsional mechanics and geometric constraints of DNA and proteins in chromatin^50–52^. We show that tri-nucleosome-based modeling may improve the fitting accuracy and provide atomistic structural interpretation of nucleosomal arrays. The proposed approach also reduces manual intervention during the model construction by embedding disjointed nucleosomal relationships and linker DNA geometry directly into the fitting template, especially valuable for regions where the linker DNA density is missing or partially resolved. We provide a library of successfully fitted conformations that can be used in the future by other researchers. Despite these advantages, the accuracy of the resulting models is constrained by the quality and completeness of the cryo-ET density maps. In particular, map denoising, routinely used by experimentalists, cannot recover information that is missing because of the limited tomographic tilt range. The resulting missing wedge may cause anisotropic resolution and elongation artifacts and increase uncertainty in density regions that are unfavorably oriented relative to the tilt axis. Moreover, no denoising method can fully distinguish weak biological density from noise.

Beyond structural fitting, our tri-nucleosome simulations reveal distinct structural and dynamic features of nucleosomal arrays. Histone tails have long been recognized as essential contributors to higher-order chromatin folding, self-association and compaction. It has been shown that even high concentrations of divalent cations, like magnesium, are unable to induce self-association of nucleosomal arrays without H3 and H4 histone tails^53,54^. Indeed, the intrinsically disordered nature of histone tails and their enrichment in positively charged residues enable them to partially neutralize the negative charge of DNA, thereby facilitating regular chromatin fiber compaction and inter-nucleosomal interactions^23,54–57^. Our simulations reveal that the presence of histone tails is associated with the compaction of tri-nucleosomes, as evidenced by low inter-nucleosomal distances, a narrow radius of gyration distribution and the conformational free-energy landscapes with narrow energy wells. All results have been confirmed in different independent runs. To complement previous studies, we provide an atomistic analysis of tail-mediated compaction of nucleosome arrays, which are mostly mediated by tail–DNA interactions but also involve tail–tail interactions. It has been observed that most tail–tail interactions involve lysines from H2A-H4 and H3-H4 tail pairs, forming salt bridges and hydrogen bonds, maintained in some cases on a microsecond time scale.

Previous studies have shown that the H3 tail predominantly stabilizes inter-nucleosomal interactions^58–60^ and the absence of histone H3 tails leads to more extended chromatin structures^22,36,40,61,62^. Our data show that H3 tails can span the linker DNA regions to form extensive networks of tail–tail and tail–DNA interactions with consecutive nucleosomes. Interestingly, important post-translational modification sites, like H3K9, have been found to be involved in a substantial fraction of these interactions, and, as was shown previously, its methylation could increase the interaction with neighboring nucleosomes and facilitate chromatin compaction^63^. Our results also indicate that H2A and H2B tails might play a relatively minor role in compacting the nucleosome arrays. However, it should be mentioned that these results do not necessarily apply to histone variant nucleosomes. For example, H2A.Z C-terminal tails may extend to the neighboring nucleosomes, form interactions with their acidic patch and mediate higher-order compaction^64,65^.

Compared to the ${{{\rm{NUC}}}}_{{{\rm{tail}}}}^{22}$ system, the reference ${{{\rm{NUC}}}}_{{{\rm{notail}}}}^{22}$ system with lacking histone tails has extensively sampled tri-nucleosome conformations, characterized by DNA breathing, increased disjointed and consecutive inter-nucleosomal distances, and larger effective linker DNA lengths, consistent with an open array architecture. All these observations confirm previous works^42,66–68^ and extend them by showing that tail–tail and tail–DNA interactions may mediate inter-nucleosome interactions, their spacing and dynamics. Notably, the tail-mediated inter-nucleosomal interactions are transient and allow for local dynamical behavior even in the condensed state. We should acknowledge that sampling of disordered histone tails in such large systems remains limited due to the computational cost of all-atom simulations. In addition, limitations in force-field accuracy and the omission of chromatin-associated factors, such as linker histones and other binding proteins, constrain the representativeness of the sampled histone tail conformations in the native chromatin. As a complementary approach, coarse-grained (CG) methods can offer improved sampling efficiency and may potentially capture linker DNA flexibility, large-scale chromatin heterogeneity and histone tail interactions, even though without high atomistic resolution^65,69–72^.

Several cryo-ET studies of native chromatin vitrified in vitro^12^ and in situ^23^ showed a multiplex heterogeneous nucleosome arrangement with nucleosome disks contacting each other in face-to-face, face-to-side, and side-to-side orientations. We show that there is a noticeable difference between the structure and dynamics of tri-nucleosome systems with different linker lengths. Namely, ${{{\rm{NUC}}}}_{{{\rm{tail}}}}^{22}$ and ${{{\rm{NUC}}}}_{{{\rm{tail}}}}^{31}$ systems are characterized by more prominent face-to-face nucleosome orientations mediated by extensive tail–DNA and tail–tail interactions, whereas ${{{\rm{NUC}}}}_{{{\rm{tail}}}}^{26}$ system has a more open conformation with the predominant side-to-side nucleosome orientations. Overall, the angle and distance distributions for simulated systems were found to be in good agreement with the recent study reporting distinctive folding of reconstituted nucleosome arrays with 25 bp and 30 bp linkers at low salt concentration and dilute phase^23^.

Our all-atom MD simulations reveal a mechanism for interaction between two disjointed nucleosomes (Fig. 4). Previous in situ nucleosome interaction capture experiments in human interphase cells suggested that disjointed nucleosomes are mostly engaged in *i* ± 2 interactions consistent with the two-start zigzag folding of the nucleosome array^73–76^. It has been suggested that the two-start zigzag folding is associated with gene repression, consistent with its role in directing heterochromatin-specific interactions with DNA-binding pioneer proteins^77^ and has also been observed by in situ cryo-ET at the nuclear periphery^27,28^.

In conclusion, our study introduces a strategy for interpreting the cryo-ET maps and reconstructing nucleosome array structure at atomistic resolution with inherently realistic DNA torsional constraints and inter-nucleosomal relationships. This approach may help to resolve ambiguities in cryo-ET maps, particularly in condensed chromatin regions, and ultimately capture biologically meaningful nucleosome connectivity.

## Methods

### Tri-nucleosome model construction

The tri-nucleosome models used in this study consist of three nucleosome core particles (called “nucleosomes” hereafter) connected by two linker DNA segments (Fig. S24). The *Homo sapiens* cryo-EM structure of the tri-nucleosome with a 22 bp linker (PDB ID: 6L4A) was used as the template for constructing the simulation models described below^78^. The original DNA sequence from the cryo-EM structure was replaced with a native DNA sequence generated using w3DNA^79^. This native DNA sequence was taken from the *Homo sapiens TP53* gene (GenBank ID: EU877045.1, range: 2486–2970) and spans 485 base pairs, with a 147-bp DNA segment wrapping around each nucleosome and two 22-bp DNA linkers connecting nucleosomes. Based on this cryo-EM structure, we first constructed two tri-nucleosome models containing 22 bp linker DNA: one with histone tails (${{{\rm{NUC}}}}_{{{\rm{tail}}}}^{22}$) and one without histone tails (${{{\rm{NUC}}}}_{{{\rm{notail}}}}^{22}$). In addition, we made structural models for tri-nucleosomes containing 26 bp and 31 bp linker DNA $\left(\right.{{{\rm{NUC}}}}_{{{\rm{tail}}}}^{26}$ and ${{{\rm{NUC}}}}_{{{\rm{tail}}}}^{31}\left)\right.$, since no PDB structures for such tri-nucleosomes were available. These linker lengths were chosen based on the previous study, which showed through coarse-grained simulations that tri-nucleosome folding depended on the linker length, with 26 bp and 31 bp linkers occupying distinct folding conformational clusters^59^. Linker DNA was constructed using the default B-DNA fiber model parameters from w3DNA (twist: 36.0° and rise: 3.375 Å). Namely, we inserted a 4 bp or 9 bp polyA sequence at the midpoint of the two 22 bp linker DNA segments from ${{{\rm{NUC}}}}_{{{\rm{tail}}}}^{22},$ to form 26 bp and 31 bp linker DNA segments for ${{{\rm{NUC}}}}_{{{\rm{tail}}}}^{26}$ and ${{{\rm{NUC}}}}_{{{\rm{tail}}}}^{31}$ models, respectively. DNA sequences and initial structures for each model are provided in Figs. S24 and S25. For all tri-nucleosome models, the DNA sequences of nucleosomes were kept identical. The initial configurations of histone tails (except for H3 tails) were taken from the high-resolution X-ray structure of the nucleosome core particle (PDB ID: 1KX5)^80^. The H3 histone tails were linearly extended symmetrically (with respect to the dyad axis) into the solvent to prevent clashes between nucleosomes, otherwise observed if three 1KX5 nucleosome structures were connected to form a tri-nucleosome. In this study, histone tail regions are defined as follows: H3 N-terminal tail (residues 1–43), H4 N-terminal tail (residues 1–23), H2A N-terminal tail (residues 1–15) and C-terminal tail (residues 119–124), and H2B N-terminal tail (residues 1–29).

### Molecular dynamics simulation protocol

All MD simulations were performed with the package GROMACS version 2022.3^81^, using the AMBER 14SB force field for protein parameters and OL15 for nucleic acids^82,83^, along with the Optimal Point Charge (OPC) water model. This water model has been shown to accurately reproduce bulk water properties and improve the accuracy of simulations involving nucleic acids and intrinsically disordered proteins^84^. In each simulation, the initial tri-nucleosome model was solvated in a box with a minimum distance of 20 Å between the nucleosome atoms and the box edges. Sodium chloride (NaCl) was added to the system to achieve a concentration of 150 mM.

Energy minimization was performed using the steepest descent algorithm for 10,000 steps. The system was then gradually heated to 300 K over 800 ps, with restraints applied to the atoms, followed by 1 ns of equilibration (we will refer to these models as “initial models” throughout the text). Production simulations were conducted in the isobaric-isothermal (NPT) ensemble for up to 4 µs. Temperature was maintained at 300 K using the Bussi thermostat, and pressure was controlled at 1 atm using the Parrinello-Rahman barostat. A cutoff of 10 Å was applied to short-range non-bonded vdW interactions. The Particle Mesh Ewald (PME) method with a cut-off of 10 Å was used to calculate all long-range electrostatic interactions. Periodic boundary conditions were applied, and covalent bonds involving hydrogen atoms were constrained using the LINCS algorithm^85^, allowing for a 2 fs integration time step. The coordinates of each solute were recorded every 100 ps, and a total of 40,000 frames in each simulation run were collected for analysis. Three independent simulation runs were performed for each system, 4 µs each. A summary of the simulation setup and details is provided in Supplementary Tables 2 and 3.

### Cryo-electron tomographic maps

Cryo-ET maps of chromatin from human K562 cells under two experimental conditions (0 mM Mg²⁺ and 0.75 mM Mg²⁺) were obtained from previous work^12^. To aid 3D visualization and to improve the resolution of individual nucleosome orientations, we processed the tomograms using a deep learning-based denoising method^32^. The chromatin samples, vitrification conditions, and resulting cryotomograms are listed in the Supplementary Table 1 of ref. ^12^ and the raw tilt series are available from the Dryad data repository (https://datadryad.orghttps://doi.org/10.5061/dryad.ttdz08m21). Samples analyzed in this work correspond to the tilt series TS21_1 (0 mM MgCl_2_) and TS22_1 (0.75 mM MgCl_2_).

### Fitting of MD simulation conformations to experimental cryo-ET maps

To construct three-dimensional (3D) chromatin models at near-atomic resolution, we fitted the atomic coordinates of the mono-nucleosome structure (PDB: 1KX5) or tri-nucleosome models into each denoised cryo-ET density map (7 maps for chromatin array vitrified at 0 mM Mg^2+^, 13 maps vitrified at 0.75 mM Mg^2+^) using the *fitmap* tool in ChimeraX 1.7, which performs rigid-body local optimization^86^. The voxel size was set to 8.4 Å for the 0 mM Mg²⁺ map (EM magnification 42,000) and 5.51 Å for the 0.75 mM Mg²⁺ map (EM magnification 64,000). The parameter settings in the *fitmap* tool were the same as in the previous study^12^. Specifically, the *fitmap* tool was configured with a resolution of 16 Å, a cluster angle of 200°, and a cluster shift of 24 Å. Initial random placements of the structural model into the 3D density map were performed, and then the optimal orientation and position were searched and saved that maximized the cross-correlation between the model’s density and the experimental map.

In detail, the atomistic model was converted into a density map by representing every atom by a scattering potential approximated by a Gaussian distribution. A global search comprising 1000 random initial placements was performed to explore the full map volume, followed by local rigid-body optimization to maximize the map-map correlation coefficient. Distinct fitting conformations were identified by clustering the resulting fits using angular and translational thresholds of 200° and 24 Å, respectively. Based on this protocol, we performed the fitting of tri-nucleosome conformations from the MD simulations to native chromatin cryo-ET maps. Due to the high computational cost of the fitting procedure (a large number of conformations, 40,000 frames per simulation × 3 runs × 4 tri-nucleosome systems), we selected 200 MD frames per run uniformly spaced at 20 ns intervals of tri-nucleosome system. This resulted in a total of 600 (MD frames) × 4 (systems) × 20 (cryo-ET maps) = 48,000 fitting operations, where each fitting operation corresponds to a single execution of the fitmap command on one conformation structure from the MD simulation and one cryo-ET map. The MD conformation with the highest goodness of fit correlation coefficient value was retained. The correlation coefficient shows the similarity between the experimental cryo-ET density map and the density map calculated from the atomistic model during the rigid-body fitting and is defined as:

1 $${{\rm{correlation\; coefficent}}}=\frac{\left\langle u,{v}\right\rangle }{\left|u\right|\left|v\right|}$$

Here, *u* represents the density values of the fit map generated from the atomistic nucleosome model. *v* represents the density values of the reference experimental cryo-ET map. Additionally, the “inside-contour” values were calculated, which represent the proportion of atoms from the model that lie within the experimental cryo-ET map’s contour surface. To compare to the global measures of nucleosome array compaction (disjointed and consecutive inter-nucleosome distances) from experiments, distances between the centers of nucleosomal disks were calculated after fitting the cryo-ET maps with the mono-nucleosome structural models, as was done in the previous study^12^.

### Analysis of MD simulations

For each MD simulation system, the initial 200 ns of each simulation trajectory were discarded as equilibration. The resulting trajectories were visualized using VMD 1.9.3 and analyzed with a set of VMD and GROMACS utilities. Histone-histone and histone-DNA contacts were defined as interactions between any two non-hydrogen atoms within 4.0 Å of each other. The radius of gyration of the DNA segments was calculated using the GROMACS tool gmx_gyrate^81^. The multi-dimensional free energy landscapes were computed using another GROMACS tool, gmx_sham, which estimates the Gibbs free energy by Boltzmann inversion of frequency histograms. The nucleosome center-center distances were used as collective variables. The interaction energies between the inter-nucleosomal histone tail and DNA were determined using the Molecular Mechanics Generalized Born Surface Area (MM/GBSA) method^87^. The salt concentration was set to 0.15 M, and the generalized Born model OBC2 was used with parameters igb = 5 and radii = mbondi2. The fast LCPO algorithm was applied to compute an analytical approximation to the solvent accessible area of the molecule (molsurf = 0). The calculations were performed based on every 1 ns frame.

### Statistics and reproducibility

Three independent MD simulation replicates were performed for each of the four tri-nucleosome systems: ${{{\rm{NUC}}}}_{{{\rm{notail}}}}^{22}$, ${{{\rm{NUC}}}}_{{{\rm{tail}}}}^{22}$, ${{{\rm{NUC}}}}_{{{\rm{tail}}}}^{26}$ and ${{{\rm{NUC}}}}_{{{\rm{tail}}}}^{31}$. For time-dependent quantities, values were first averaged over time within each trajectory and then averaged across the three independent replicates. Error bars represent the standard error of the mean (SEM) across replicates. Reproducibility was assessed by applying identical simulation protocols and analysis procedures to all independent trajectories. Statistical comparisons were performed using one-way ANOVA followed by Tukey’s post hoc test for normally distributed data and the Kruskal–Wallis test followed by pairwise Mann–Whitney *U* tests for non-normally distributed data.

### Reporting summary

Further information on research design is available in the Nature Portfolio Reporting Summary linked to this article.

## Supplementary information

Supplementary Information

Description of Additional Supplementary Files

Supplementary Data 1

Reporting Summary

Transparent Peer Review File

## Source data

Source Data

## Data Availability

The molecular dynamics simulation trajectory data generated in this study have been deposited in the Figshare repository at https://doi.org/10.6084/m9.figshare.32145079 and https://doi.org/10.6084/m9.figshare.32372565. Nucleosome structures used in this study include PDB accession codes 6L4A and 1KX5. The chromatin samples and vitrification conditions are deposited in the Figshare repository at https://doi.org/10.6084/m9.figshare.33246018. The chromatin raw tilt series are available from the Dryad data repository (https://datadryad.org/dataset/doi:10.5061/dryad.ttdz08m21). The library of fitted conformations generated in this study has been deposited in the Figshare repository at https://doi.org/10.6084/m9.figshare.32399967. Source data are provided with this paper.

## Code availability

The scripts in this study are available from the Figshare repository https://doi.org/10.6084/m9.figshare.33271119.
