# Supplementary material for: Molecular dynamics guided all-atom reconstruction of cryo-ET maps reveals mechanisms of histone tail mediated chromatin compaction
Source: Nat Commun. 2026 Sep 15;17:10811. doi: 10.1038/s41467-026-77871-9 (PMC13669341; doi:10.1038/s41467-026-77871-9)
Supplement: Supplementary file 1 [file 41467_2026_77871_MOESM1_ESM.pdf]

Supplementary information

Molecular Dynamics Guided All-Atom Reconstruction of Cryo-ET Maps Reveals Mechanisms of Histone Tail Mediated Chromatin Compaction

Shuxiang Li[1], Maria J. Aristizabal[2,3], Sergei A. Grigoryev[4], Anna R. Panchenko[1,3,5,6]*

[1] Department of Pathology and Molecular Medicine, Queen's University, ON, Canada

[2] Biology Department, Queen's University, ON, Canada

[3] Department of Biomedical and Molecular Sciences, Queen's University, ON, Canada

[4] Department of Molecular and Precision Medicine, College of Medicine, Penn State University, PA, USA

[5] School of Computing, Queen's University, ON, Canada

[6] Department of Physics, Engineering Physics & Astronomy, Queen's University, ON, Canada

*- corresponding author, Tel: +1 613 533 6000; Email: anna.panchenko@queensu.ca

Supplementary Table 1. Binding free energy of inter-nucleosomal histone tail-DNA interactions in the $\mathrm{NUC}_{\mathrm{tail}}^{22}$ system calculated using MM/GBSA approach. Free energy calculations exclude interactions between histone tails and the DNA of their originating nucleosome. For example, the inter-nucleosomal H3 tail-DNA binding free energy includes H3 tail (Nuc1) with DNA (Nuc2 and Nuc3), H3 tail (Nuc2) with DNA (Nuc1 and Nuc3) and H3 tail (Nuc3) with DNA (Nuc1 and Nuc2). Similarly, the inter-nucleosomal H4 tail-DNA binding free energy includes H4 tail (Nuc1) with DNA (Nuc2 and Nuc3), H4 tail (Nuc2) with DNA (Nuc1 and Nuc3) and H4 tail (Nuc3) with DNA (Nuc1 and Nuc2), and the same definition applies to H2A and H2B tails. Standard errors were calculated from three independent simulation runs.

Histone tail type	ΔG (kcal/mol)
**H3 tail**	-112.2 ± 16.3
**H4 tail**	-60.2 ± 5.9
**H2A tails (N-ter and C-ter)**	-16.7 ± 4.1
**H2B tail**	-0.6 ± 0.1

Supplementary Table 2. Summary of the simulation setup.

Simulation systems	Force field	Water model	Simulation replicas	Simulation time per run
$\mathbf{NUC}^{\mathbf{22}}_{\mathbf{notail}}$	AMBER FF14SB + OL15	OPC	3 runs	4000 ns
$\mathbf{NUC}^{\mathbf{22}}_{\mathbf{tail}}$	AMBER FF14SB + OL15	OPC	3 runs	4000 ns
$\mathbf{NUC}^{\mathbf{26}}_{\mathbf{tail}}$	AMBER FF14SB + OL15	OPC	3 runs	4000 ns
$\mathbf{NUC}^{\mathbf{31}}_{\mathbf{tail}}$	AMBER FF14SB + OL15	OPC	3 runs	4000 ns

Supplementary Table 3. Summary of the simulation system details.

**Simulation system**	**Box dimensions (Å)**	**Total number of atoms**	**Total number of water molecules**	**Salt type**	**Salt concentration**
$\mathbf{NUC^{22}_{notail}}$	298.5 × 298.5 × 298.5	3,478,406	851,581	Sodium chloride (NaCl)	150 mM
$\mathbf{NUC^{22}_{tail}}$	302.7 × 302.7 × 302.7	3,639,214	888,921	Sodium chloride (NaCl)	150 mM
$\mathbf{NUC^{26}_{tail}}$	332.6 × 332.6 × 332.6	4,831,544	1,186,463	Sodium chloride (NaCl)	150 mM
$\mathbf{NUC^{31}_{tail}}$	324.2 × 324.2 × 324.2	4,470,065	1,096,053	Sodium chloride (NaCl)	150 mM

Figure S1. Fitting of mono-nucleosome (a and b) and tri-nucleosome models (c and d) into Cryo-ET density maps.

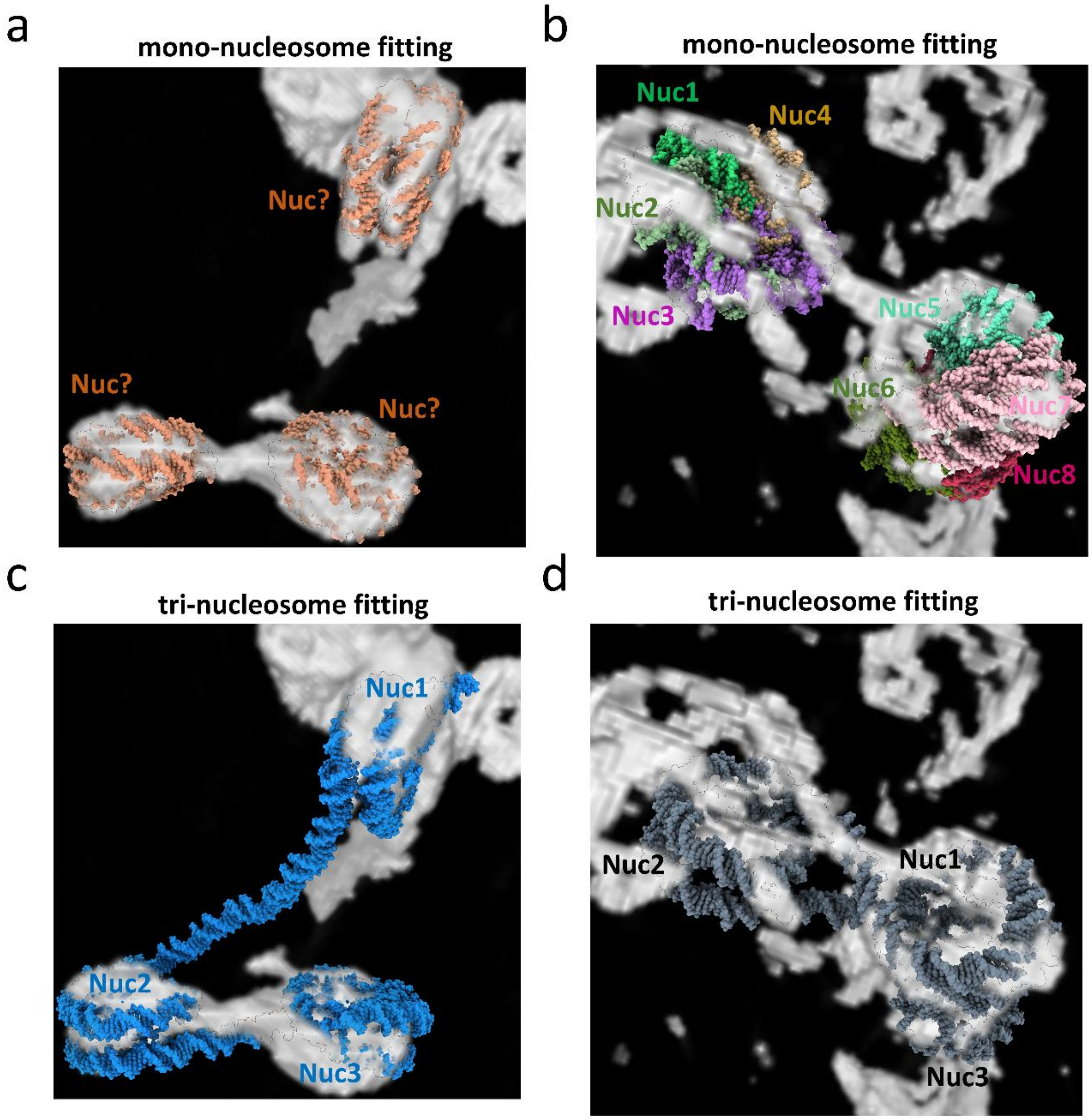

Figure S2. The XZ views of fitting of mono-nucleosome (a and b) and tri-nucleosome models (c and d) into Cryo-ET density maps.

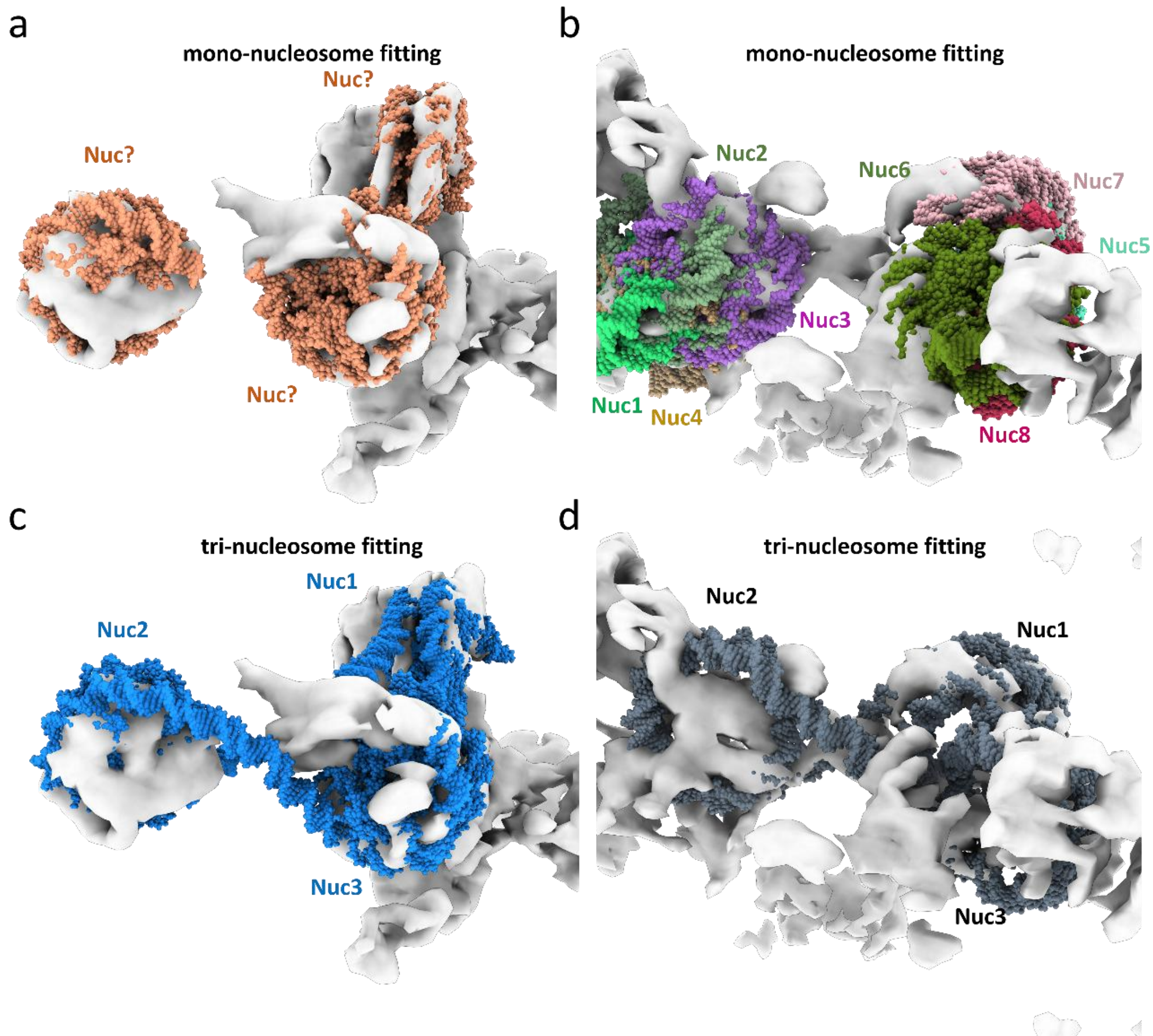

Figure S3. Denoised Cryo-ET maps of K562 native chromatin array vitrified at 0 mM Mg^{2+} from[1].

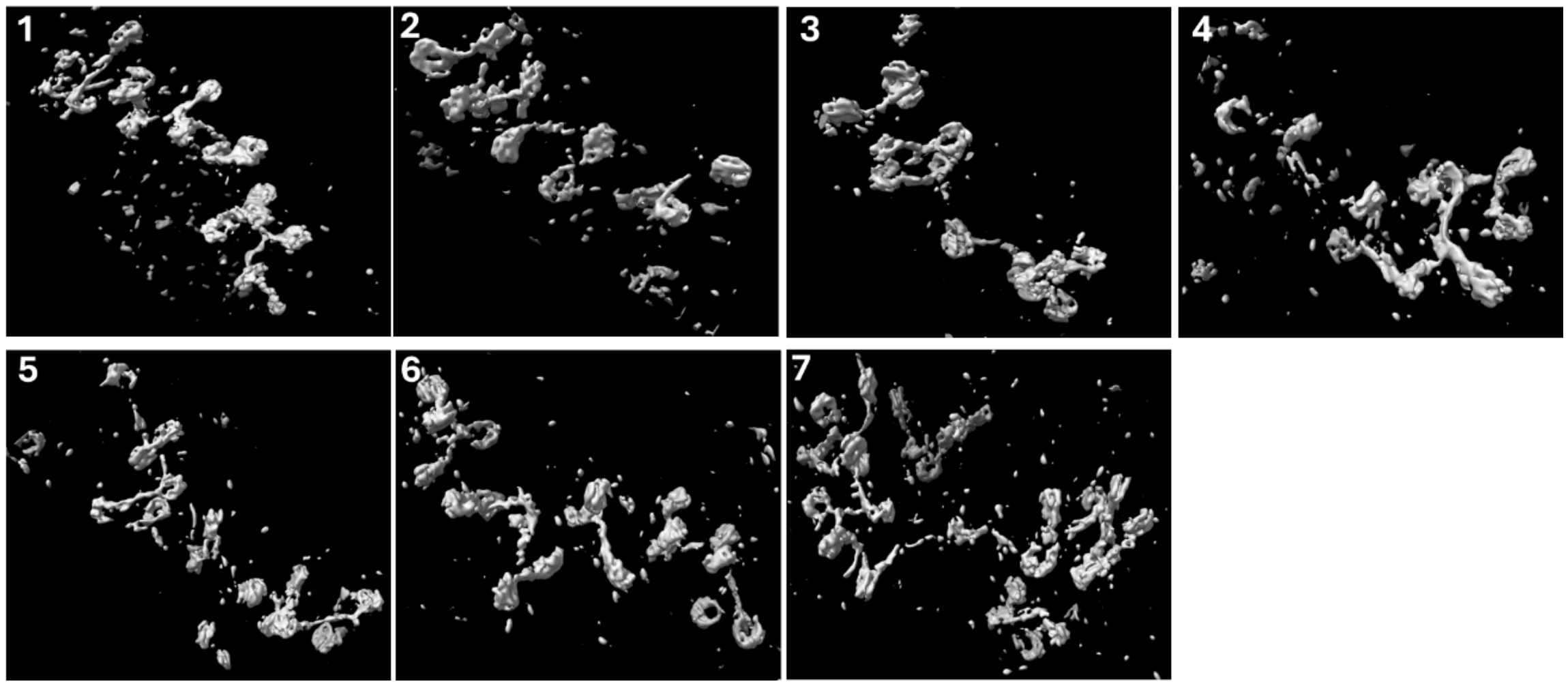

Figure S4. Denoised Cryo-ET maps of K562 native chromatin array vitrified at 0.75 mM Mg^{2+} from[1].

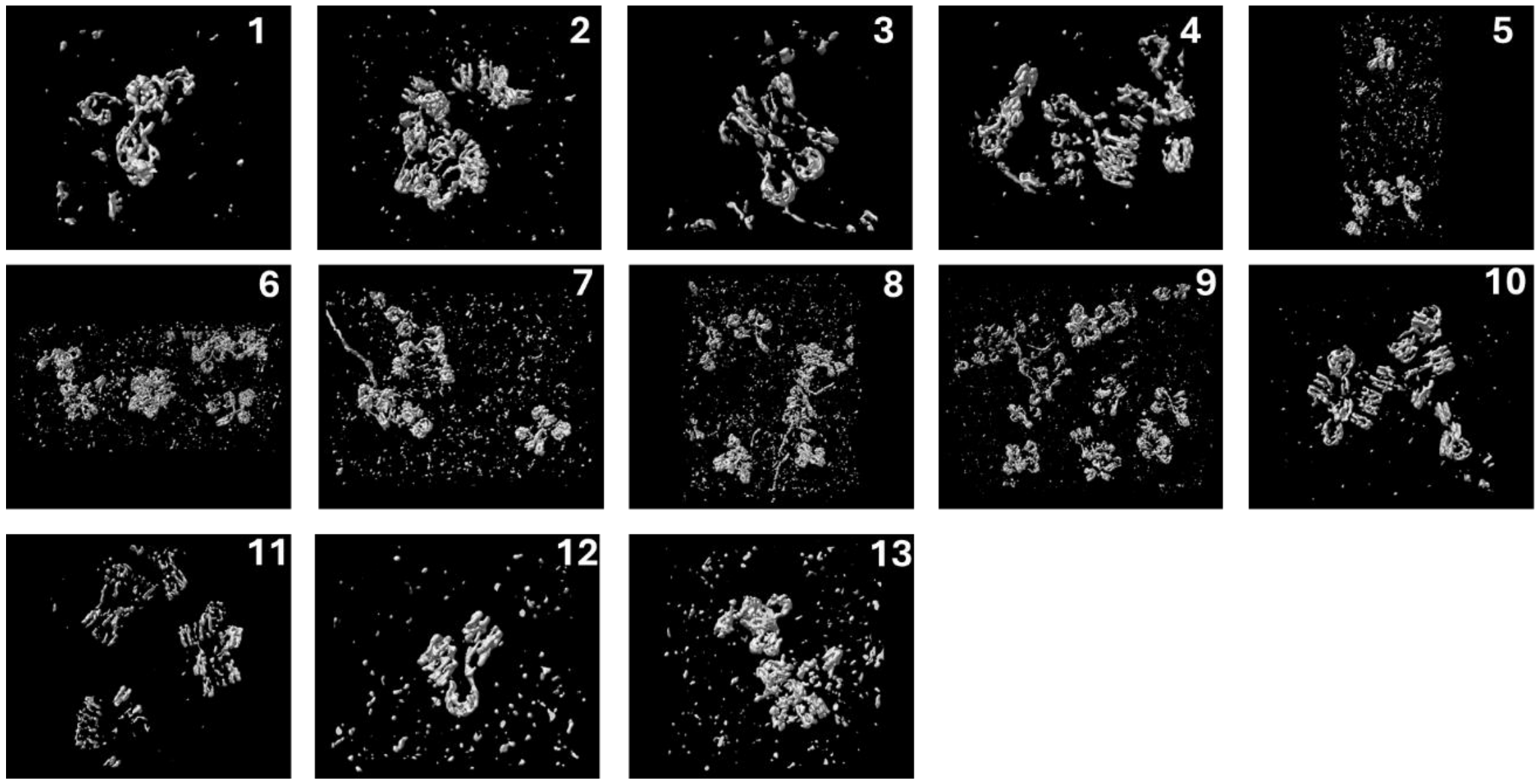

Figure S5. Comparisons of goodness-of-fit measures (correlation coefficients) among tri-nucleosome systems with and without histone tails and the mono-nucleosome system. Distributions of correlation coefficient values for fitting of NUC^{22}_{notail}, NUC^{22}_{tail} and mono-nucleosome (PDB: 1KX5) into the high-density regions of Cryo-ET maps of native chromatin vitrified at (a) 0.0 mM Mg^{2+} and (b) 0.75 mM Mg^{2+}. Each data point represents the fitting correlation coefficient value (n(NUC^{22}_{notail}) = 4199, n(NUC^{22}_{tail}) = 4199, n(mono-nucleosome) = 197). Because the data distributions were normal, statistical significance was assessed using ANOVA followed by Tukey's post-hoc test. Significance levels are indicated as $p < 0.05$ (*), $p < 0.01$ (**), $p < 0.001$ (***), and $p < 0.0001$ (****).

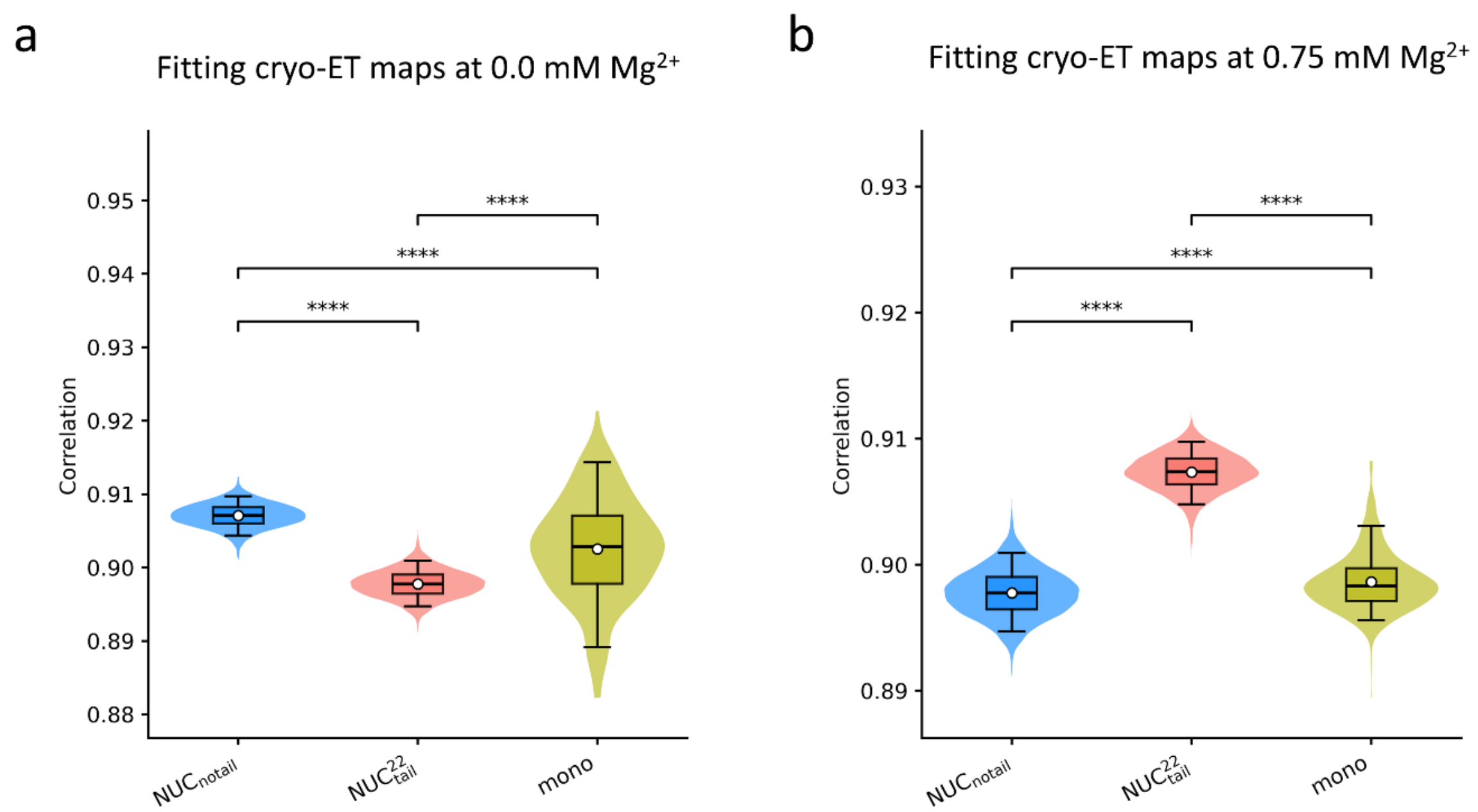

Figure S6. Comparisons of goodness-of-fit measures (inside-the-contour) among tri-nucleosome systems with and without histone tails and the mono-nucleosome system. Distributions of inside-the-contour values for fitting of $\mathrm{NUC}^{22}_{\mathrm{notail}}$, $\mathrm{NUC}^{22}_{\mathrm{tail}}$ and mono-nucleosome (PDB: 1KX5) into the high-density regions of Cryo-ET maps of native chromatin vitrified at (a) 0.0 mM Mg^{2+} and (b) 0.75 mM Mg^{2+}. Each data point represents the fitting inside-the-contour value (n($\mathrm{NUC}^{22}_{\mathrm{notail}}$) = 4199, n($\mathrm{NUC}^{22}_{\mathrm{tail}}$) = 4199, n(mono-nucleosome) = 197). Because the data distributions were non-normal, statistical significance was assessed using the Kruskal-Wallis test followed by pairwise Mann-Whitney U tests. Significance levels are indicated as $p < 0.05$ (*), $p < 0.01$ (**), $p < 0.001$ (***), and $p < 0.0001$ (****).

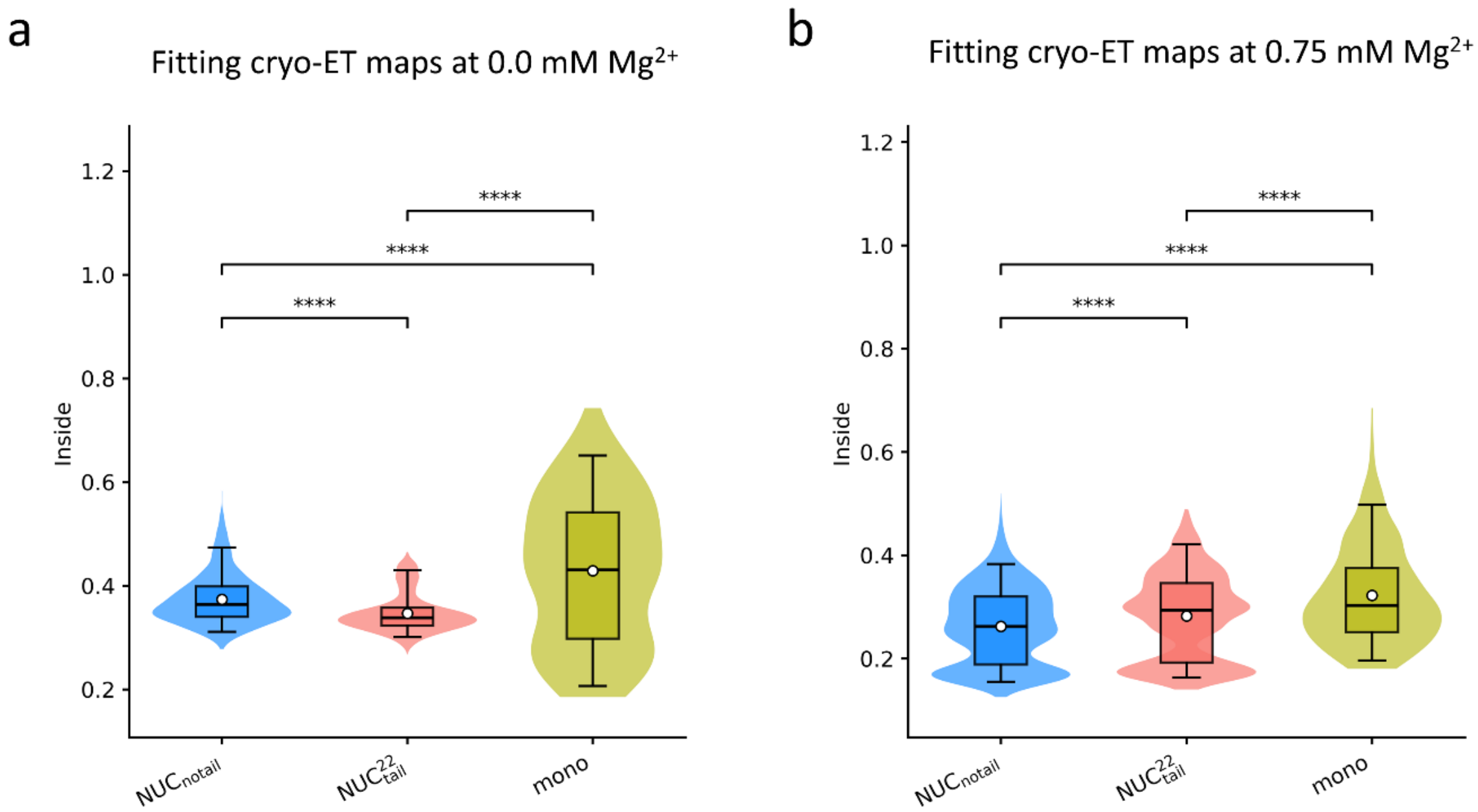

Figure S7. Comparisons of goodness-of-fit measures (correlation coefficient) among tri-nucleosome systems with different linker DNA lengths. Distributions of correlation coefficient values for fitting of NUC_{tail}^{22}, NUC_{tail}^{26} and NUC_{tail}^{31} into the high-density regions of Cryo-ET maps of native chromatin vitrified at (a) 0.0 mM Mg^{2+} and (b) 0.75 mM Mg^{2+}. Each data point represents the fitting correlation coefficient value ($n(NUC_{tail}^{22}) = 4199$, $n(NUC_{tail}^{26}) = 4199$, $n(NUC_{tail}^{31}) = 4199$). Because the data distributions were normal, statistical significance was assessed using ANOVA followed by Tukey's post-hoc test. Significance levels are indicated as $p < 0.05$ (*), $p < 0.01$ (**), $p < 0.001$ (***), and $p < 0.0001$ (****).

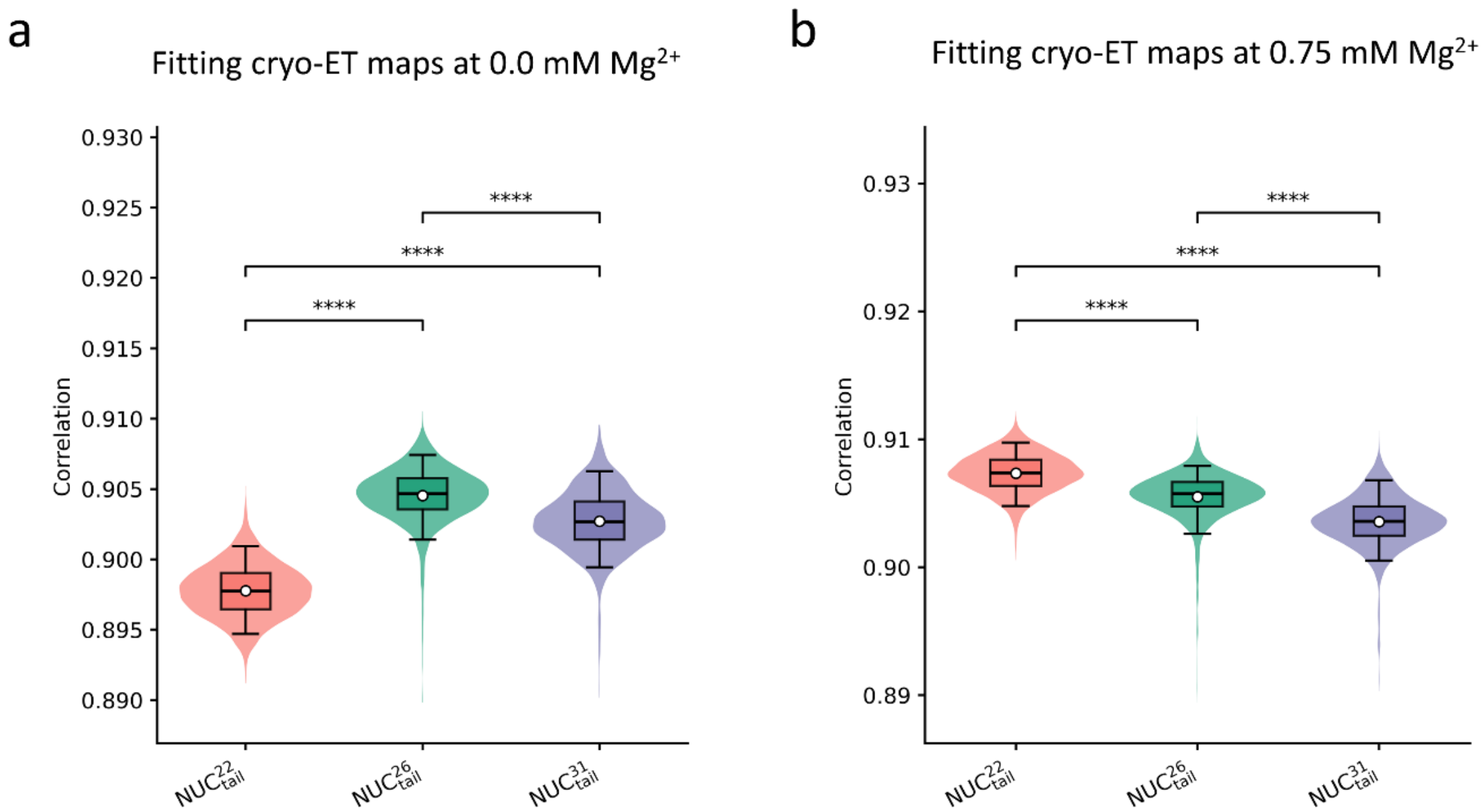

Figure S8. Comparisons of goodness-of-fit measures (inside-the-contour) among tri-nucleosome systems with different linker DNA lengths. Distributions of inside-the-contour values for fitting of $\mathrm{NUC}^{22}_{\mathrm{tail}}$, $\mathrm{NUC}^{26}_{\mathrm{tail}}$ and $\mathrm{NUC}^{31}_{\mathrm{tail}}$ into the high-density regions of Cryo-ET maps of native chromatin vitrified at (a) 0.0 mM Mg^{2+} and (b) 0.75 mM Mg^{2+}. Each data point represents the fitting inside-the-contour value ($n(\mathrm{NUC}^{22}_{\mathrm{tail}}) = 4199$, $n(\mathrm{NUC}^{26}_{\mathrm{tail}}) = 4199$, $n(\mathrm{NUC}^{31}_{\mathrm{tail}}) = 4199$). Because the data distributions were non-normal, statistical significance was assessed using the Kruskal-Wallis test followed by pairwise Mann-Whitney U tests. Significance levels are indicated as $p < 0.05$ (*), $p < 0.01$ (**), $p < 0.001$ (***), and $p < 0.0001$ (****).

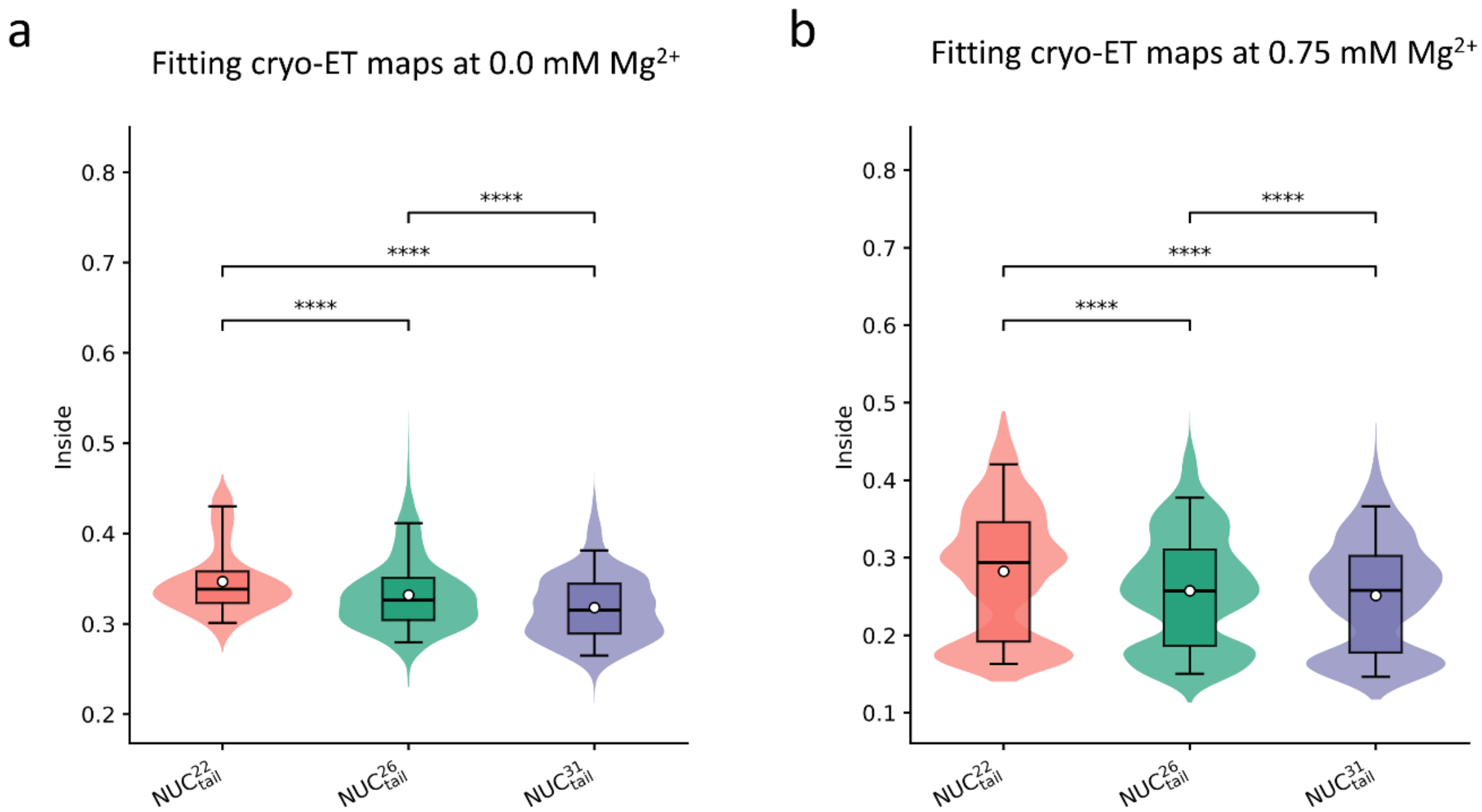

Figure S9. Quantitative comparisons of the orientation and positions of the fitted structural models using three denoising filters: NAD filter, AI-based denoising, and Gaussian filter. Each data point represents the measured distance or angle value (n(NAD filter) = 30, n(AI-based denoising) = 30, n(Gaussian filter) = 30). Significance levels are indicated as $p > 0.05$ (n.s.).

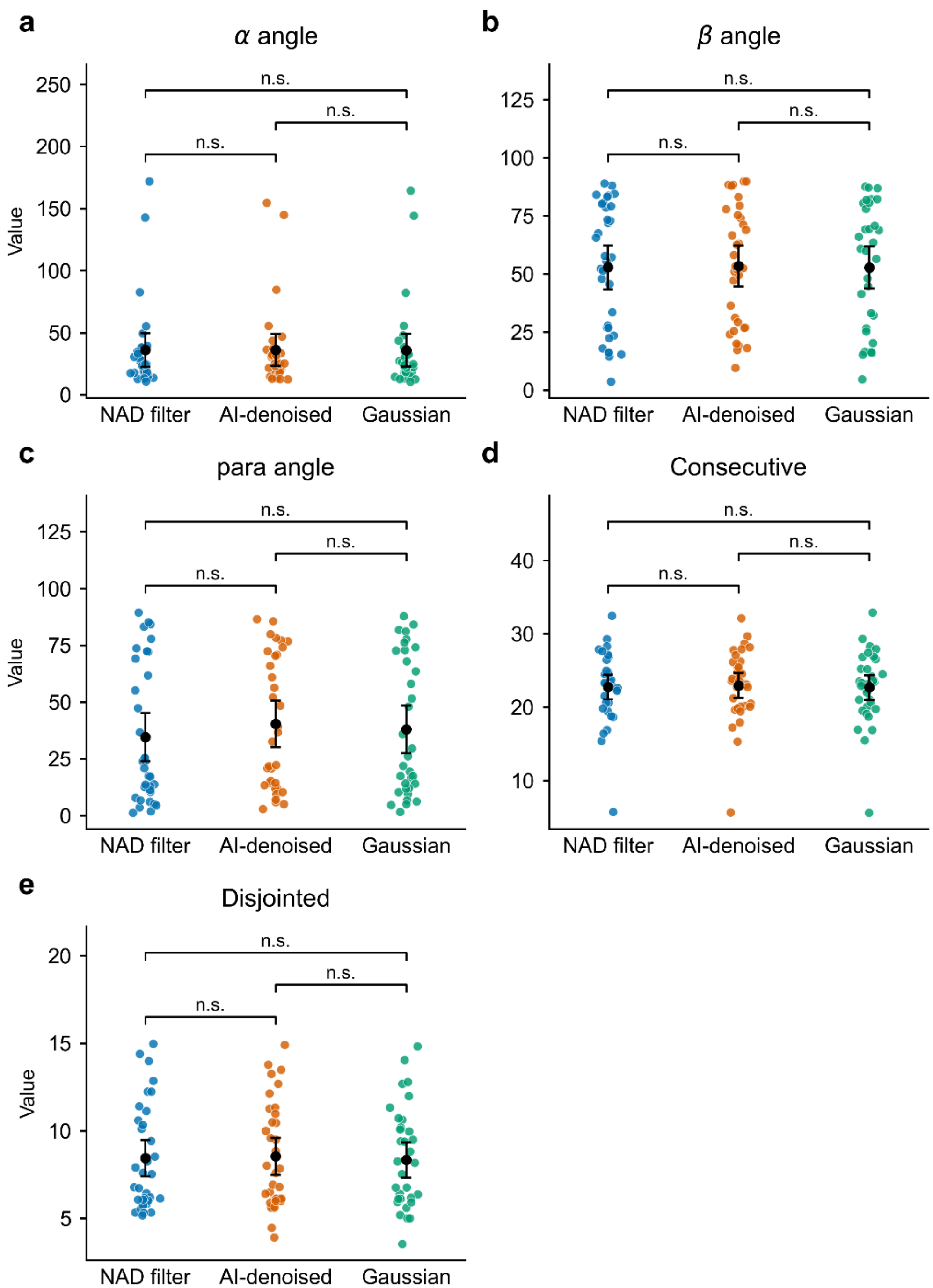

Figure S10. Fitting of a mono-nucleosome (PDB: 1KX5) into the high-density regions of Cryo-ET maps of the native chromatin vitrified at 0 mM Mg^{2+}.

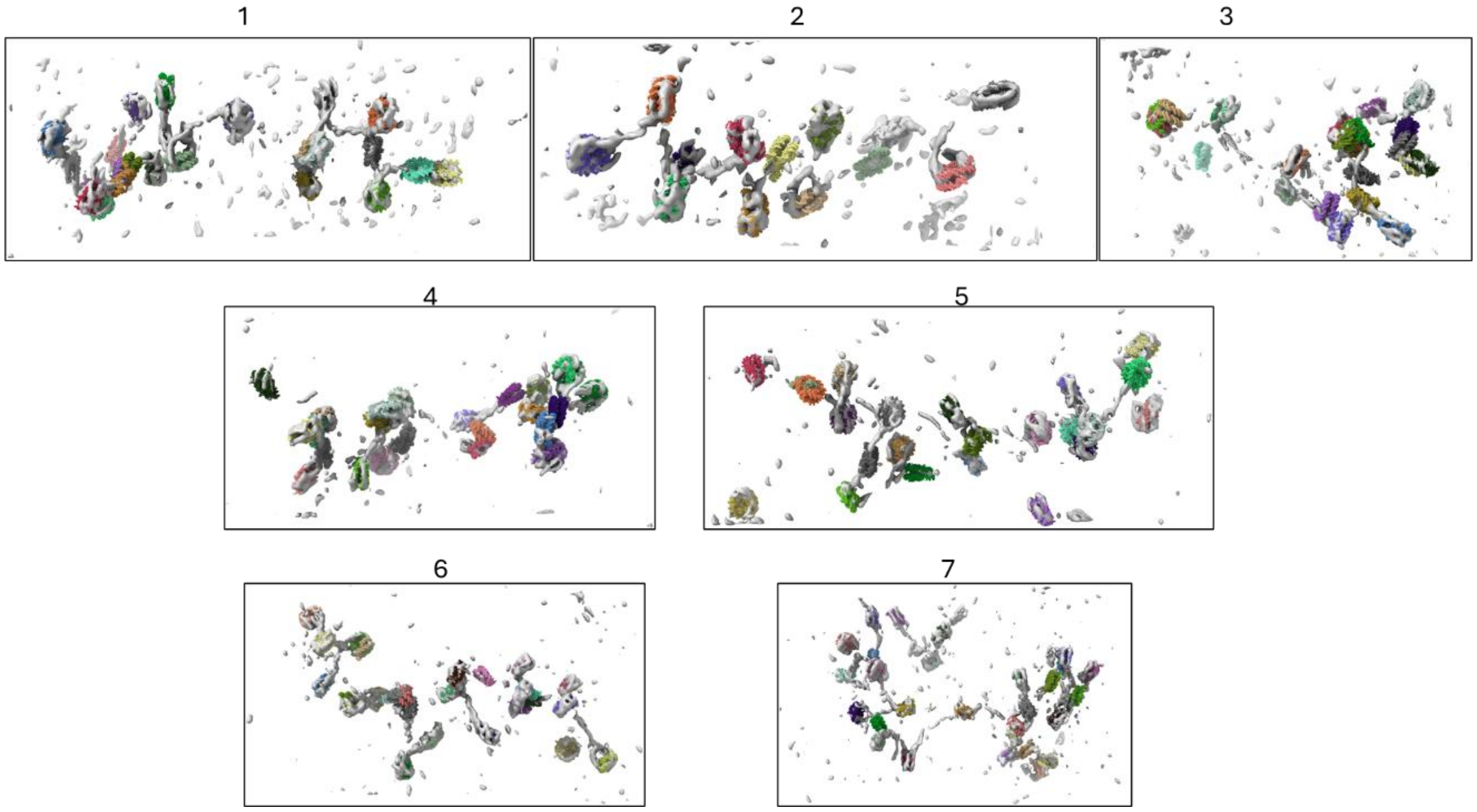

Figure S11. Fitting of a mono-nucleosome (PDB: 1KX5) into the high-density regions of Cryo-ET maps of native chromatin vitrified at 0.75 mM Mg^{2+}.

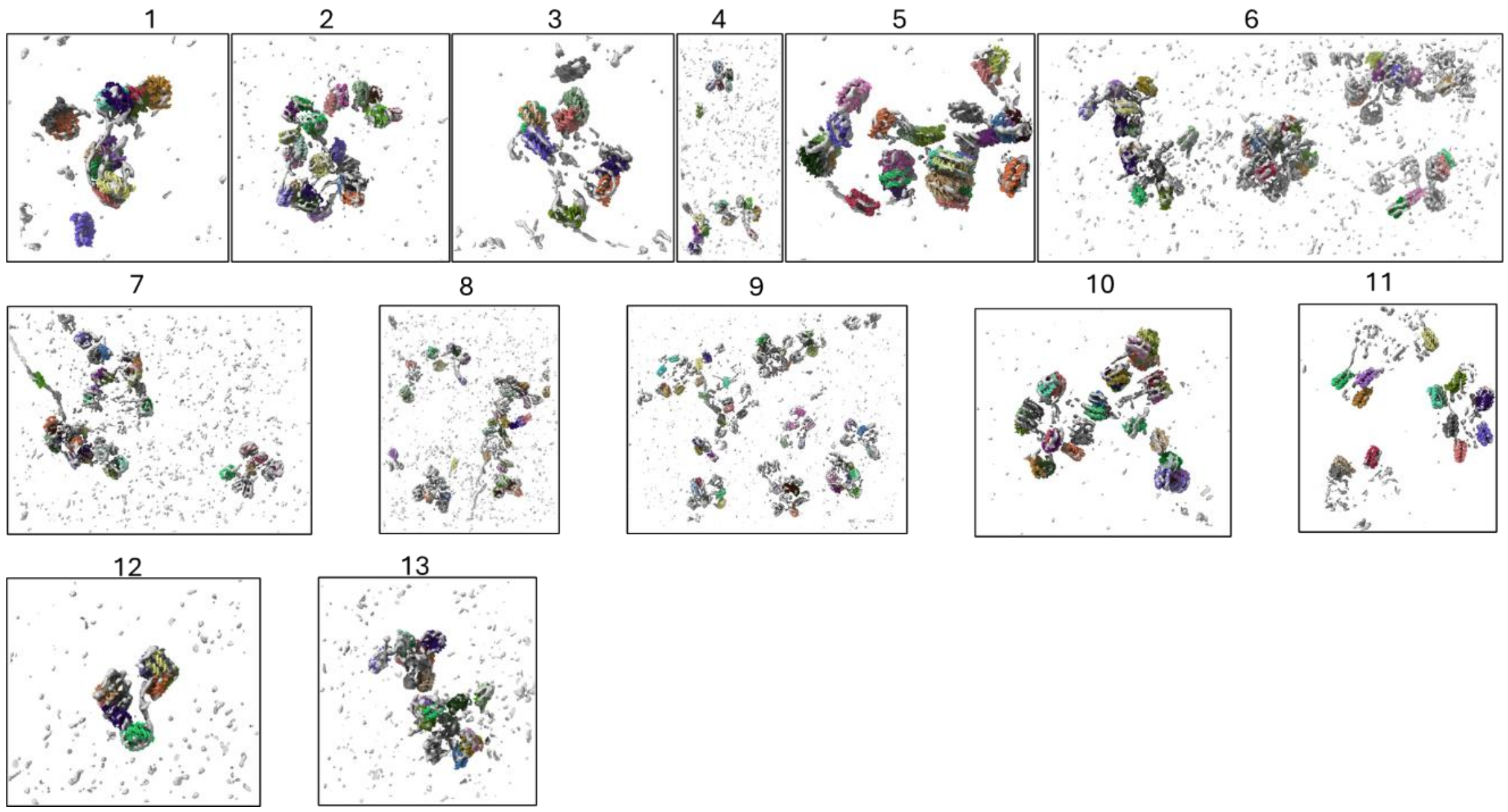

Figure S12. Representative and initial conformations (after equilibration) of tri-nucleosome MD simulations with different linker DNA lengths. (a) NUC_{tail}^{22} with a 22 bp linker. (b) NUC_{tail}^{26} with a 26 bp linker. (c) NUC_{tail}^{31} with a 31 bp linker.

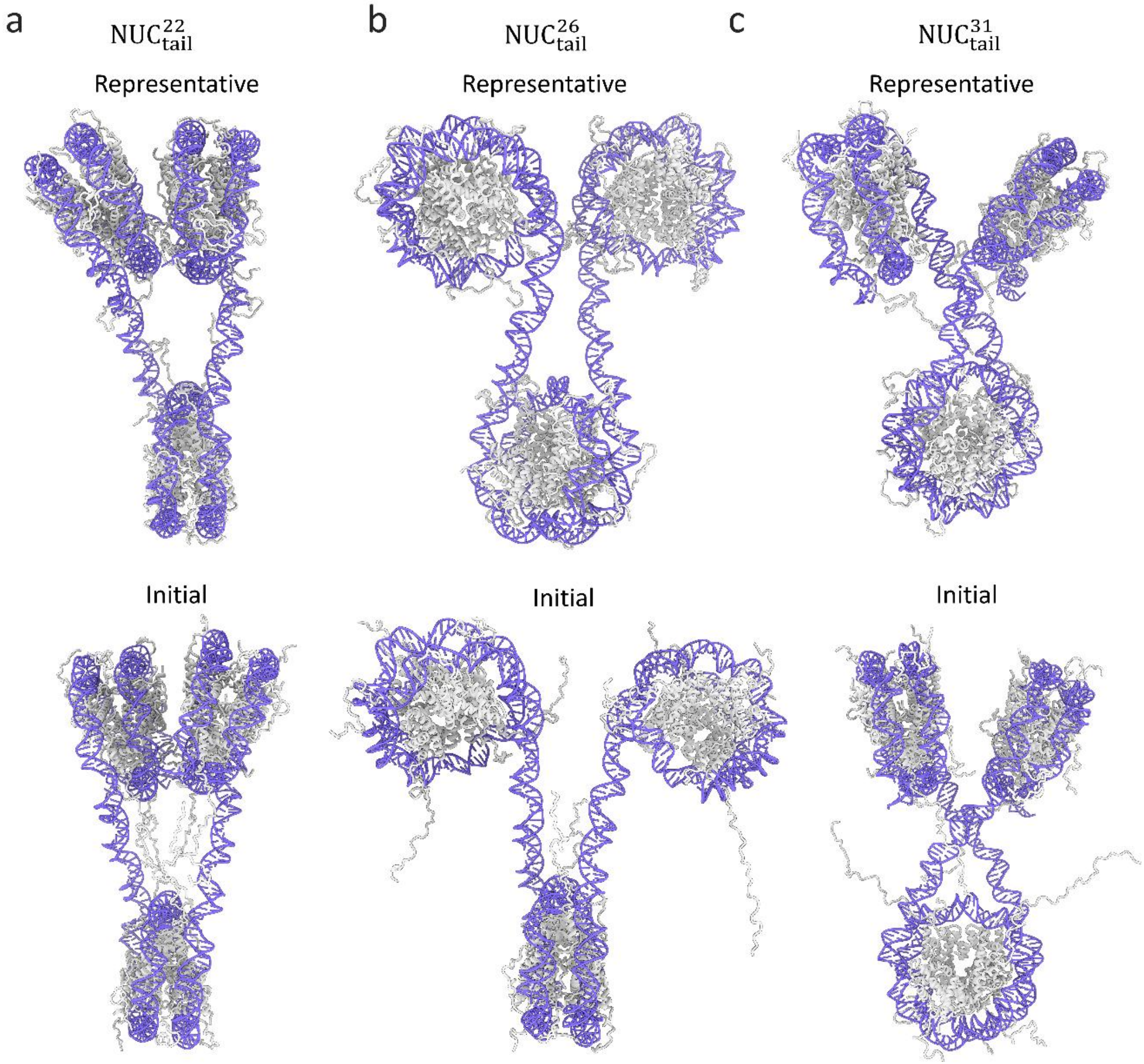

Figure S13. Comparisons of structural characteristics among tri-nucleosome systems with different linker DNA lengths. Distributions of disjointed (a) and consecutive (b) center-to-center nucleosome distances, β angles (c) and para angles (d) calculated from the MD simulations and experimental chromatin array vitrified at 0.75 mM Mg^{2+}. All simulations were performed with histone tails included.

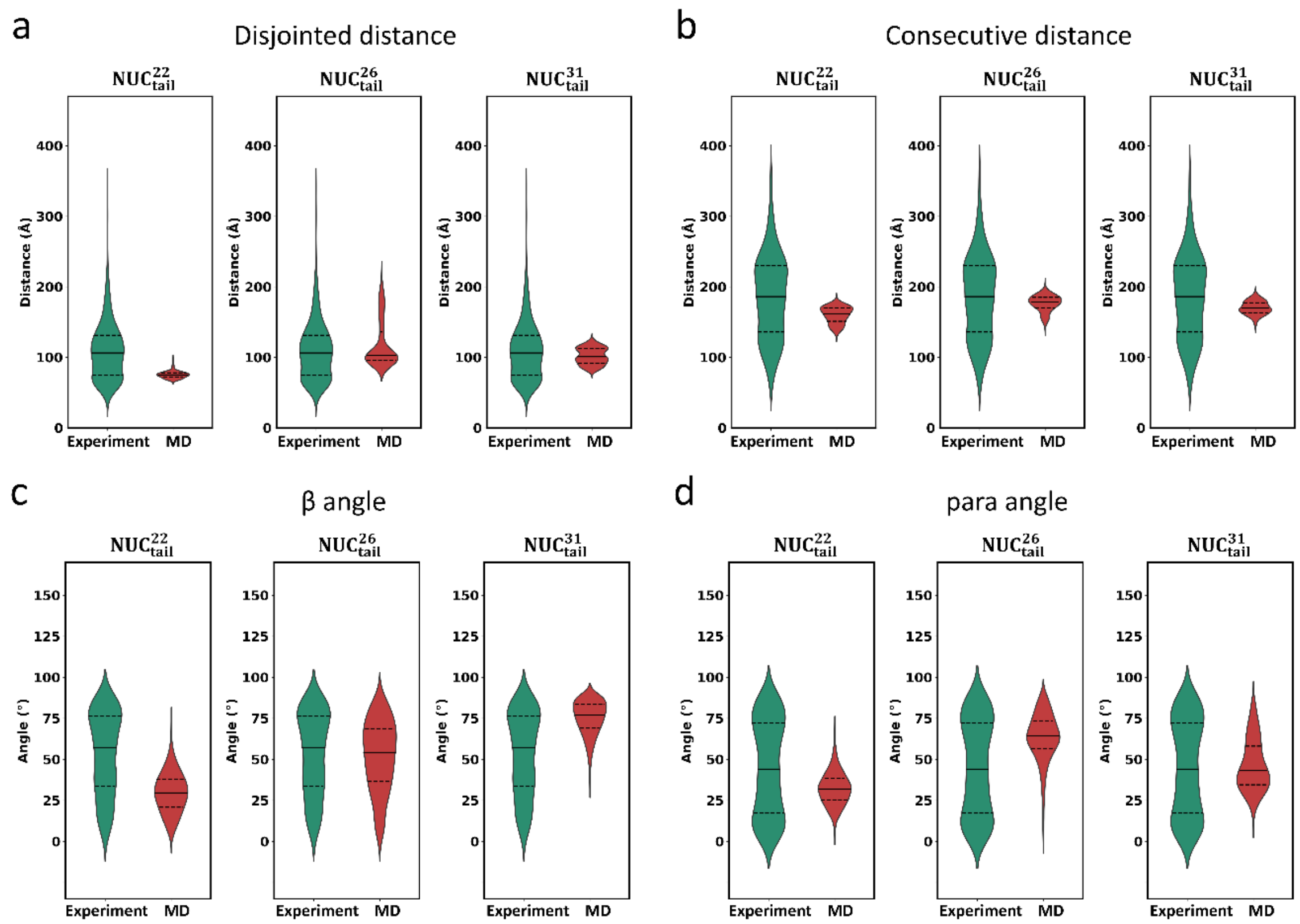

Figure S14. Representative conformations of NUC^{22}_{notail} simulations, showing unfolding of nucleosome 1 (Nuc1) (a), nucleosome 3 (Nuc3) (b), or both nucleosome 1 and nucleosome 3 (c) from the middle nucleosome (Nuc2).

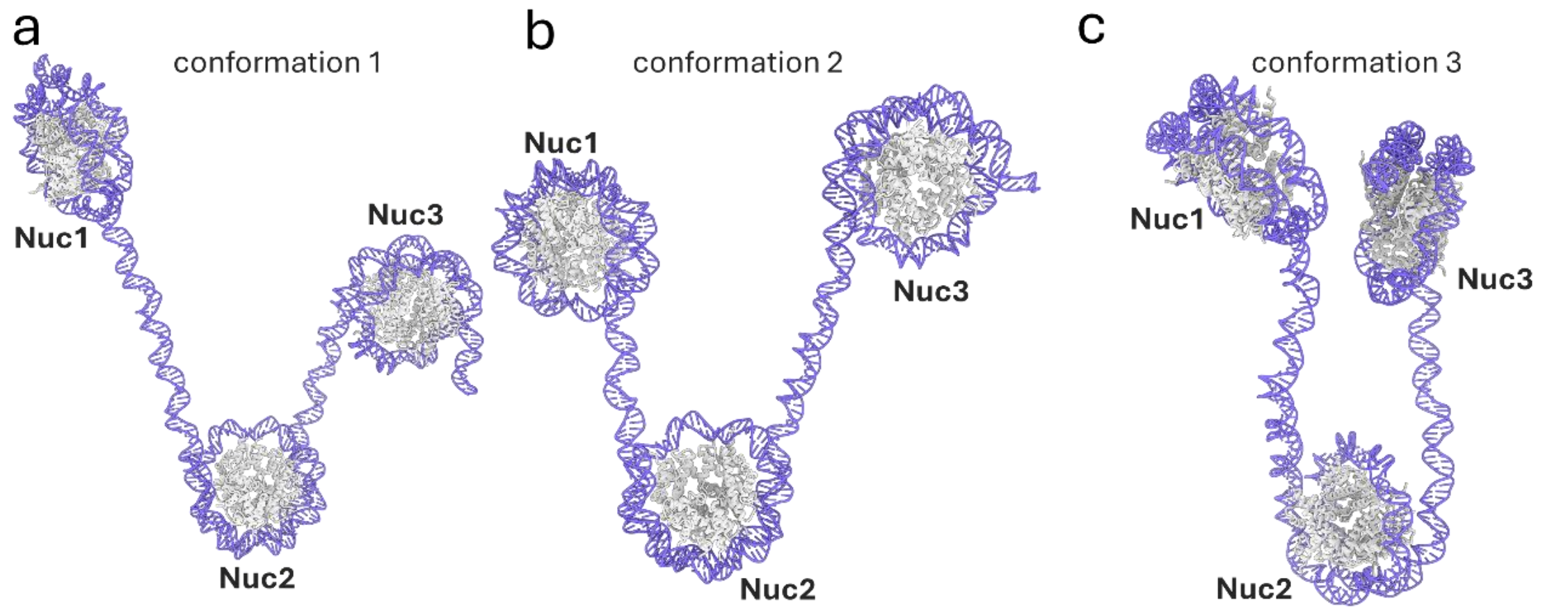

Figure S15. A free-energy surface as a function of center-to-center distances between nucleosome 1 (Nuc1) and nucleosome 2 (Nuc2), and between nucleosome 2 and nucleosome 3 (Nuc3) in $\mathrm{NUC}_{\mathrm{tail}}^{22}$ simulations. Free energies are in kcal/mol.

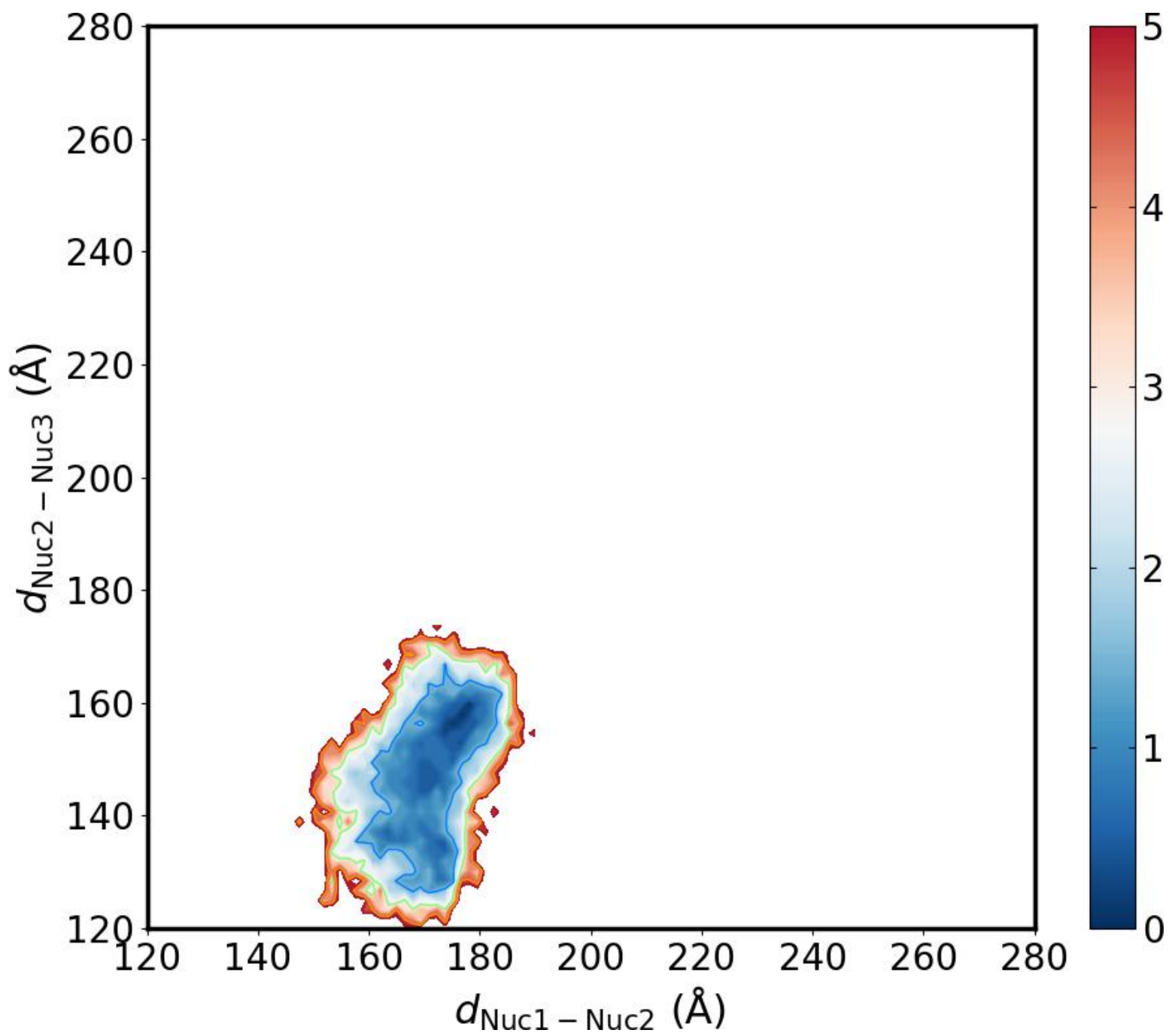

Figure S16. Comparisons of inter-nucleosomal histone tail-tail contacts among tri-nucleosome systems with different linker DNA lengths. The average number per frame of inter-nucleosomal histone tail-tail contacts in the $\mathrm{NUC}_{\mathrm{tail}}^{22}$ (a), $\mathrm{NUC}_{\mathrm{tail}}^{26}$ (b) and $\mathrm{NUC}_{\mathrm{tail}}^{31}$ (c) systems. The inter-nucleosomal histone tail-tail contacts are contacts between nucleosomes Nuc1-Nuc2, Nuc2-Nuc3 and Nuc1-Nuc3. Data are presented as mean values ± SEM from three independent simulation runs (n = 3).

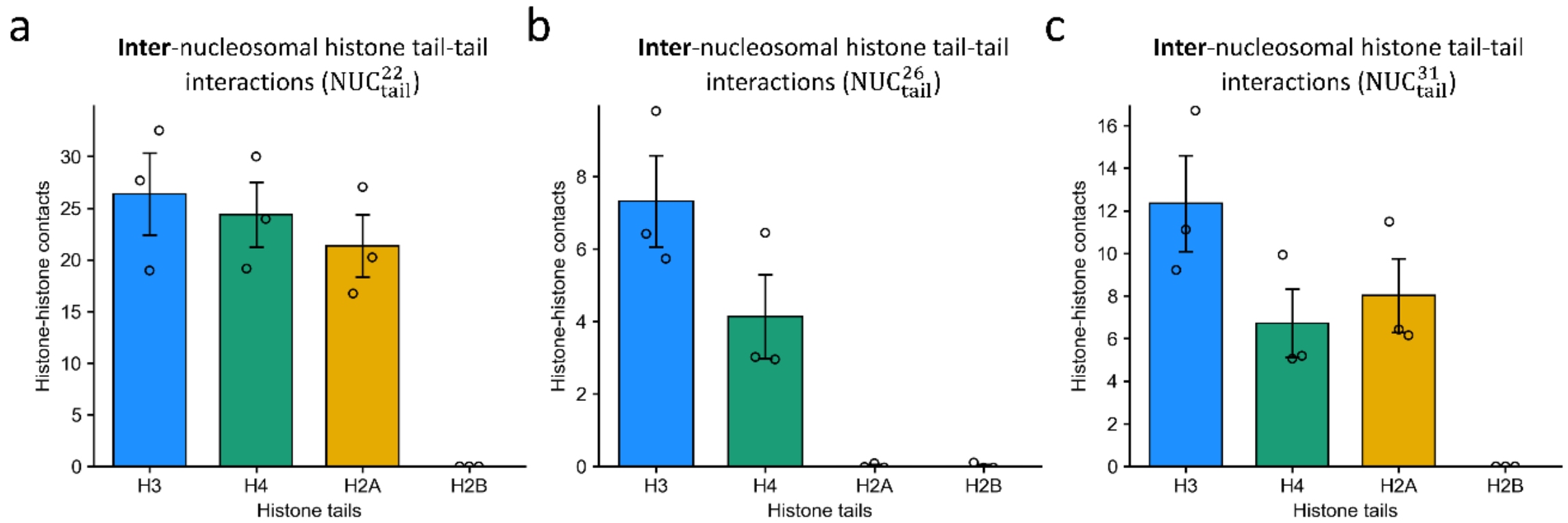

Figure S17. Comparisons of intra- and inter-nucleosomal histone tail–DNA contacts among tri-nucleosome systems with different linker DNA lengths. **a-c:** The average number of intra-nucleosomal histone tail-DNA contacts (histone tails interacting with the DNA from their originating nucleosome) in the $\mathrm{NUC}_{\mathrm{tail}}^{22}$ (a), $\mathrm{NUC}_{\mathrm{tail}}^{26}$ (b) and $\mathrm{NUC}_{\mathrm{tail}}^{31}$ (c) systems. **d-f:** The average number of inter-nucleosomal histone tail-DNA contacts in the $\mathrm{NUC}_{\mathrm{tail}}^{22}$ (d), $\mathrm{NUC}_{\mathrm{tail}}^{26}$ (e) and $\mathrm{NUC}_{\mathrm{tail}}^{31}$ (f) systems. The inter-nucleosomal histone tail-DNA contacts are contacts between histone tails from Nuc1 and DNA from Nuc2 and Nuc3, histone tails from Nuc2 and DNA from Nuc1 and Nuc3, and histone tails from Nuc3 and DNA from Nuc1 and Nuc2. Data are presented as mean values ± SEM from three independent simulation runs (n = 3).

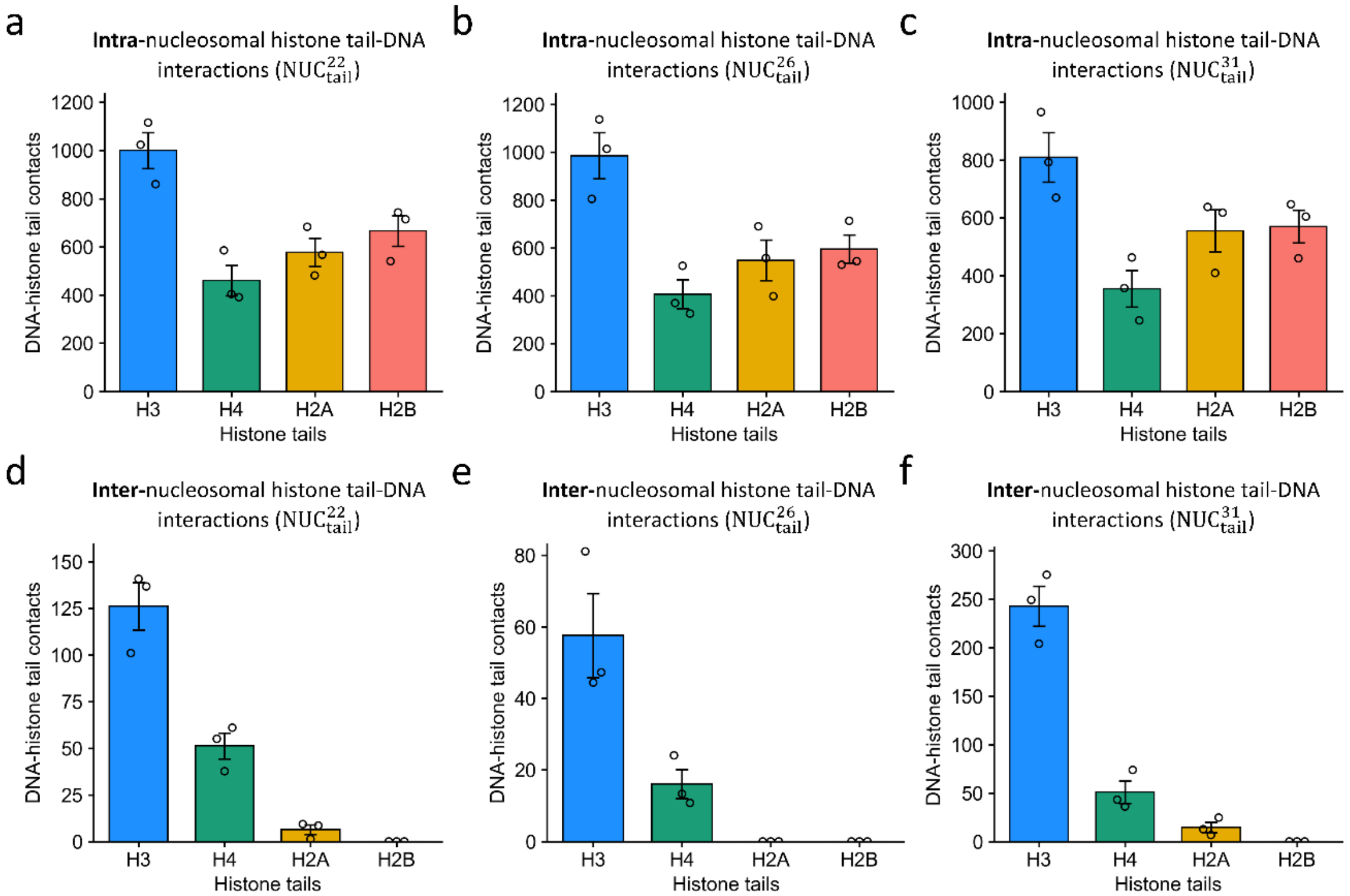

Figure S18. Contact maps show the percentage of all contacts between the H3 N-terminal tail from Nuc1 and the H4 N-terminal tail from Nuc3 in each MD simulation run in the $\mathrm{NUC}_{\mathrm{tail}}^{22}$ system.

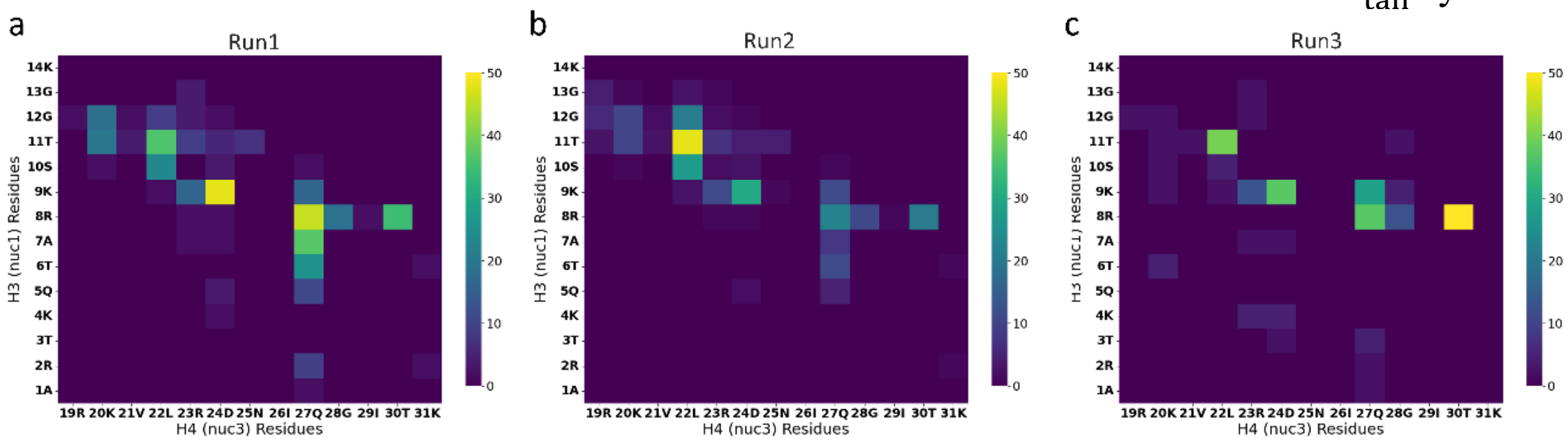

Figure S19. Contact maps show the percentage of all contacts between the H4 N-terminal tail from Nuc1 and the H2A C-terminal tail from Nuc3 in each MD simulation run in the NUC_{tail}^{22} system.

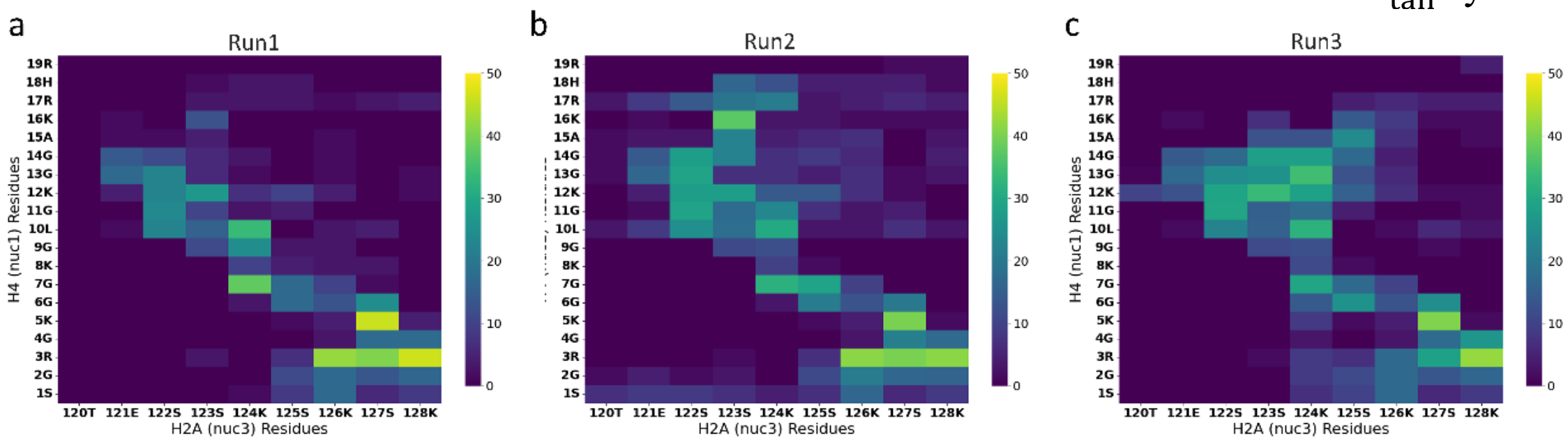

Figure S20. Contact maps show the percentage of all contacts between the H3 N-terminal tail from Nuc2 and the H3 N-terminal tail from Nuc3 in each MD simulation run in the $\mathrm{NUC}_{\mathrm{tail}}^{22}$ system.

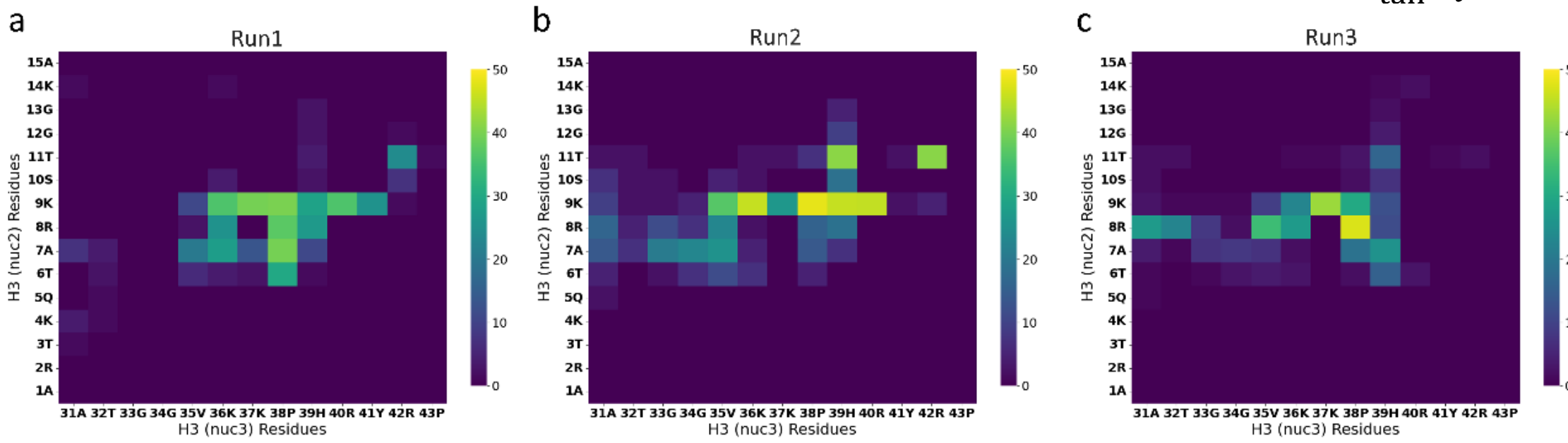

Figure S21. Mapping of histone tail–DNA contacts along DNA superhelical locations (SHLs). The mean numbers of contacts between the H4 (a), H3 (b), H2A (c), and H2B (d) tails and DNA, averaged across three independent simulation runs, are plotted as a function of SHL for each nucleosome in the NUC^{22}_{tail} simulations.. SHL 0 corresponds to the dyad position of each nucleosome.

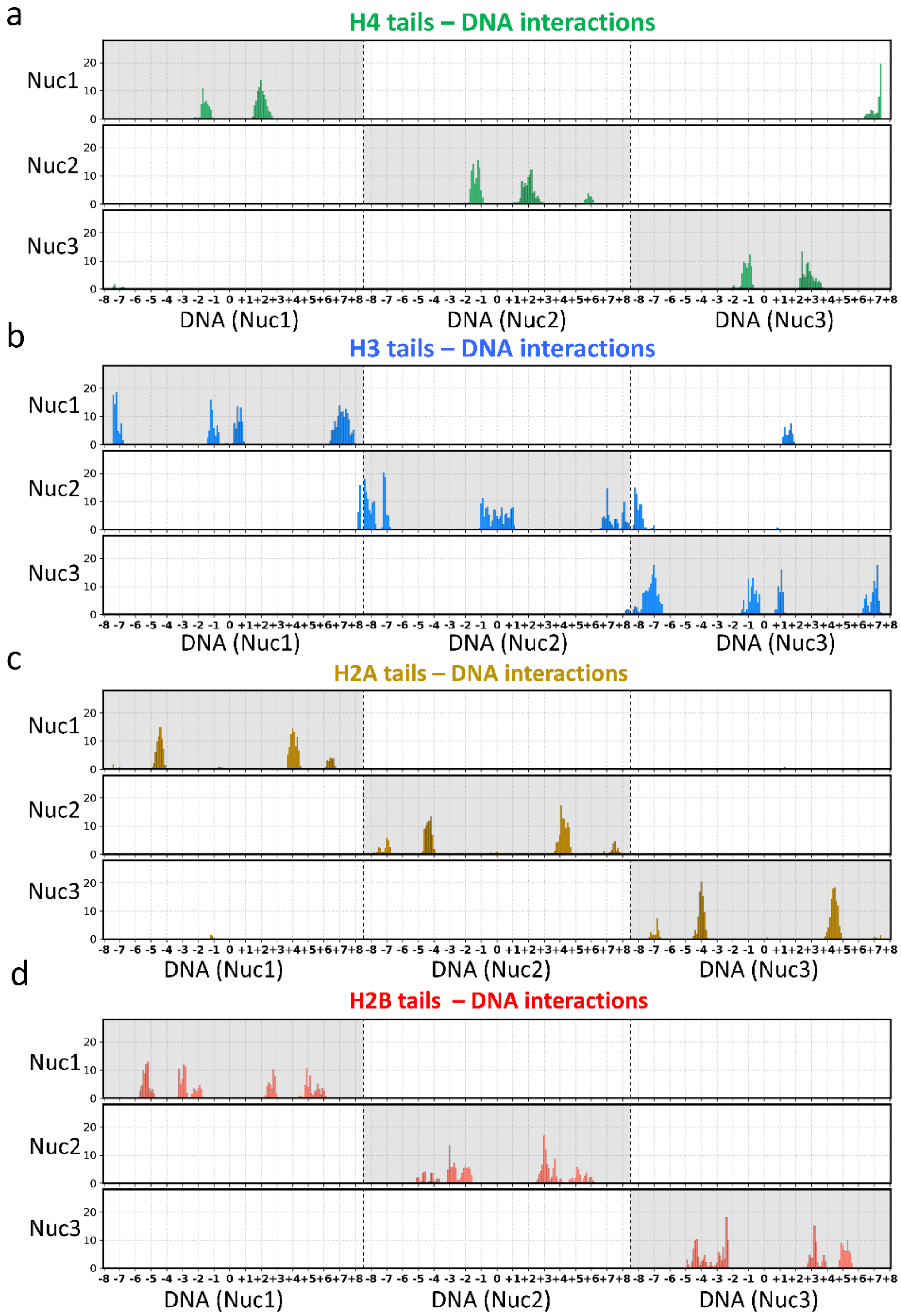

Figure S22. Mapping of histone tail–DNA contacts along DNA superhelical locations (SHLs). The mean numbers of contacts between the H4 (a), H3 (b), H2A (c), and H2B (d) tails and DNA, averaged across three independent simulation runs, are plotted as a function of SHL for each nucleosome in the NUC^{26}_{tail} simulations.. SHL 0 corresponds to the dyad position of each nucleosome.

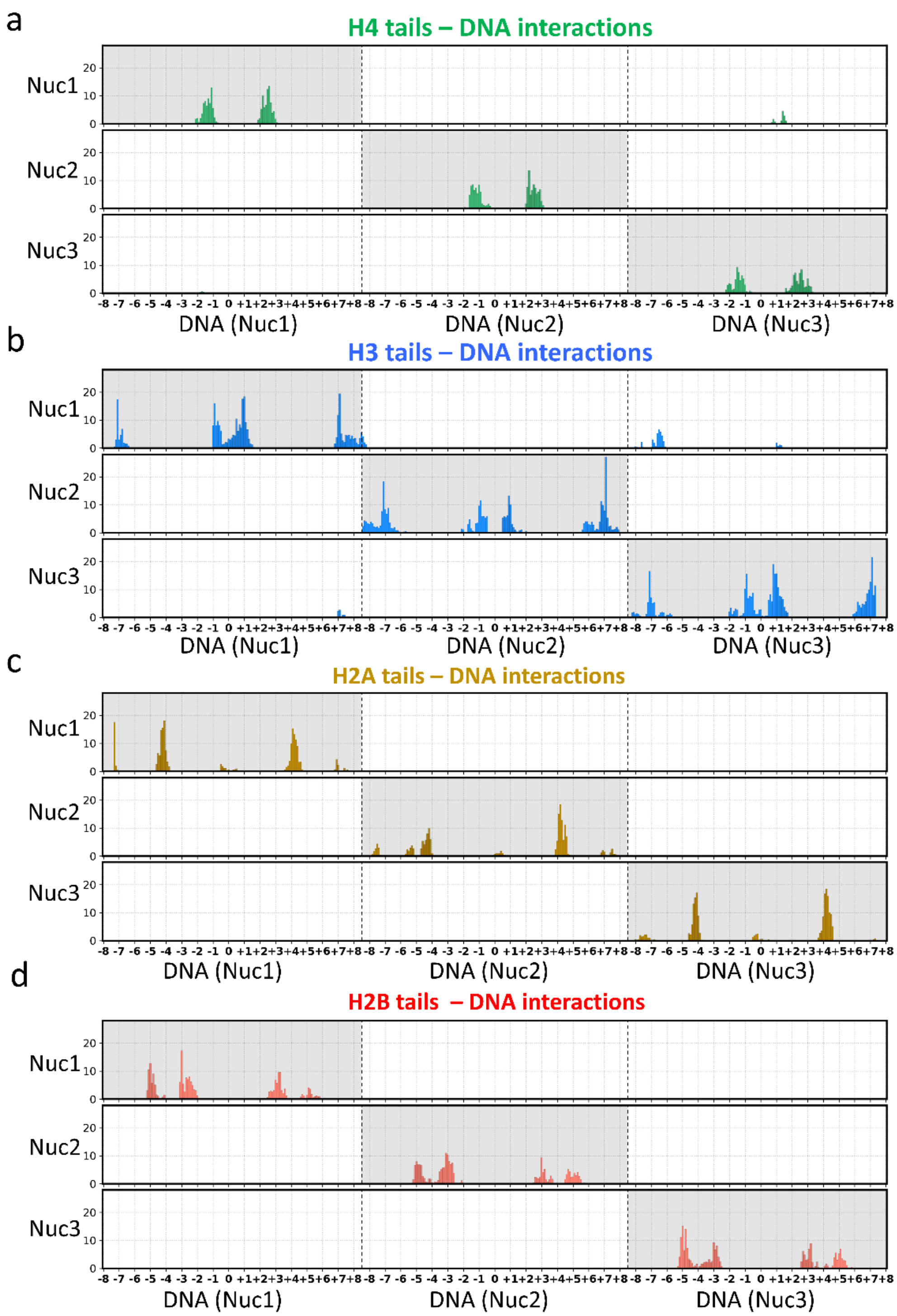

Figure S23. Mapping of histone tail–DNA contacts along DNA superhelical locations (SHLs). The mean numbers of contacts between the H4 (a), H3 (b), H2A (c), and H2B (d) tails and DNA, averaged across three independent simulation runs, are plotted as a function of SHL for each nucleosome in the NUC_{tail}^{31} simulations.. SHL 0 corresponds to the dyad position of each nucleosome.

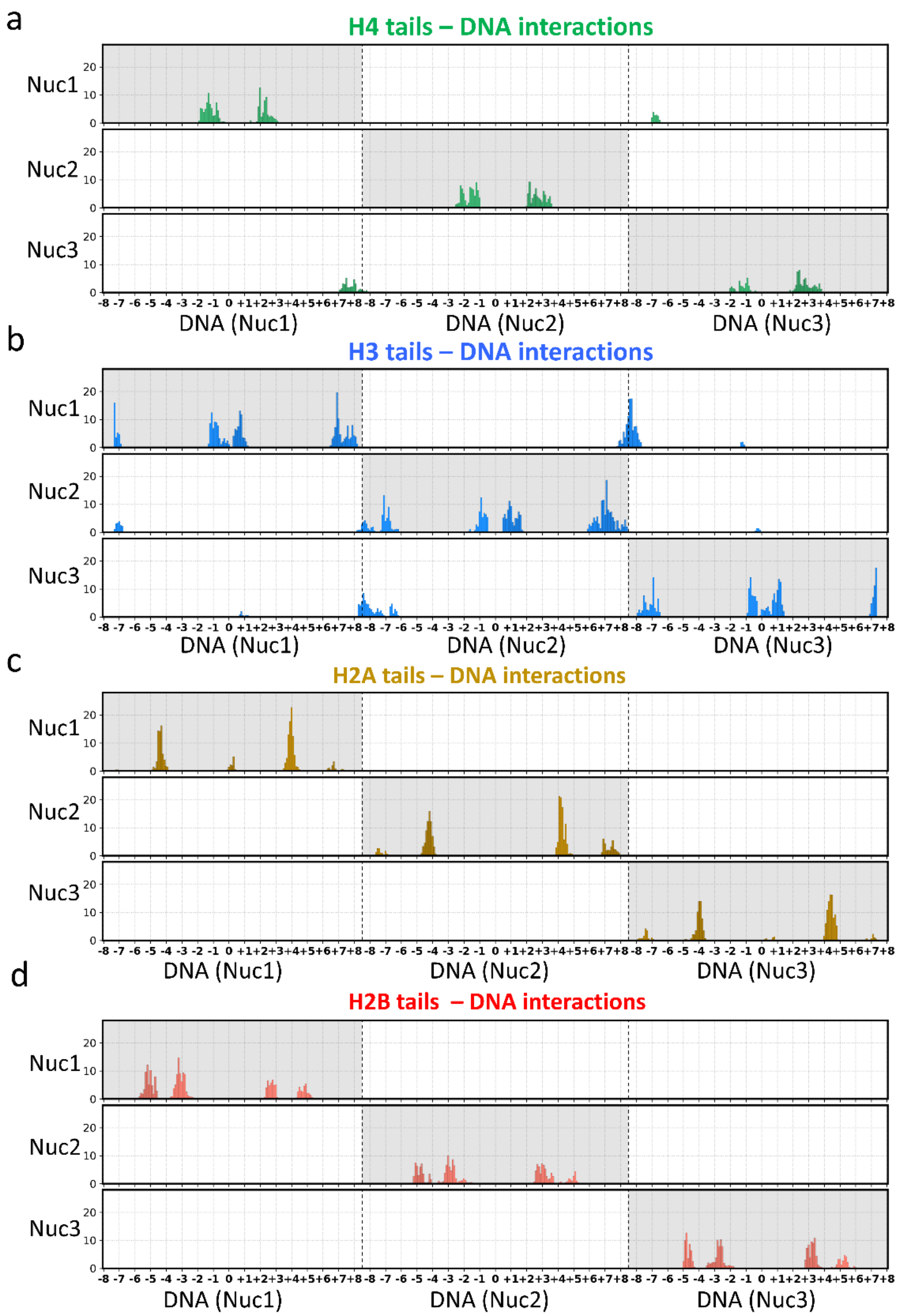

Figure S24. DNA sequences of the three tri-nucleosome models. (a) The DNA sequence (Homo sapiens TP53; GenBank ID: EU877045.1, range: 2486 to 2970) was used to construct the initial $\mathrm{NUC}_{\mathrm{tail}}^{22}$ tri-nucleosome model. (b) The DNA sequence for the $\mathrm{NUC}_{\mathrm{tail}}^{26}$ tri-nucleosome model. (c) The DNA sequence for the $\mathrm{NUC}_{\mathrm{tail}}^{31}$ tri-nucleosome model.

a

TTTGCTTCCTCCGGCAGGCGGATTACTTGCCCTTACTTGTCATGGCGACTGTCC
AGCTTTGTGCCAGGAGCCTCGCAGGGGTTGATGGGATTGGGGTTTTCCCCTCCC
ATGTGCTCAAGACTGGCGCTAAAAGTTTTGAGCTTCTCAAAAGTCTAGAGCCAC
CGTCCAGGGAGCAGGTAGCTGCTGGGCTCCGGGGACACTTTGCGTTCGGGCTGG
GAGCGTGCTTTCCACGACGGTGACACGCTTCCCTGGATTGGGTAAGCTCCTGAC
TGAACTTGATGAGTCCTCTCTGAGTCACGGGCTCTCGGCTCCGTGTATTTTCAG
CTCGGGAAAATCGCTGGGGCTGGGGGTGGGGCAGTGGGGACTTAACGAGTTTGG
GGGTGAGTGGGATGGAAGCTTGGCTAGAGGGATCATCATAGGAGTTGCATTGTT
GGGAGACCTGGGTGTAGATGATGGGGATGTTAGGACCATCCGAACTCAAAGTT

Nucleosomal DNA regions
Linker DNA regions

b

TTTGCTTCCTCCGGCAGGCGGATTACTTGCCCTTACTTGTCATGGCGACTGTCC
AGCTTTGTGCCAGGAGCCTCGCAGGGGTTGATGGGATTGGGGTTTTCCCCTCCC
ATGTGCTCAAGACTGGCGCTAAAAGTTTTGAGCTTCTCAAAAGTCTAGAGCCAC
CGTCCAGAAAGGGAGCAGGTAGCTGCTGGGCTCCGGGGACACTTTGCGTTCGGG
CTGGGAGCGTGCTTTCCACGACGGTGACACGCTTCCCTGGATTGGGTAAGCTCC
TGACTGAACTTGATGAGTCCTCTCTGAGTCACGGGCTCTCGGCTCCGTGTATTT
TCAGCTCGGGAAAATCGCATTTTGGGGCTGGGGGTGGGGCAGTGGGGACTTAAC
GAGTTTGGGGGTGAGTGGGATGGAAGCTTGGCTAGAGGGATCATCATAGGAGTT
GCATTGTTGGGAGACCTGGGTGTAGATGATGGGGATGTTAGGACCATCCGAACT
CAAAGTT

Nucleosomal DNA regions
Linker DNA regions

c

TTTGCTTCCTCCGGCAGGCGGATTACTTGCCCTTACTTGTCATGGCGACTGTCC
AGCTTTGTGCCAGGAGCCTCGCAGGGGTTGATGGGATTGGGGTTTTCCCCTCCC
ATGTGCTCAAGACTGGCGCTAAAAGTTTTGAGCTTCTCAAAAGTCTAGAGCCAC
CGTCCAGAAAGTCTAGGGAGCAGGTAGCTGCTGGGCTCCGGGGACACTTTGCGT
TCGGGCTGGGAGCGTGCTTTCCACGACGGTGACACGCTTCCCTGGATTGGGTAA
GCTCCTGACTGAACTTGATGAGTCCTCTCTGAGTCACGGGCTCTCGGCTCCGTG
TATTTTCAGCTCGGGAAAATCGCATTTTCAGCTGGGGCTGGGGGTGGGGCAGTG
GGGACTTAACGAGTTTGGGGGTGAGTGGGATGGAAGCTTGGCTAGAGGGATCAT
CATAGGAGTTGCATTGTTGGGAGACCTGGGTGTAGATGATGGGGATGTTAGGAC
CATCCGAACTCAAAGTT

Nucleosomal DNA regions
Linker DNA regions

Figure S25. The constructed models (before energy minimization and equilibration) for different lengths of linker DNA. (a) 22 bp linker DNA. (b) 26 bp linker DNA. (c) 31 bp linker DNA.

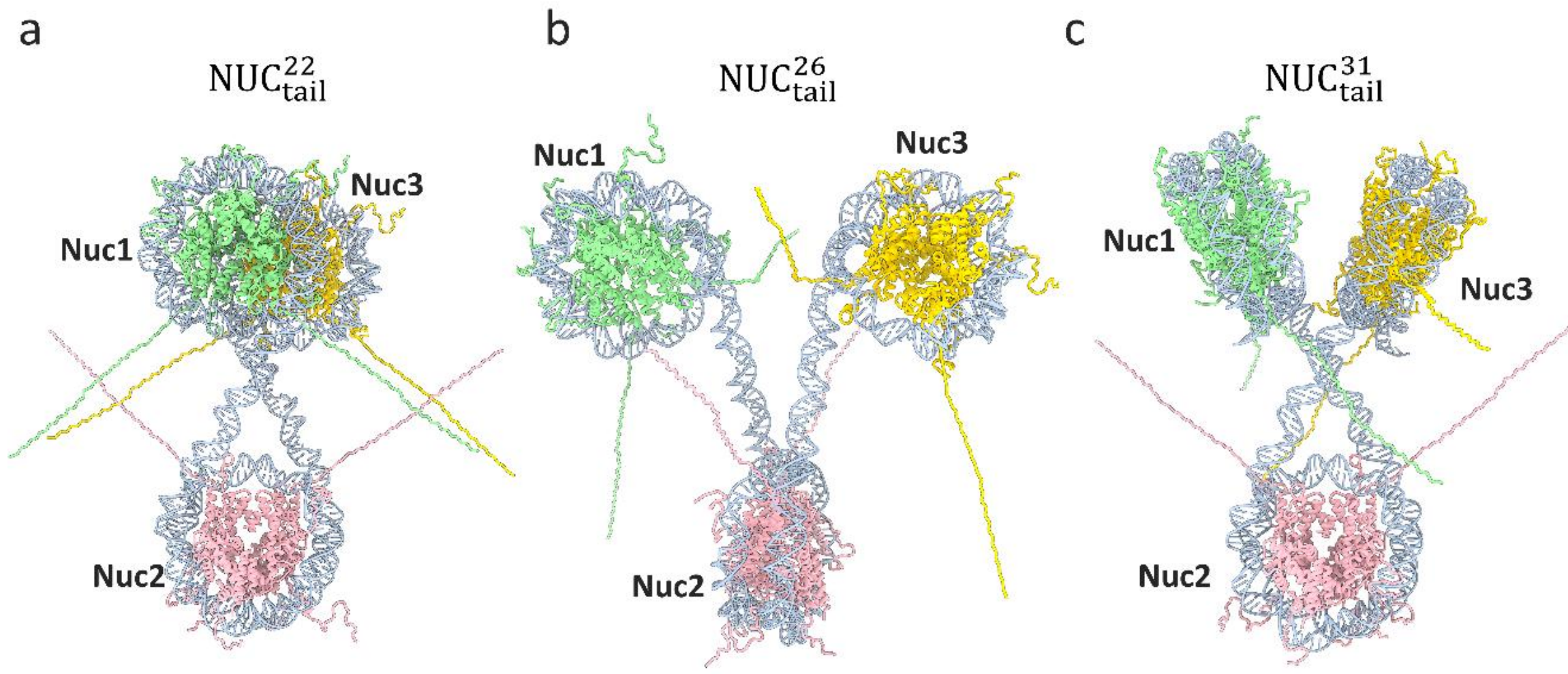

References

1. Jentink N, Purnell C, Kable B, Swulius MT, Grigoryev SA. Cryoelectron tomography reveals the multiplex anatomy of condensed native chromatin and its unfolding by histone citrullination. *Mol Cell* **83**, 3236–3252 (2023).
