# Supplementary material for: Molecular dynamics guided all-atom reconstruction of cryo-ET maps reveals mechanisms of histone tail mediated chromatin compaction
Source: Nat Commun. 2026 Sep 15;17:10811. doi: 10.1038/s41467-026-77871-9 (PMC13669341; doi:10.1038/s41467-026-77871-9)
Supplement: Supplementary file 2 [file 41467_2026_77871_MOESM2_ESM.pdf]

Description of Additional Supplementary Files

Supplementary Data 1. The raw data for quantitative comparisons on the orientation and positions of the fitted structural models using three denoising filters: NAD filter, AI-based denoising, and Gaussian filter.
