# Supplementary material for: Molecular dynamics guided all-atom reconstruction of cryo-ET maps reveals mechanisms of histone tail mediated chromatin compaction
Source: Nat Commun. 2026 Sep 15;17:10811. doi: 10.1038/s41467-026-77871-9 (PMC13669341; doi:10.1038/s41467-026-77871-9)
Supplement: Supplementary file 3 [file 41467_2026_77871_MOESM4_ESM.pdf]

nature portfolio

Corresponding author(s): Anna Panchenko

Last updated by author(s): Aug 21, 2026

Reporting Summary

Nature Portfolio wishes to improve the reproducibility of the work that we publish. This form provides structure for consistency and transparency in reporting. For further information on Nature Portfolio policies, see our Editorial Policies and the Editorial Policy Checklist.

Statistics

For all statistical analyses, confirm that the following items are present in the figure legend, table legend, main text, or Methods section.

n/a	Confirmed	
[ ]	[x]	The exact sample size (*n*) for each experimental group/condition, given as a discrete number and unit of measurement
[ ]	[x]	A statement on whether measurements were taken from distinct samples or whether the same sample was measured repeatedly
[x]	[ ]	The statistical test(s) used AND whether they are one- or two-sided<br>*Only common tests should be described solely by name; describe more complex techniques in the Methods section.*
[x]	[ ]	A description of all covariates tested
[x]	[ ]	A description of any assumptions or corrections, such as tests of normality and adjustment for multiple comparisons
[ ]	[x]	A full description of the statistical parameters including central tendency (e.g. means) or other basic estimates (e.g. regression coefficient) AND variation (e.g. standard deviation) or associated estimates of uncertainty (e.g. confidence intervals)
[x]	[ ]	For null hypothesis testing, the test statistic (e.g. *F*, *t*, *r*) with confidence intervals, effect sizes, degrees of freedom and *P* value noted<br>*Give P values as exact values whenever suitable.*
[x]	[ ]	For Bayesian analysis, information on the choice of priors and Markov chain Monte Carlo settings
[x]	[ ]	For hierarchical and complex designs, identification of the appropriate level for tests and full reporting of outcomes
[x]	[ ]	Estimates of effect sizes (e.g. Cohen's *d*, Pearson's *r*), indicating how they were calculated

Our web collection on statistics for biologists contains articles on many of the points above.

Software and code

Policy information about availability of computer code

Data collection	All molecular dynamic simulations were performed using GROMACS version 2022.3 (https://manual.gromacs.org/2022.3 /install-guide/ index.html)
Data analysis	VMD version 1.9.3 available at https://www.ks.uiuc.edu/Research/vmd/.<br>UCSF ChimeraX version 1.7 available at https://www.cgl.ucsf.edu/chimerax/.<br>3DNA version 2.4 available at https://x3dna.org.<br>The supplementary scripts in this study is available from the Figshare repository https://doi.org/10.6084/m9.figshare.33271119

For manuscripts utilizing custom algorithms or software that are central to the research but not yet described in published literature, software must be made available to editors and reviewers. We strongly encourage code deposition in a community repository (e.g. GitHub). See the Nature Portfolio guidelines for submitting code & software for further information.

Data

Policy information about availability of data

All manuscripts must include a data availability statement. This statement should provide the following information, where applicable:

- Accession codes, unique identifiers, or web links for publicly available datasets
- A description of any restrictions on data availability
- For clinical datasets or third party data, please ensure that the statement adheres to our policy

The molecular dynamics simulation trajectory data, initial coordinates and final coordinates are provided in the Figshare repository at https://doi.org/10.6084/m9.figshare.32145079 and https://doi.org/10.6084/m9.figshare.32372565. The cryo-ET maps used in this study are publicly available through the Dryad repository https://datadryad.org/dataset/doi:10.5061/dryad.ttdz08m21. Nucleosome structures used in this study include PDB accession codes 6L4A [https://doi.org/10.2210/pdb6L4A/pdb] and 1KX5 [https://doi.org/10.2210/pdb1KX5/pdb]. The chromatin samples and vitrification conditions are deposited in the Figshare repository at https://doi.org/10.6084/m9.figshare.33246018. The chromatin raw tilt series are available from Dryad data depository (https://datadryad.org/dataset/doi:10.5061/dryad.ttdz08m21). The library of fitted conformations generated in this study have been deposited in the Figshare repository at https://doi.org/10.6084/m9.figshare.32399967.

Research involving human participants, their data, or biological material

Policy information about studies with human participants or human data. See also policy information about sex, gender (identity/presentation), and sexual orientation and race, ethnicity and racism.

Reporting on sex and gender	Not applicable, this study did not involve human participants, human data, or human materials.
Reporting on race, ethnicity, or other socially relevant groupings	*Please specify the socially constructed or socially relevant categorization variable(s) used in your manuscript and explain why they were used. Please note that such variables should not be used as proxies for other socially constructed/relevant variables (for example, race or ethnicity should not be used as a proxy for socioeconomic status).*<br>*Provide clear definitions of the relevant terms used, how they were provided (by the participants/respondents, the researchers, or third parties), and the method(s) used to classify people into the different categories (e.g. self-report, census or administrative data, social media data, etc.)*<br>*Please provide details about how you controlled for confounding variables in your analyses.*
Population characteristics	*Describe the covariate-relevant population characteristics of the human research participants (e.g. age, genotypic information, past and current diagnosis and treatment categories). If you filled out the behavioural & social sciences study design questions and have nothing to add here, write "See above."*
Recruitment	*Describe how participants were recruited. Outline any potential self-selection bias or other biases that may be present and how these are likely to impact results.*
Ethics oversight	*Identify the organization(s) that approved the study protocol.*

Note that full information on the approval of the study protocol must also be provided in the manuscript.

Field-specific reporting

Please select the one below that is the best fit for your research. If you are not sure, read the appropriate sections before making your selection.

☒ Life sciences ☐ Behavioural & social sciences ☐ Ecological, evolutionary & environmental sciences

For a reference copy of the document with all sections, see nature.com/documents/nr-reporting-summary-flat.pdf

Life sciences study design

All studies must disclose on these points even when the disclosure is negative.

Sample size	We performed simulations for four systems, with three independent replicates for each system to ensure statistical robustness. Each simulation replicate comprised 40,000 frames spanning 4 µs. The MD simulation conformations were compared with 20 experimental Cryo-ET density maps (7 maps for chromatin array vitrified at 0 mM Mg2+, 13 maps vitrified at 0.75 mM Mg2+).
Data exclusions	No data was excluded.
Replication	For each nucleosome system, we performed three independent simulation runs. All simulation runs are successfully performed and the calculated measurements vary little among different independent runs.
Randomization	Randomization is not applicable. The entire frames from each simulation trajectory were used for our data analysis.
Blinding	Blinding is not applicable. We do not collect any statistical samples in our simulation studies as all our judgments are based on the convergence of the simulation runs and variances of measurements among different runs. The data from the simulation trajectories were directly used for analysis.

Reporting for specific materials, systems and methods

We require information from authors about some types of materials, experimental systems and methods used in many studies. Here, indicate whether each material, system or method listed is relevant to your study. If you are not sure if a list item applies to your research, read the appropriate section before selecting a response.

Materials & experimental systems

n/a	Involved in the study
☒	☐ Antibodies
☒	☐ Eukaryotic cell lines
☒	☐ Palaeontology and archaeology
☒	☐ Animals and other organisms
☒	☐ Clinical data
☒	☐ Dual use research of concern
☒	☐ Plants

Methods

n/a	Involved in the study
☒	☐ ChIP-seq
☒	☐ Flow cytometry
☒	☐ MRI-based neuroimaging

Plants

Seed stocks	Not applicable because the study did not involve any plant materials.
Novel plant genotypes	Not applicable because the study did not involve any plant materials.
Authentication	Not applicable because the study did not involve any plant materials.
