# Supplementary material for: Molecular dynamics guided all-atom reconstruction of cryo-ET maps reveals mechanisms of histone tail mediated chromatin compaction
Source: Nat Commun. 2026 Sep 15;17:10811. doi: 10.1038/s41467-026-77871-9 (PMC13669341; doi:10.1038/s41467-026-77871-9)
Supplement: Supplementary file 4 [file 41467_2026_77871_MOESM5_ESM.pdf]

nature portfolio

Peer Review File

Molecular Dynamics Guided All-Atom Reconstruction of Cryo-ET Maps Reveals Mechanisms of Histone Tail Mediated Chromatin Compaction

Corresponding Author: Professor Anna Panchenko

This file contains all reviewer reports in order by version, followed by all author rebuttals in order by version.

Version 0:

Reviewer comments:

Reviewer #1

(Remarks to the Author)
This study combined all-atom molecular dynamics (MD) simulations of tri-nucleosomes with Cryo-ET maps to provide a new structural framework for investigating histone tail-mediated chromatin folding. There are two major findings. 1) The study clarified that compaction of tri-nucleosomes is driven by three major interactions of histone tails: histone tail-DNA interactions and inter-nucleosomal H2A-H4 and H3-H4 tail-tail interactions, which provide atomistic insights into chromatin structure regulation. 2) The cryo-ET density with Mg^{2+} fits better with tri-nucleosome with histone-tails than tailless ones, while cryo-ET density without Mg^{2+} fits better with tailless tri-nucleosomes, as in Figure S4-S7.
Technically, the MD simulations and the fitting to cryo-ET density are performed properly with well-tested methods and thus are reliable, I consider. The study provides new insights on the role of individual histone tails in chromatin compaction and thus is valuable.
However, I have some concerns about the limitations of the results and clarity of the presentation, which should be addressed.
1) The linker DNA length dependence. As is well-known, folding of nucleosome-arrays strongly depends on the linker-length, partly due to the rotation phase and the degree of disorder. While histone-tail interactions should depend on linker DNA length, the current work uses only one linker DNA length (22 bp). Notably, even if nucleosomal DNA partially unwraps in the simulation, altered linker DNA does not change the rotation phase.
2) Interpretation of Figures 2a and c is unclear to me. While, I think, it is the fitting of tri-nucleosome models onto cryo-ET maps, the fitted models do not show linker DNAs. I thought the inference of linker DNA is one of the most interesting outcomes in this fitting. Can't authors add linker DNAs on the model?
In addition, what is the meaning of colors in the fitted nucleosomes? Does it have some meaning?
3) In Figures S4-S7, the differences in the distributions of correlation and the inside-the-contour values, which is the core results for the second point of finding, are rather small. I was not sure if it is statistically significant or not. Authors should provide some statistical analysis on the difference.

Reviewer #2

(Remarks to the Author)
Manuscript, "Molecular Dynamics-Guided All-Atom Reconstruction of Cryo-ET Maps Reveals Mechanisms of Histone Tail-Mediated Chromatin Compaction" by Shuxiang Li, Maria J. Aristizabal, Sergei A. Grigoryev, and Anna R. Panchenko, is an original study of nucleosome dynamics in the context of chromatin fiber. The object of the study is the tri-nucleosome model in atomistic representation, which represents a minimal chromatin model (because a central nucleosome interacts with neighboring nucleosomes on the proximal and distal ends, and at the same time this model allows to measure some parameters between non-directly connected nucleosomes). The main method of the study is all-atom MD simulation, which is computationally expensive and absolutely necessary for the study, as it aims to establish molecular mechanisms of internucleosome interactions. The tri-nucleosome is a large system for the method, especially with the 4-point water model that the authors used here, and the collected simulations took a lot of computational time.
Such simulations provided two sets of significant results. First, the authors suggested an application of observed conformational ensemble for fitting cryo-EM and cryo-ET maps and demonstrated an advantage of such an approach with

respect to the most used one (fitting of mono-nucleosome X-ray structures). On the other hand, atomistic details allowed the authors to determine new histone-histone and histone-DNA interactions between nucleosomes in the context of the chromatin fiber.
These data have a high level of scientific novelty and the quality of the text is also worth mentioning. The presentation of the material is consistent and logically connected. However, before the article can be considered for publication several points have to be addressed

1. A couple of comments on residue-residue contacts. P.7 “The vast majority of these interactions were mediated by histone tails” – it is not clear if the authors measured contacts of a certain tail with another tail of another nucleosome as a whole? It would be helpful to get in the Supplement the average numbers of different contact types, e.g., fractions of tail-tail contacts, tail-globular domain contacts, and tail-DNA contacts. And a related question: Figure S15 (“The average number of histone-histone interactions between Nuc1 and Nuc3 in NUCtail.”) - does the biggest bar (H4 with H2A) refer to the most studied contact between the H4 tail and H2A acidic patch residues or not? If so, it may be worth mentioning in the discussion or results section.
2. There is a beautiful agreement between MD and experiment with regard to inter-nucleosome distances. But in MDS with tails and without Mg2+, the disjointed nucleosome median distance was lower than the experimental median value with Mg2+ (Figure 2d). Does it mean that MD force fields overestimate the strength of the tail's contacts with the neighboring nucleosome? Can the authors provide some discussion on MD force field limitations to this end?
3. I would recommend expanding discussion. To me, it would be worth discussing alternative approaches of molecular modeling in the application of cryo-EM maps fitting. For example, the authors emphasize the importance of consideration of linker DNA flexibility when building models. Can coarse-grained methods provide such reliable information for the task, and maybe higher computational speed would even give some advantage? Also, the authors may suggest some improvement of the inter-nucleosome interactions description in mesoscale modeling?
4. https://doi.org/10.1016/j.bpj.2019.12.010 https://pubs.acs.org/doi/10.1021/acs.jpcb.9b07898 Here are two more MD studies of inter-nucleosome interactions that may be further discussed. For example, the latter implies that H3 and H2B may form contacts.
5. P. 7 “Specifically, salt bridge formation was observed between positively charged residue K9 of the H3 tail and negatively charged residue D24 of the H4 tail.”, “Additional residues involved in H3–H4 tail-tail interactions included T6, R8, and T11 from H3, and L22, Q27, and T30 from H4, with contact frequencies ranging from 10% to 45%.” - not sure that it is correct to define that as tail-tail interaction because D24, Q27, and T30 of H4 are related to the alpha1-ext helix of H4.
6. Introduction: “Chromatin, the complex of DNA and histone proteins,...” – According to the classic definition, chromatin also includes non-histone proteins and RNA. Please paraphrase it.
7. Results, P. 9: “However, it should be mentioned that variant nucleosomes containing H2A.Z C-terminal tails have a different sequence and structure, which may extend to the neighboring nucleosomes and form interactions with their acidic patch and mediate higher-order chromatin compaction, as was suggested previously 39,40 264.” – I suggest that this should go to the Discussion section.
8. The first appearing figures are S4c, S5c; please check the numeration.
9. “electron microscopy (EM)” abbreviation first was introduced on p.10
10. Please provide a link to raw data (initial models and trajectories).

Reviewer #3

(Remarks to the Author)
This is a very interesting and ambitious study that addresses an extremely important problem in chromatin biology: how to obtain physically meaningful atomistic models of native chromatin organization and how simulations can be used to complement cryo-ET experiments. The authors propose a conceptually strong method, namely using atomistic MD simulations of tri-nucleosomes to build a database of templates to resolve cryo-ET density maps. The overall idea is strong, timely, and well aligned with similar current efforts to integrate cryo-ET experiments and simulations to solve chromatin structure in condensed conditions (i.e., Kreysing et al bioRxiv 2025 and Zhou et al Science 2025). While I think the work is important, I have some concerns, which I list below.

1. The cryo-ET data correspond to native chromatin, in which linker DNA lengths are inherently heterogeneous. In contrast, the tri-nucleosome simulations are constructed using a single, fixed linker length of 22 bp, with any apparent variability arising from DNA unwrapping only in the tailless systems rather than from explicit linker-length diversity. The tailless simulations are used only for open chromatin, meaning that for condensed chromatin only simulations with fixed linker lengths are used. Chromatin structure is known to be extremely sensitive to linker DNA length, particularly under condensed conditions, as has been convincingly demonstrated in recent studies combining cryo-ET with coarse-grained simulations of chromatin condensates (e.g., Zhou et al., Science, 2025). Without explicitly sampling tri-nucleosomes spanning a broad range of linker lengths, it is difficult to justify the claim that the simulated tri-nucleosome ensemble is representative of the experimental chromatin structures.

Consequently, in its present form the study does not provide a general solution to the problem it sets out to address, but rather illustrates a limited proof of concept. While the conceptual idea is strong, the work does not provide a solution to overcome the fundamental limitation that atomistic MD simulations of tri-nucleosomes are severely constrained by achievable configurational sampling. As a result, the analysis is restricted to structures derived from only two specific tri-nucleosome systems, which is insufficient to capture the structural heterogeneity of native chromatin. To meaningfully resolve native chromatin organization, the approach would require systematic MD simulations of a much wider range of tri-nucleosome geometries with varying linker DNA lengths. This major limitation must be explicitly acknowledged, and its

consequences for the interpretation and generality of the cryo-ET fitting should be discussed in a much more critical and transparent manner.

2. On a related note, each atomistic trajectory is only 3 µs long, which is extremely short for a system of this size and complexity. For comparison, coarse-grained simulations of tri-nucleosomes using models such as AICG2+/3SPN.2, where dynamics are orders of magnitude faster, require on the order of 0.1 ms per trajectory to achieve sufficient sampling (Kenzaki et al J Mol Biol 2021). The authors should provide a clear justification for why 3 µs of atomistic sampling is sufficient, or explicitly acknowledge that sampling is incomplete and the implications.

3. Nucleosomes lacking histone tails are expected to be significantly less stable, as histone tails contribute strongly to DNA wrapping. The simulations indeed show extensive DNA unwrapping and effectively variable linker lengths in the tailless systems. However, the conformations of tri-nucleosomes without tails are then used to interpret cryo-ET maps of native chromatin at 0 mM Mg^{2+}. This raises an important conceptual inconsistency: at low counterion concentration, electrostatic interactions between DNA and histones are strong, making extensive DNA unwrapping at such solution conditions unlikely. In other words, the MD simulations appear to represent partially unwrapped nucleosomes, whereas the cryo-ET data at 0 mM Mg^{2+} should correspond to that of fully wrapped nucleosomes, making the tailless fitting units intrinsically inaccurate for the 0 mM Mg^{2+} condition. Are the authors proposing that DNA unwrapping is genuinely present under these experimental conditions, or is this an artefact of the model? If the latter, how does it affect the validity of the fitting procedure?

4. The goodness of fit between MD-generated tri-nucleosome conformations and cryo-ET maps is evaluated using center-to-center nucleosome distances. While this metric is convenient, it is a low resolution metric and does not capture key aspects of chromatin organization. In particular, relative nucleosome orientation is critical for chromatin structure. Because this distance-based metric is central to the authors' claim that the MD-derived models reproduce experimental chromatin organization, a much stronger justification is required. The authors should explain why center-to-center distances alone are sufficient, or provide additional orientational or angular metrics that can more strictly compare the simulated vs cryoET data.

5. The analysis of inter-nucleosomal interactions mediated by histone tails is one of the most interesting aspects of the paper. However, I found it difficult to follow how the conclusions are supported by the figures. For example, the manuscript states that H3 and H4 tails dominate inter-nucleosomal DNA interactions, whereas H2A and H2B tails play a minor role. In Figures 5 and S19, the mean number of contacts is shown as a function of SHL, but it is not obvious how the dominance of H3/H4 over H2A/H2B follows from these plots. If one were to integrate the contact profiles, the total number of contacts might appear comparable across tail types. The authors should clarify how these conclusions are quantitatively derived from the data and guide the reader more explicitly through the figures.

6. The manuscript concludes that H2A and H2B tails play a relatively minor role in chromatin compaction. However, the analysis is based on the number and spatial distribution of contacts, not on energetic contributions. The number of contacts alone is not a direct measure of enthalpic stabilization. This distinction should be acknowledged more clearly, and the conclusions should be phrased more cautiously unless energetic analyses are provided.

Reviewer #4

(Remarks to the Author)
The manuscript describes a method for interpreting chromatin structure based on fitting of tri-nucleosome templates obtained from molecular dynamics simulations into cryo-electron tomographic data. The use of atomic models allows, in principle, addressing histone tail based interactions, and the authors claim that such a level of interpretation can be achieved with their method.

Major comments

While the proposed approach might be conceptually interesting and has potential to capture heterogeneity and complexity of dynamic chromatin fibrils, the manuscript at its present state does not provide sufficient evidence demonstrating effectiveness of the method at the fitting stage. Nucleosome fitting precision and robustness, as well as positioning of linker DNA, need to be thoroughly explored and justified before addressing applicability of the method to atomic scale interpretations. High general cross-correlation values alone are not sufficient and may indicate overfitting. An initial assessment could be performed, for example, by extracting subtomograms of nucleosomes, applying the rotations obtained from the model fitting, and summing the particles. The resulting average and its further classification would provide an indication of the fitting quality.

Fitting of both tri- and mono- nucleosome templates into tomographic data should be performed taking into account inherent limitations of cryo-ET data, such contrast transfer function, missing wedge, and electron dose accumulation. Additionally, fitting should be performed on raw data without using nonlinear filtering approaches which are prone to hallucinations.

MD simulations performed by the authors were initialized from a single tri-nucleosome template. Even considering partial unwrapping of DNA, it is difficult to imagine that this system sufficiently represents complexity of long native isolated chromatin fibers, with variable linker lengths and potential presence of linker histones or other binding factors. The use of such a complex system for initial proof of principle study should be better justified. Initial benchmarking of the concept would be more robust on simpler systems as reconstituted chromatin fibers, ideally tri-nucleosome assemblies.

Minor comments

Introduction, lines 65 to 70
Recent studies have moved beyond linking nucleosomes by straight DNA linkers (https://www.biorxiv.org/content/10.1101/2025.04.09.647790v1; https://www.biorxiv.org/content/10.1101/2022.08.16.502515v2). It is also not clear what the authors mean by "spatial coherence".

Results, lines 90 to 107
This section mixes presentation of results with extended introduction and partially repeats content already described in the lines 65 to 70.

Results, lines 108 to 120 and Figure 1
Figure 1 is confusing and not sufficient to illustrate the advantages of tri-nucleosome fitting. In Figure 1b, fitting of nucleosomes appears less reliable (in particular for nucleosome N1) compared to the case of single nucleosome template fitting (Figure 1a). Figures 1c and 1d show densities whose shapes are difficult to correlate with nucleosome structures.

Discussion, lines 275 to 280. This part closely resembles the introduction and does not provide additional discussion or new meaning.

Version 1:

Reviewer comments:

Reviewer #1

(Remarks to the Author)
The authors largely revised the manuscript according to my comments. Especially, additional MD simulations with 2 more lengths of linker DNA broadened the conformational space of tri-nucleosomes marekedly. The resulting shows better fitting than the previous one.
I consider the revised manuscript can be published as is.

Reviewer #2

(Remarks to the Author)
I have carefully reviewed the authors' responses to my previous comments as well as the revised manuscript. I find that the authors have addressed all of my concerns thoroughly and satisfactorily. Given the complete readiness of the manuscript, the high quality of the research, and its undeniable relevance, I recommend the manuscript for publication in its current form.

Reviewer #3

(Remarks to the Author)
The authors have done an excellent job of addressing all my concerns, and the revised manuscript is excellent. I am very supportive of this revised version being published as it is.

Reviewer #4

(Remarks to the Author)
I thank the authors for addressing my specific comments. However, regarding their response to my major point, in my opinion, additional validation is still required.

The current best practices in cryo-ET data processing should not be overlooked. Cryo-ET has several well-known limitations, particularly high noise levels and the missing wedge (MW).
Cross-correlation, which is used in this manuscript as the fitting metric, is calculated on the basis on pixel intensity values. The application of non-linear filters, such as NAD or deep-learning-based networks, modifies pixel values while enhancing contrast in a way that cannot be fully predicted and taken into an account. As a result, such processing may artificially increase the cross-correlation score and thereby influence the fitting outcome. The previous publication referenced in the rebuttal https://doi.org/10.1093/nar/gkaf457 (Supplementary Figures S1BC) compares two nonlinear filters with each other, but not with raw data or with raw data with applied standard linear filters, such as a Fourier band-pass filter, which is standard in cryo-ET data processing.

The missing wedge has the most severe impact, resulting in artificial elongation of structures along the electron beam axis and, consequently, prone of an artificial increase in the volume of particles making fitting more permissive and less precise.

Application of non-linear filters to increase the contrast may further enhance this effect.

The Chimera cross-correlation-based fitting would only be justifiable if the EM data have a uniform resolution and without non-linear filters applied, for example SPA maps or sub-tomogram averages. In case of raw cryo-ET data the quantitative assessment of the fitting accuracy in relation to missing wedge and contrast enhancement should be provided.

This evaluation does not necessarily require recalculating the fitting for all MD frames, which I understand would be computationally heavy. It could be initially performed on the obtained best-fits and include two components:
(i) assessment of the influence of the missing wedge and the application of nonlinear denoising filters on the position and orientation of the fitted tri-nucleosome template.
(ii) determination the translational and angular differences between nucleosome positions and orientations obtained by mono-nucleosome template fitting (with MW taken into account and without non-linear contrasting) and the positions and orientations of the same nucleosomes resulted from tri-nucleosome template fitting.

To help readers to be aware of the impact of the missing wedge, the Figures showing representative fitting results for mono- and tri-nucleosomes should also include XZ views of the cryo-ET densities.

Version 2:

Reviewer comments:

Reviewer #4

(Remarks to the Author)
The authors have adequately addressed all of the comments I raised. I recommend publication of the manuscript as it is.

Open Access This Peer Review File is licensed under a Creative Commons Attribution 4.0 International License, which permits use, sharing, adaptation, distribution and reproduction in any medium or format, as long as you give appropriate credit to the original author(s) and the source, provide a link to the Creative Commons license, and indicate if changes were made.
In cases where reviewers are anonymous, credit should be given to 'Anonymous Referee' and the source.
The images or other third party material in this Peer Review File are included in the article's Creative Commons license, unless indicated otherwise in a credit line to the material. If material is not included in the article's Creative Commons license and your intended use is not permitted by statutory regulation or exceeds the permitted use, you will need to obtain permission directly from the copyright holder.
To view a copy of this license, visit https://creativecommons.org/licenses/by/4.0/

RESPONSE TO REVIEWERS' COMMENTS

We sincerely thank the reviewers for their thoughtful and constructive comments. These suggestions have helped us improve the quality of our work. In response to these comments, we performed additional all-atom MD simulations with different linker DNA lengths and added new analyses and clarifications throughout the revised manuscript and Supplementary Information. All changes in the manuscript are highlighted in red.

Responses to reviewer #1

Overall comment: This study combined all-atom molecular dynamics (MD) simulations of tri-nucleosomes with Cryo-ET maps to provide a new structural framework for investigating histone tail-mediated chromatin folding. There are two major findings. 1) The study clarified that compaction of tri-nucleosomes is driven by three major interactions of histone tails: histone tail-DNA interactions and inter-nucleosomal H2A-H4 and H3-H4 tail-tail interactions, which provide atomistic insights into chromatin structure regulation. 2) The cryo-ET density with Mg2+ fits better with tri-nucleosome with histone-tails than tailless ones, while cryo-ET density without Mg2+ fits better with tailless tri-nucleosomes, as in Figure S4-S7.

Technically, the MD simulations and the fitting to cryo-ET density are performed properly with well-tested methods and thus are reliable, I consider. The study provides new insights on the role of individual histone tails in chromatin compaction and thus is valuable.

Response: We thank the reviewer for the concise summary of our work and for the encouraging comments on our work. We have revised the manuscript further in response to the points raised below.

Comment 1: The linker DNA length dependence. As is well-known, folding of nucleosome-arrays strongly depends on the linker-length, partly due to the rotation phase and the degree of disorder. While histone-tail interactions should depend on linker DNA length, the current work uses only one linker DNA length (22 bp). Notably, even if nucleosomal DNA partially unwraps in the simulation, altered linker DNA does not change the rotation phase.

Response: We thank the reviewer for raising this important point. We fully agree that linker length is one of the determinants of chromatin fiber folding and can modulate histone-tail interactions. To address this concern, in addition to the original 22 bp system described in the manuscript, we constructed tri-nucleosome systems with 26 bp and 31 bp linker DNA with histone tails (Supplementary Figures S22 and S23) and performed additional all-atom MD simulations for 4 μs, three independent runs for each system. In addition, we have extended the simulation time for our original 22 bp linker system, up to 4 μs. Our new results show that tri-nucleosome conformations depend on the linker lengths (Supplementary Figure S10). Indeed, the native chromatin *in situ* experiments contains various linker lengths and disk-to-disk orientations. Our new results show that the distributions of distances and dihedral angles, which include all conformations with various linker lengths, become considerably wider, better recapitulating the behaviour of native chromatin (Figure 2f-h). We have incorporated these new analyses into the revised manuscript.

Comment 2: Interpretation of Figures 2a and c is unclear to me. While, I think, it is the fitting of tri-nucleosome models onto cryo-ET maps, the fitted models do not show linker DNAs. I thought the inference of linker DNA is one of the most interesting outcomes in this fitting. Can't authors add linker DNAs on the model?

In addition, what is the meaning of colors in the fitted nucleosomes? Does it have some meaning?

Response: We appreciate the reviewer's comment. We should clarify that Figures 2a and 2c (2e in the revised manuscript) are based on mono-nucleosome rigid-body fitting into the Cryo-ET density maps for the purpose of computing the disjointed and consecutive nucleosome center-to-center distances from experimental native chromatins. This is the way the original Cryo-ET study interpreted and calculated the inter-nucleosomal distances. The distributions of these distances were then compared with the corresponding distributions derived from the tri-nucleosome MD simulations (Figures 2b and 2f). Because these panels are based on mono-nucleosome fitting, the linker DNA is not shown. We have clarified this in the revised manuscript. We have also clarified in the figure legend that the colors assigned to the fitted nucleosomes in these panels do not encode any structural or functional property and were used only for ease of visualizing individual nucleosomes in different density regions. On the other hand, fitting which includes DNA linkers is shown in Figure 1. In addition, we show in Supplementary Figure S4 that fitting of tri-

nucleosome systems into the Cryo-ET maps (including linkers) produces better goodness-of-fit values compared to the mono-nucleosome fitting.

Comment 3: In Figures S4-S7, the differences in the distributions of correlation and the inside-the-contour values, which is the core results for the second point of finding, are rather small. I was not sure if it is statistically significant or not. Authors should provide some statistical analysis on the difference.

Response: Following the reviewer's suggestions, we have added statistical analysis on differences in the distributions of correlation and the inside-the-contour values in Figures S4-S7 (combined and renumbered as Figures S4–S5 in the revised manuscript).

Responses to reviewer #2

Overall comment: Manuscript, "Molecular Dynamics-Guided All-Atom Reconstruction of Cryo-ET Maps Reveals Mechanisms of Histone Tail-Mediated Chromatin Compaction" by Shuxiang Li, Maria J. Aristizabal, Sergei A. Grigoryev, and Anna R. Panchenko, is an original study of nucleosome dynamics in the context of chromatin fiber. The object of the study is the tri-nucleosome model in atomistic representation, which represents a minimal chromatin model (because a central nucleosome interacts with neighboring nucleosomes on the proximal and distal ends, and at the same time this model allows to measure some parameters between non-directly connected nucleosomes). The main method of the study is all-atom MD simulation, which is computationally expensive and absolutely necessary for the study, as it aims to establish molecular mechanisms of internucleosome interactions. The tri-nucleosome is a large system for the method, especially with the 4-point water model that the authors used here, and the collected simulations took a lot of computational time.

Such simulations provided two sets of significant results. First, the authors suggested an application of observed conformational ensemble for fitting cryo-EM and cryo-ET maps and demonstrated an advantage of such an approach with respect to the most used one (fitting of mono-nucleosome X-ray structures). On the other hand, atomistic details allowed the authors to determine new histone-histone and histone-DNA interactions between nucleosomes in the context of the chromatin fiber.

These data have a high level of scientific novelty and the quality of the text is also worth mentioning. The presentation of the material is consistent and logically connected. However, before the article can be considered for publication several points have to be addressed.

Response: We sincerely thank the reviewer for thoughtful comments on our study.

Comment 1: A couple of comments on residue-residue contacts. P.7 "The vast majority of these interactions were mediated by histone tails" – it is not clear if the authors measured contacts of a certain tail with another tail of another nucleosome as a whole? It would be helpful to get in the Supplement the average numbers of different contact types, e.g., fractions of tail-tail contacts, tail-globular domain contacts, and tail-DNA contacts. And a related question: Figure S15 ("The average number of histone-histone interactions between Nuc1 and Nuc3 in NUCtail.") - does the biggest bar (H4 with H2A) refer to the most studied contact between the H4 tail and H2A acidic patch residues or not? If so, it may be worth mentioning in the discussion or results section.

Response: Per reviewer's suggestion, we have added new figures that quantify the average numbers of different contact types, including inter-nucleosomal histone tail-tail contacts (Supplementary Figure S14) and intra- and inter-nucleosomal histone tail-DNA contacts (Figures 5a, 5b and Supplementary Figure S15) in the revised manuscript. We also revised the corresponding text to clarify that the inter-nucleosomal histone tail-tail contacts were measured between specific histone tails from different nucleosomes. As for the H4–H2A tail contacts, our analysis shows that most of these contacts involve the H4 N-terminal tail and the H2A C-terminal tail. We did not observe significant interactions between the H4 tail and the H2A acidic patch residues.

Comment 2: There is a beautiful agreement between MD and experiment with regard to inter-nucleosome distances. But in MDS with tails and without Mg2+, the disjointed nucleosome median distance was lower than the experimental median value with Mg2+ (Figure 2d). Does it mean that MD force fields overestimate the strength of the tail's contacts with the neighboring nucleosome? Can the authors provide some discussion on MD force field limitations to this end?

Response: We think the difference between the simulated (tri-nucleosome with tails) and experimental (with Mg^{2+}) disjointed nucleosome distances may reflect a combination of several factors, including limited conformational sampling, the use of a fixed linker DNA length and

possible force-field limitations in describing tail-mediated interactions in a highly crowded chromatin environment. We note that the experimental Cryo-ET maps of native chromatin contain heterogeneous linker lengths, whereas our original NUC_{tail} simulations used only a 22 bp linker. To address this issue, we performed additional all-atom MD simulations with 26 bp and 31 bp linkers (Supplementary Figures S22 and S23). Our new results show that the distributions of disjointed distances, which include all conformations with various linker lengths, become considerably wider and shift to the higher values, better recapitulating the behaviour of the native chromatin (Figure 2f-h). We also acknowledge that force-field accuracy and accessible simulation timescales remain general limitations of current all-atom chromatin simulations. We have revised the manuscript and explicitly mentioned these limitations.

Comment 3: I would recommend expanding discussion. To me, it would be worth discussing alternative approaches of molecular modeling in the application of cryo-EM maps fitting. For example, the authors emphasize the importance of consideration of linker DNA flexibility when building models. Can coarse-grained methods provide such reliable information for the task, and maybe higher computational speed would even give some advantage? Also, the authors may suggest some improvement of the inter-nucleosome interactions description in mesoscale modeling?

Response: We thank the reviewer for this constructive suggestion. We have expanded the Discussion accordingly.

Comment 4: https://doi.org/10.1016/j.bpj.2019.12.010; https://pubs.acs.org/doi/10.1021/acs.jpcb.9b07898 Here are two more MD studies of inter-nucleosome interactions that may be further discussed. For example, the latter implies that H3 and H2B may form contacts.

Response: We thank the reviewer for mentioning these relevant studies. We have incorporated them into the revised manuscript.

Comment 5: P. 7 “Specifically, salt bridge formation was observed between positively charged residue K9 of the H3 tail and negatively charged residue D24 of the H4 tail.”, “Additional residues

involved in H3–H4 tail-tail interactions included T6, R8, and T11 from H3, and L22, Q27, and T30 from H4, with contact frequencies ranging from 10% to 45%." - not sure that it is correct to define that as tail-tail interaction because D24, Q27, and T30 of H4 are related to the alpha1-ext helix of H4.

Response: We thank the reviewer for this important clarification. In the revised manuscript, we have refined the terminology and updated the residue description to distinguish tail–tail contacts from tail–core (or tail–α1-extension) contacts.

Comment 6: Introduction: "Chromatin, the complex of DNA and histone proteins,..." – According to the classic definition, chromatin also includes non-histone proteins and RNA. Please paraphrase it.

Response: Per reviewer's suggestion, we have revised the sentence accordingly.

Comment 7: Results, P. 9: "However, it should be mentioned that variant nucleosomes containing H2A.Z C-terminal tails have a different sequence and structure, which may extend to the neighboring nucleosomes and form interactions with their acidic patch and mediate higher-order chromatin compaction, as was suggested previously 39,40 264." – I suggest that this should go to the Discussion section.

Response: Per reviewers' suggestion, we have moved this text from the Results section to the Discussion.

Comment 8: The first appearing figures are S4c, S5c; please check the numeration.

Response: We thank the reviewer for noticing this issue and have corrected the numbering and order of all Supplementary Figures in the revised manuscript.

Comment 9: "electron microscopy (EM)" abbreviation first was introduced on p.10.

Response: We have updated this abbreviation on p.10.

Comment 10: Please provide a link to raw data (initial models and trajectories).

Response: We have added the link to the deposited raw data in the revised manuscript.

Responses to reviewer #3

Overall comment: This is a very interesting and ambitious study that addresses an extremely important problem in chromatin biology: how to obtain physically meaningful atomistic models of native chromatin organization and how simulations can be used to complement cryo-ET experiments. The authors propose a conceptually strong method, namely using atomistic MD simulations of tri-nucleosomes to build a database of templates to resolve cryo-ET density maps. The overall idea is strong, timely, and well aligned with similar current efforts to integrate cryo-ET experiments and simulations to solve chromatin structure in condensed conditions (i.e., Kreysing et al bioRxiv 2025 and Zhou et al Science 2025). While I think the work is important, I have some concerns, which I list below.

Response: We sincerely thank the reviewer for the careful and thoughtful evaluation of our work and for pointing out this new interesting study which we cited in the revised manuscript.

Comment 1: The cryo-ET data correspond to native chromatin, in which linker DNA lengths are inherently heterogeneous. In contrast, the tri-nucleosome simulations are constructed using a single, fixed linker length of 22 bp, with any apparent variability arising from DNA unwrapping only in the tailless systems rather than from explicit linker-length diversity. The tailless simulations are used only for open chromatin, meaning that for condensed chromatin only simulations with fixed linker lengths are used. Chromatin structure is known to be extremely sensitive to linker DNA length, particularly under condensed conditions, as has been convincingly demonstrated in recent studies combining cryo-ET with coarse-grained simulations of chromatin condensates (e.g., Zhou et al., Science, 2025). Without explicitly sampling tri-nucleosomes spanning a broad range of linker lengths, it is difficult to justify the claim that the simulated tri-nucleosome ensemble is representative of the experimental chromatin structures.

Consequently, in its present form the study does not provide a general solution to the problem it sets out to address, but rather illustrates a limited proof of concept. While the conceptual idea is strong, the work does not provide a solution to overcome the fundamental limitation that atomistic

MD simulations of tri-nucleosomes are severely constrained by achievable configurational sampling. As a result, the analysis is restricted to structures derived from only two specific tri-nucleosome systems, which is insufficient to capture the structural heterogeneity of native chromatin. To meaningfully resolve native chromatin organization, the approach would require systematic MD simulations of a much wider range of tri-nucleosome geometries with varying linker DNA lengths. This major limitation must be explicitly acknowledged, and its consequences for the interpretation and generality of the cryo-ET fitting should be discussed in a much more critical and transparent manner.

Response: We thank the reviewer for pointing out the limitations of the original manuscript imposed by the fixed linker DNA length sampling of all-atom MD simulations. We acknowledge that the tri-nucleosome conformations from 22 bp linker DNA simulations are not sufficient to capture all the structural heterogeneity of native chromatin. In response, we utilized all available computational resources to construct and perform additional all-atom MD simulations of tri-nucleosome systems with 26 bp and 31 bp linker DNA for 4 μs (Supplementary Figures S22 and S23) based on representative folding conformations of tri-nucleosome with different linker lengths [1], in addition to the original 22 bp system. In addition, we have extended the simulation time for our original 22 bp linker system, up to 4 μs. The results from these new simulations show clearly that linker length strongly affects tri-nucleosome conformations (Supplementary Figure S10). Our new results show that the distributions of disjointed distances and dihedral angles, which include all conformations with various linker lengths, become considerably wider and shift to the higher values, better recapitulating the behaviour of the native chromatin (Figure 2f-h).

We agree that the simulated ensemble remains far from exhaustive and does not capture the full structural heterogeneity of native chromatin. This is the limitation of all current chromatin modeling studies. However, we would like to point out that, even though the conformational sampling is not extensive and tail-mediated interactions may depend on the initial configurations, the sampled conformations represent physically plausible states and are likely to capture key interactions. In particular, the mere presence of histone tails between adjacent nucleosomal disks, even if exhaustive sampling of their conformational ensemble is not feasible, can locally neutralize DNA charge and modulate nucleosome–nucleosome attraction. Moreover, as we show, molecular dynamics simulations lead to a broadening of the distributions of dynamical features and improved agreement with experimental observations compared to the initial configurations. We have revised the manuscript to explicitly acknowledge these limitations.

Comment 2: On a related note, each atomistic trajectory is only 3 µs long, which is extremely short for a system of this size and complexity. For comparison, coarse-grained simulations of tri-nucleosomes using models such as AICG2+/3SPN.2, where dynamics are orders of magnitude faster, require on the order of 0.1 ms per trajectory to achieve sufficient sampling (Kenzaki et al J Mol Biol 2021). The authors should provide a clear justification for why 3 µs of atomistic sampling is sufficient, or explicitly acknowledge that sampling is incomplete and the implications.

Response: We thank the reviewer for this important point. We have extended the simulations as far as possible (currently up to 4 µs) within available computational resources. Because the NUC_{tail} system contains approximately 3.6 million atoms, including water and ions, the accessible simulation timescales for all-atom MD remain limited. We acknowledge that coarse-grained simulations can achieve more extensive conformational sampling and have discussed this in the revised manuscript. There are limitations for both all-atom and coarse-grained simulations. Whereas all-atom can explicitly model hydrogen-bonded interactions, salt-bridges, side chain rotamers, water-mediated interactions and explicit interactions with water and ions, coarse-grained simulations, indeed, can sample conformations on larger, more biologically relevant time scales. We think both types of simulations complement each other; this is a good example of a trade-off of accuracy and sampling coverage. As a reviewer mentioned, this study provides a library of tri-nucleosomal conformations, which can be expanded in the future. The atomistic level of detail is particularly important for future studies of the effects of chemical modifications, including DNA methylation and histone post-translational modifications on chromatin folding.

Comment 3: Nucleosomes lacking histone tails are expected to be significantly less stable, as histone tails contribute strongly to DNA wrapping. The simulations indeed show extensive DNA unwrapping and effectively variable linker lengths in the tailless systems. However, the conformations of tri-nucleosomes without tails are then used to interpret cryo-ET maps of native chromatin at 0 mM Mg^{2+}. This raises an important conceptual inconsistency: at low counterion concentration, electrostatic interactions between DNA and histones are strong, making extensive DNA unwrapping at such solution conditions unlikely. In other words, the MD simulations appear to represent partially unwrapped nucleosomes, whereas the cryo-ET data at 0 mM Mg^{2+} should correspond to that of fully wrapped nucleosomes, making the tailless fitting units intrinsically inaccurate for the 0 mM Mg^{2+} condition. Are the authors proposing that DNA unwrapping is

genuinely present under these experimental conditions, or is this an artefact of the model? If the latter, how does it affect the validity of the fitting procedure?

Response: We thank the reviewer for raising this important conceptual issue. The reviewer notes that DNA is expected to remain largely in a wrapped state at low counterion concentrations, whereas our $\mathrm{NUC}^{22}_{\mathrm{notail}}$ simulations exhibit extensive DNA unwrapping. First, we would like to clarify that, in addition to NaCl, other counterions are present in the experimental open chromatin cryo-ET maps. Specifically, for open chromatin, after sucrose gradient centrifugation, fractions enriched in particles with DNA sizes corresponding to 8–16 nucleosomes were collected, and dialyzed against HNE buffer (5 mM NaCl, 0.1 mM EDTA, 10 mM HEPES-NaOH, pH=7.5). Second, we would like to emphasize the timescale limitations inherent to all-atom MD simulations when modeling native chromatin conformations. Open chromatin fiber states are challenging to capture in all-atom simulations of nucleosome arrays with intact histone tails. To our knowledge, even simulations extending to ~15 μs do not show significant DNA unwrapping when histone tails are present [2]. For this reason, systems with truncated histone tails are widely employed to investigate the mechanisms of DNA unwrapping and to study chromatin unfolding [2-4].

In our work, we adopted this strategy in the $\mathrm{NUC}^{22}_{\mathrm{notail}}$ simulations to mimic open chromatin fiber conformations and showed good agreement with experimental distance distributions. This suggests that our model reproduces, at least qualitatively, the relative positioning of nucleosomal disks within the fiber. We interpret this agreement as indicating either that histone tails play a limited role in stabilizing inter-nucleosomal interactions in the open fiber (in agreement with recent work [5]), or that our explicit treatment of DNA unwrapping and effective linker lengths captures the dominant structural determinants. At present, we cannot distinguish which of these factors, or other factors, are primarily responsible for the observed agreement. We acknowledge the limitations of our approach in the Discussion section.

Comment 4: The goodness of fit between MD-generated tri-nucleosome conformations and cryo-ET maps is evaluated using center-to-center nucleosome distances. While this metric is convenient, it is a low resolution metric and does not capture key aspects of chromatin organization. In particular, relative nucleosome orientation is critical for chromatin structure. Because this distance-based metric is central to the authors' claim that the MD-derived models reproduce experimental chromatin organization, a much stronger justification is required. The

authors should explain why center-to-center distances alone are sufficient, or provide additional orientational or angular metrics that can more strictly compare the simulated vs cryoET data.

Response: We would like to clarify that we are using two ways to compare MD simulation trajectories with cryo-ET fibers. The first way is to fit the MD conformations into the cryo-ET maps. For this, we use the correlation coefficient and inside-the-contour values as goodness-of-fit measures. The second way is to compare distance distributions from simulations and experiments without any fitting involved.

Per reviewer's request, we have added additional analyses based on dihedral angles (Figures 2c, 2d, 2g and 2h). These new results have been included in the revised manuscript.

Comment 5: The analysis of inter-nucleosomal interactions mediated by histone tails is one of the most interesting aspects of the paper. However, I found it difficult to follow how the conclusions are supported by the figures. For example, the manuscript states that H3 and H4 tails dominate inter-nucleosomal DNA interactions, whereas H2A and H2B tails play a minor role. In Figures 5 and S19, the mean number of contacts is shown as a function of SHL, but it is not obvious how the dominance of H3/H4 over H2A/H2B follows from these plots. If one were to integrate the contact profiles, the total number of contacts might appear comparable across tail types. The authors should clarify how these conclusions are quantitatively derived from the data and guide the reader more explicitly through the figures.

Response: We thank the reviewer for this very helpful comment. We have added a new figure in which the contact profiles from Figures 5 and S19 are integrated to show the total numbers of inter-nucleosomal histone tail-DNA contacts formed by each histone tail type (Figure 5b, Supplementary Figure S15). The original Figures 5a, 5b and S19 are combined and renumbered as Figure S19 in the revised manuscript. This new analysis makes the comparison more direct and shows more clearly that H3 and H4 tails account for the majority of inter-nucleosomal histone tail-DNA contacts in MD simulations, whereas H2A and H2B tails contribute substantially less. We have also revised the manuscript accordingly.

Comment 6: The manuscript concludes that H2A and H2B tails play a relatively minor role in chromatin compaction. However, the analysis is based on the number and spatial distribution of contacts, not on energetic contributions. The number of contacts alone is not a direct measure of

enthalpic stabilization. This distinction should be acknowledged more clearly, and the conclusions should be phrased more cautiously unless energetic analyses are provided.

Response: We thank the reviewer for this suggestion. We have added binding free energy analyses for associations between the inter-nucleosomal histone tail-DNA interactions (Supplementary Table 1).

Responses to reviewer #4

Overall comment: The manuscript describes a method for interpreting chromatin structure based on fitting of tri-nucleosome templates obtained from molecular dynamics simulations into cryo-electron tomographic data. The use of atomic models allows, in principle, addressing histone tail based interactions, and the authors claim that such a level of interpretation can be achieved with their method.

Response: We would like to thank the reviewer for the concise summary of our work.

Major comments

While the proposed approach might be conceptually interesting and has potential to capture heterogeneity and complexity of dynamic chromatin fibrils, the manuscript at its present state does not provide sufficient evidence demonstrating effectiveness of the method at the fitting stage. Nucleosome fitting precision and robustness, as well as positioning of linker DNA, need to be thoroughly explored and justified before addressing applicability of the method to atomic scale interpretations. High general cross-correlation values alone are not sufficient and may indicate overfitting. An initial assessment could be performed, for example, by extracting subtomograms of nucleosomes, applying the rotations obtained from the model fitting, and summing the particles. The resulting average and its further classification would provide an indication of the fitting quality.

Fitting of both tri- and mono- nucleosome templates into tomographic data should be performed taking into account inherent limitations of cryo-ET data, such contrast transfer function, missing wedge, and electron dose accumulation. Additionally, fitting should be performed on raw data without using nonlinear filtering approaches which are prone to hallucinations.

MD simulations performed by the authors were initialized from a single tri-nucleosome template. Even considering partial unwrapping of DNA, it is difficult to imagine that this system sufficiently represents complexity of long native isolated chromatin fibers, with variable linker lengths and potential presence of linker histones or other binding factors. The use of such a complex system for initial proof of principle study should be better justified. Initial benchmarking of the concept would be more robust on simpler systems as reconstituted chromatin fibers, ideally tri-nucleosome assemblies.

Response: We thank the reviewer for these insightful comments. First, we would like to clarify that we are using two ways to compare MD simulation trajectories with cryo-ET fibers. The first way is to fit the MD conformations into the cryo-ET maps. For this, we use the correlation coefficient and inside-the-contour values as goodness-of-fit measures. The second way is to compare distance distributions from simulations and experiments without any fitting involved.

The fitting method and protocol used in this study were established in our previous works [6-8]. In those studies, reasonably fitting conformations were manually identified to construct models describing the internal structure of native chromatin, demonstrating the effectiveness of the approach. In our previous work, we routinely use two parallel methods for tomogram denoising and nucleome fitting. In one, we use regression denoising with AI in Dragonfly (ORS) using data synthesized by cryo-TomoSim. In the other, we used non-linear, edge-enhancing anisotropic diffusion (NAD) filtering to smooth noise and reduce highly directional diffraction effects, while minimally affecting structural resolution. This method uses structural information in the raw data to locally control the strength and direction of diffusion filtering to enhance the signal-to-noise ratio while preserving the original dimensions of the structural elements [9]. Both methods were absolutely consistent; no “hallucinations” were observed for the AI denoising, and stereological comparison showed excellent correspondence between the nonlinear anisotropic diffusion filtering and deep learning regression denoising (see Supplementary Figure S1B and 1C in the work [8]).

In the current work, we applied a similar fitting method and protocol (see Methods for detailed parameters), but used tri-nucleosome conformations derived from MD simulations instead of mono-nucleosome conformations. Due to the large number of conformations generated from MD simulations (480,000 in total), we selected 2,400 conformations for fitting into the 35 experimental cryo-ET maps, resulting in approximately 4,800,000 fitted conformations. Given the scale of this dataset, manual visualization and selection used previously are impractical. Therefore, our analysis primarily relies on ensemble-based comparisons.

We also appreciate and agree with the reviewer's point that using a single tri-nucleosome template and omitting other factors such as variable linker DNA lengths, linker histones and other binding proteins may limit the representativeness of the current ensemble. To address this, and within the constraints of available computational resources, we utilized all available computing capacity to perform additional MD simulations of tri-nucleosome systems with 26 bp and 31 bp linker DNA lengths (Supplementary Figures S22 and S23) in addition to the original 22 bp system. Our new results show that the distributions of disjointed distances and angles, which include all conformations with various linker lengths, become considerably wider and shift to the higher values, better recapitulating the behaviour of the native chromatin (Figure 2f-h).

Finally, we would like to emphasize the scale and scope of this study. Simulating a system of approximately 3.5 million atoms over a 4 μs timescale represents one of the first all-atom studies of chromatin at this level. Importantly, this work provides a publicly available library of conformations, which can serve as a foundation for further refinement and extended simulations. All simulation trajectories will be deposited into the open-access initiative MDposit https://irb-dev.mddbr.eu/.

Minor comments

Comment 1: Introduction, lines 65 to 70

Recent studies have moved beyond linking nucleosomes by straight DNA linkers (https://www.biorxiv.org/content/10.1101/2025.04.09.647790v1; https://www.biorxiv.org/content/10.1101/2022.08.16.502515v2). It is also not clear what the authors mean by "spatial coherence".

Response: We thank the reviewer for this comment and for highlighting these relevant studies. We have incorporated these works into the revised manuscript. In addition, we have revised the term "spatial coherence" to more explicitly describe the relative positioning and orientation of nucleosomes.

Comment 2: Results, lines 90 to 107

This section mixes presentation of results with extended introduction and partially repeats content already described in the lines 65 to 70.

Response: Per reviewers' suggestion, we have revised these sections.

Comment 3: Results, lines 108 to 120 and Figure 1

Figure 1 is confusing and not sufficient to illustrate the advantages of tri-nucleosome fitting. In Figure 1b, fitting of nucleosomes appears less reliable (in particular for nucleosome N1) compared to the case of single nucleosome template fitting (Figure 1a). Figures 1c and 1d show densities whose shapes are difficult to correlate with nucleosome structures.

Response: We thank the reviewer for this insightful comment. Figure 1 is not intended to suggest that tri-nucleosome fitting is superior to mono-nucleosome fitting in terms of local placement accuracy. It is intended to highlight the distinct conceptual advantages of tri-nucleosome based fitting, namely its ability to preserve the relative order of consecutive nucleosomes and to provide linker DNA geometries that are consistent with physically plausible chromatin conformations. We agree that mono-nucleosome fitting in some cases can achieve more accurate **local** placement of individual nucleosomes but tri-nucleosome fitting yields better quality for the condensed chromatin fiber (Supplementary Figures S4 and S5). Moreover, tri-nucleosome fitting introduces additional structural constraints by enforcing nucleosome connectivity and local chromatin geometry, which cannot be captured by fitting individual nucleosomes independently. Regarding the densities shown in Figures 1c and 1d, we acknowledge that their shapes may not always be easily correlated with individual nucleosome features due to the limited resolution and crowded nature of cryo-ET maps. These panels are intended to illustrate the challenges of interpreting such densities and to demonstrate how tri-nucleosome based fitting provides a more coherent representation of nucleosome arrangements and linker DNA organization under these conditions.

Comment 4: Discussion, lines 275 to 280. This part closely resembles the introduction and does not provide additional discussion or new meaning.

Response: We agree with the reviewer and have revised this part in the revised manuscript.

References

1. Kenzaki H, Takada S. Linker DNA Length is a Key to Tri-nucleosome Folding. *J Mol Biol* **433**, 166792 (2021).
2. Armeev GA, Kniazeva AS, Komarova GA, Kirpichnikov MP, Shaytan AK. Histone dynamics mediate DNA unwrapping and sliding in nucleosomes. *Nat Commun* **12**, 2387 (2021).
3. Morioka S*, et al.* High-Speed Atomic Force Microscopy Reveals the Nucleosome Sliding and DNA Unwrapping/Wrapping Dynamics of Tail-less Nucleosomes. *Nano Letters* **24**, 5246–5254 (2024).
4. Iwasaki W*, et al.* Contribution of histone N-terminal tails to the structure and stability of nucleosomes (vol 3, pg 363, 2013). *Febs Open Bio* **8**, 1567–1567 (2018).
5. Zhou HB*, et al.* Multiscale structure of chromatin condensates explains phase separation and material properties. *Science* **390**, eadv6588 (2025).
6. Jentink N, Purnell C, Kable B, Swulius MT, Grigoryev SA. Cryoelectron tomography reveals the multiplex anatomy of condensed native chromatin and its unfolding by histone citrullination. *Mol Cell* **83**, 3236–3252 (2023).
7. Purnell C*, et al.* Rapid Synthesis of Cryo-ET Data for Training Deep Learning Models. *bioRxiv*, (2023).
8. Kable B*, et al.* Compromised two-start zigzag chromatin folding in immature mouse retina cells driven by irregularly spaced nucleosomes with short DNA linkers. *Nucleic Acids Res* **53**, gkaf457 (2025).
9. Broser PJ*, et al.* Nonlinear anisotropic diffusion filtering of three-dimensional image data from two-photon microscopy. *J Biomed Opt* **9**, 1253–1264 (2004).

RESPONSE TO REVIEWERS' COMMENTS

We sincerely thank the reviewers for their thoughtful and constructive comments. We performed additional analyses and quantitative comparisons using three different denoising methods. All revisions made to the manuscript and the Supplementary Information are highlighted in red.

Responses to reviewer #1

Comment: The authors largely revised the manuscript according to my comments. Especially, additional MD simulations with 2 more lengths of linker DNA broadened the conformational space of tri-nucleosomes markedly. The resulting shows better fitting than the previous one.

I consider the revised manuscript can be published as is.

Response: We thank the reviewer for the positive assessment of our revised manuscript.

Responses to reviewer #2

Comment: I have carefully reviewed the authors' responses to my previous comments as well as the revised manuscript. I find that the authors have addressed all of my concerns thoroughly and satisfactorily. Given the complete readiness of the manuscript, the high quality of the research, and its undeniable relevance, I recommend the manuscript for publication in its current form.

Response: We appreciate the reviewer's recognition that the previous concerns have been thoroughly and satisfactorily addressed, and we are grateful for the positive recommendation for publication.

Responses to reviewer #3

Comment: The authors have done an excellent job of addressing all my concerns, and the revised manuscript is excellent. I am very supportive of this revised version being published as it is.

Response: We thank the reviewer for the encouraging evaluation of our revised manuscript.

Responses to reviewer #4

Comment: I thank the authors for addressing my specific comments. However, regarding their response to my major point, in my opinion, additional validation is still required.

The current best practices in cryo-ET data processing should not be overlooked. Cryo-ET has several well-known limitations, particularly high noise levels and the missing wedge (MW).

Cross-correlation, which is used in this manuscript as the fitting metric, is calculated on the basis on pixel intensity values. The application of non-linear filters, such as NAD or deep-learning-based networks, modifies pixel values while enhancing contrast in a way that cannot be fully predicted and taken into an account. As a result, such processing may artificially increase the cross-correlation score and thereby influence the fitting outcome. The previous publication referenced in the rebuttal https://doi.org/10.1093/nar/gkaf457 (Supplementary Figures S1BC) compares two nonlinear filters with each other, but not with raw data or with raw data with applied standard linear filters, such as a Fourier band-pass filter, which is standard in cryo-ET data processing.

The missing wedge has the most severe impact, resulting in artificial elongation of structures along the electron beam axis and, consequently, prone of an artificial increase in the volume of particles making fitting more permissive and less precise. Application of non-linear filters to increase the contrast may further enhance this effect.

The Chimera cross-correlation-based fitting would only be justifiable if the EM data have a uniform resolution and without non-linear filters applied, for example SPA maps or sub-tomogram averages. In case of raw cryo-ET data the quantitative assessment of the fitting accuracy in relation to missing wedge and contrast enhancement should be provided.

This evaluation does not necessarily require recalculating the fitting for all MD frames, which I understand would be computationally heavy. It could be initially performed on the obtained best-fits and include two components:

(i) assessment of the influence of the missing wedge and the application of nonlinear denoising filters on the position and orientation of the fitted tri-nucleosome template.

(ii) determination the translational and angular differences between nucleosome positions and orientations obtained by mono-nucleosome template fitting (with MW taken into account and without non-linear contrasting) and the positions and orientations of the same nucleosomes resulted from tri-nucleosome template fitting.

To help readers to be aware of the impact of the missing wedge, the Figures showing representative fitting results for mono- and tri-nucleosomes should also include XZ views of the cryo-ET densities.

Response: We thank the reviewer for the valuable suggestions. In response to the reviewer's comments, we would like to clarify that in this work we used maps that had already been denoised in a previous study *(Jentink et al, Mol Cell 2023)*. In that later study, we discussed in detail several limitations resulting from the nature of Cryo-ET imaging of highly variable chromatin particles, including those caused by inherent noise and the missing wedge artifact. Still, by stereological and statistical analyses of multiple nucleosome arrays (many more than in the present study), we found no evidence of nonrandom bias. The extremely high variability of native chromatin conformations revealed by that study (and, since then confirmed by a number of *in situ* Cryo-ET studies) precludes post-tomogram averaging reconstruction of any structures beyond the mono-nucleosome. As to the correlation coefficient, we did use another quality-of-fit measure "inside-contour".

As further suggested by the reviewer, we have included an additional analysis to evaluate the effect of non-linear denoising filters on the fitting results. Specifically, we performed quantitative comparisons using a representative tomogram for the particles used in this study processed with: NAD-eed-3d filter in IMOD that was used for large-scale stereological analysis (*Jentink et al, Mol Cell 2023*), deep learning-based denoising that was used to enhance rendering of nucleosomal DNA and to reveal nucleosome connectivity by the linker DNA (*Jentink et al, Mol Cell 2023*), and Gaussian filtering of raw tomograms (*Frangakis et al, J Struct Biol 2021*). The NAD filter and the deep learning-based denoising are non-linear filtering methods, whereas Gaussian filtering is a linear filtering method. No significant differences were observed in either the orientations or the positions of the fitted structural models across the three methods (Supplementary Figure S9). The corresponding raw data has also been provided for reference (Supplementary Table 4). These new analyses have been incorporated into the revised manuscript. We also added a discussion of the limitations associated with the missing wedge and imperfect denoising in the revised manuscript.

In addition, as requested by the reviewer, we included a new Supplementary Figure S2 to show the representative fitting results of mono- and tri-nucleosomes with the corresponding XZ views of the cryo-ET density maps. Accordingly, the original Supplementary Figures S2–S23 have been renumbered as Supplementary Figures S3–S8 and S10–S25 in the revised manuscript and Supplementary Information.

References

1. Jentink N, Purnell C, Kable B, Swulius MT, Grigoryev SA. Cryoelectron tomography reveals the multiplex anatomy of condensed native chromatin and its unfolding by histone citrullination. Mol Cell 83, 3236–3252 (2023).
2. Frangakis AS. It's noisy out there! A review of denoising techniques in cryo-electron tomography. J Struct Biol 213, 107804 (2021).

RESPONSE TO REVIEWERS' COMMENTS

Responses to reviewer #4

Comment: The authors have adequately addressed all of the comments I raised. I recommend publication of the manuscript as it is.

Response: We appreciate the reviewer's recognition that all of the comments have been addressed, and we are grateful for the positive recommendation for publication.
